# Human Milk Metabolomics Are Related to Maternal Adiposity, Infant Growth Rate and Allergies: The Chinese Human Milk Project

**DOI:** 10.3390/nu14102097

**Published:** 2022-05-18

**Authors:** Wei Zhang, Kaifeng Li, Chengdong Zheng, Han Sun, Jiancun Pan, Yuanyuan Li, Ying Liu, Wenqing Wang, Mengnan Ju, Yajun Xu, Shilong Jiang

**Affiliations:** 1 Nutrition and Metabolism Research Division, Innovation Center, Heilongjiang Feihe Dairy Co., Ltd., C-16, 10A Jiuxianqiao Rd., Chaoyang, Beijing 100015, China; zhangwei1@feihe.com (W.Z.); likaifeng@feihe.com (K.L.); zhengchengdong@feihe.com (C.Z.); sunhan@feihe.com (H.S.); panjiancun@feihe.com (J.P.); liyuanyuan3@feihe.com (Y.L.); liuying3@feihe.com (Y.L.); wangwenqing@feihe.com (W.W.); jumengnan@feihe.com (M.J.); 2 PKUHSC-China Feihe Joint Research Institute of Nutrition and Healthy Lifespan Development, Xueyuan Road 38, Haidian, Beijing 100083, China; 3 Department of Nutrition and Food Hygiene, School of Public Health, Peking University, Xueyuan Road 38, Haidian, Beijing 100083, China; 4 Beijing Key Laboratory of Toxicological Research and Risk Assessment for Food Safety, Peking University, Xueyuan Road 38, Haidian, Beijing 100083, China

**Keywords:** metabotype, eczema, brown adipose tissue, thermogenesis, ambient temperature

## Abstract

The metabolomic profiles of Chinese human milk have been poorly documented. The objective of the study was to explore associations between human milk metabotypes, maternal adiposity, infant growth patterns, and risk of allergies. Two hundred mother–infant dyads from seven cities were randomly selected from the Chinese Human Milk Project (CHMP). Untargeted human milk metabolomic profiles were determined using HPLC-MS/MS. Two human milk metabotypes were identified using principal component analysis. Principal component (PC) 1 was characterized by high linoleic acid metabolites with low purine nucleosides and metabolites of glutamate and glutathione metabolism. PC 2 was characterized by high glycerophospholipids and sphingomyelins content. Higher PC1 scores were associated with slower infant growth rate and higher ambient temperature (*p* < 0.05). Higher PC 2 scores were related to higher maternal BMI and increased risk of infant allergies (*p* < 0.05). Future work is needed to understand the biologic mechanisms of these human milk metabotypes.

## 1. Introduction

The Developmental Origins of Health and Disease (DOHaD) establishes early postnatal life as a window of susceptibility in which the effects of nutrition, growth, and development could influence later life. It is believed that breastfeeding grants neonates with the optimal trajectory of growth and privileges in immune functions.

Prolonged breastfeeding has been shown to be beneficial for certain outcomes in children, such as the ability to fight infections, malocclusion, and intelligence, while definite evidence has not yet been established for others, including childhood obesity and allergies [1]. Childhood obesity is recognized as the one of the most serious global public health challenges of the 21st century by the World Health Organization, with the rate of childhood and adolescent obesity increasing over ten-fold during the last four decades. In addition to childhood obesity, eczema is a common childhood allergic condition which affects 20% of children. Children with eczema are prone to develop other atopic conditions, including food allergies, asthma, and allergic rhinitis [2]. Although it has been well documented that breastfeeding is associated with a lower risk of excessive weight gain, the relationship between breastfeeding and childhood obesity remains inconclusive [3]. Similarly, mixed results have been reported regarding the effects of breastfeeding on the risk of developing allergies [4,5].

One possible reason for the aforementioned inconclusive results may be the presence of the metabolic heterogeneity of human milk composition which leads to substantial unexplained variance in infants [6]. It has been argued that the metabotypes (metabolic phenotypes) which categorize individuals to homogenous subgroups based on a combination of their metabolic characteristics may be able to reduce interindividual variability and enhance the ability of detecting significant differences between subgroups [7]. Metabotypes of biological systems, such as the plasma and urine, have been investigated previously to offer novel insights into health science research [8]. Recently, we derived human milk patterns from the integrative analysis of proteomic, lipidomic and glycomic datasets that were related to infant growth rates and allergies [8]. The advantages of human milk largely arise from its complex composition including sufficient amounts of nutrients, as well as non-nutritive substances such as metabolites [5]. In recent years, several studies have reported that maternal factors such as gestational diabetes and adiposity alter the metabolites in the milk [9]. More importantly, the association between human milk metabolites and infant growth has been suggested [5]. Unfortunately, little is known in terms of what metabotypes can be identified from mature human milk metabolites and how those metabotypes are associated with infant growth rate and the risk of allergies.

Against this backdrop, the objective of the study was to identify metabotypes from mature human milk metabolomic profiles and to investigate the association between human milk metabotypes, maternal adiposity, infant growth, and the risk of allergies based on the CHMP.

## 2. Materials and Methods

### 2.1. The Chinese Human Milk Project

The CHMP study is a cross-sectional study which recruited 1800 participants from China to evaluate HM composition in a Chinese population (NCT03675204). Healthy lactating mothers aged 25 to 35 years who were breast-feeding infants of 15 to 180 days, and who were not smokers or alcohol drinkers, were recruited in the study. The exclusion criteria included: mothers who were being treated for gastrointestinal symptoms; mothers who were suffering from mastitis, infectious disease, cardiovascular disease, metabolic disease, mental system disease, cancer, or other malignant diseases; mothers who had a history of taking antibiotics; mothers who were not able to answer the study questions; and mothers who had participated in any nutritional or pharmaceutical intervention study recently.

### 2.2. Sample Collection Protocol

Participants were asked to collect a HM sample at 9:00–11:00, at least 1.5 h from the last lactation in the morning. An electric pump was used to express one breast fully. The milk collected was then carefully weighted using a calibrated scale. Ambient temperatures (T_a_) of the collection date and city were obtained from the website of China Meteorological Administration. As the meteorological temperature of cities from different regions vary significantly in China, the temperatures were related to potential cold exposure or heat stress during maternal daily outdoor activities. The milk was gently mixed to ensure homogenization and then aliquoted to 6 × 5 mL tubes; the remaining samples were aliquoted to 10 mL tubes as needed. Aliquoted samples were delivered to our laboratory using dried ice before being stored at −80 °C. A questionnaire to identify maternal and infant characteristics was given to each participant. The study was approved by the review board of Shanghai Nutrition Society Ethics Committee (Ethic Approval [2016] No. 006). Informed written consent was obtained from all participants. The present study was based on the CHMP study: 200 mother–infant dyads in the second and sixth months of lactation were randomly selected from seven cities (Guangzhou, Weihai, Chengdu, Beijing, Jinhua, Lanzhou and Zhengzhou).

### 2.3. Maternal and Infant Characteristics

Maternal education, occupation, parity, delivery mode, family annual income, pre-pregnancy weight, sleeping duration, and smoking and drinking habits were assessed by the questionnaire. Eczema was estimated using a self-reported questionnaire by asking if eczema had ever occurred. Maternal postpartum body weight and height were measured upon sampling by calibrated electronic scales while participants were only wearing indoor clothing and no shoes. Weight and length of the infant were measured using a tared scale and a length board with a sliding foot piece, respectively. The sampling season was defined according to the sample collection date, as follows: spring (March to May), summer (June to August), autumn (September to November) and winter (December to February).

### 2.4. Feature Selection and Principal Component Analysis (PCA)

A total of 4301 metabolites were detected in all the samples, with 525 metabolites being annotated using the in-house MS2 database. In general, relative standard deviation (RSD %) of the three IS of the QC samples were 3.6%, 5.0% and 5.1%, respectively. The coefficient of variation (CV) for all metabolites was calculated from 21 quality control samples (ranging from 1.6% to 29.9%), and metabolites with a CV over 25% (*n* = 37) were excluded from further analysis. To minimize the possible noise of this high-dimensional metabolomics data, metabolites with an RSD less than 25% (*n* = 49) were further excluded, leaving a final metabolite number of 439. The data were log-transformed and Pareto-scaled and then fitted to the PCA by *FactoMineR* R package [10]. The PC score of each sample, the correlation between the metabolites and the factors, and contributions of each metabolite were calculated. *p*-values of correlation between the features and factors were adjusted using the Benjamini–Hochberg method [11]. To decide the number of PCs and to the evaluate stability of each PC, one thousand bootstrap samples were drawn and PCA was performed for each bootstrap sample. The mean, SD, and 95% bootstrap confidence interval (CI) of the loadings of all bootstrap samples were calculated and compared with original factor correlation. The number of PCs was chosen based on the scree plot, eigenvalue and stability of each PC. Each PC was characterized by metabolites with an absolute original loading above 0.5.

### 2.5. Statistical Analysis

Length-for-age *z* scores (LAZ), weight-for-age *z* scores (WAZ), BMI-for-age *z* scores (BAZ) and weight-for-length (WFL) *z* scores were calculated based on the WHO Child Growth Standards [12]. Multiple linear regression, adjusted for infant age, infant sex, birth weight, birth length, maternal age, and city were carried out to explore the associations between PC scores, infant growth *z* scores, and maternal BMI. Odds ratios of allergies against factor loadings were calculated using the generalized linear model with a further adjustment of the infant feeding pattern (breastfeeding vs. breastfeeding + formula feeding). All the regression analyses were performed using R (version 4.0.3) [13]. The mediation model was used to explore the mediation effects of human milk metabolite PCs in the maternal postpartum BMI and the risk of allergies using Mplus (version 8.3) [14]. Covariates including infant age, infant sex, maternal age, and city were added to account for potential treatment–outcome and mediator–outcome confounding factors. For binary outcome (allergies), the counterfactual model was used to estimate the potential total natural indirect effects of maternal BMI on the risk of allergies through HM metabotypes. An independent sample *t*-test was used to identify the differentially expressed metabolites (DEMs) between the samples collected in the summer (*n* = 20) and winter (*n* = 57). *p*-values obtained from the *t*-test were adjusted using the Benjamini–Hochberg method [11]. The fold changes of metabolites (summer vs. winter) were also calculated. Metabolites with fold changes over 1.5 and adjusted *p*-values lower than 0.05 were defined as DEMs. Multiple linear regression adjusted *for infant age*, *sex*, *maternal age*, *and city* were carried out to explore the association between weight of milk collected, PC scores, ambient temperature, WAZ, and LAZ.

## 3. Results

In general, 200 participants (of which 196 were primiparous with mean maternal age of 30.0 ± 5.4 years and self-reported gestational weight gain of 14.2 ± 5.4 kg) from seven cities of China were included in this analysis (Table 1). Over half of the infants (57.0%) were boys and mean (SD) birth weight and length were 3361 (659) grams and 50.1 (2.4) cm, respectively.

### 3.1. Characteristics of HM Metabotypes

**Figure 1:**
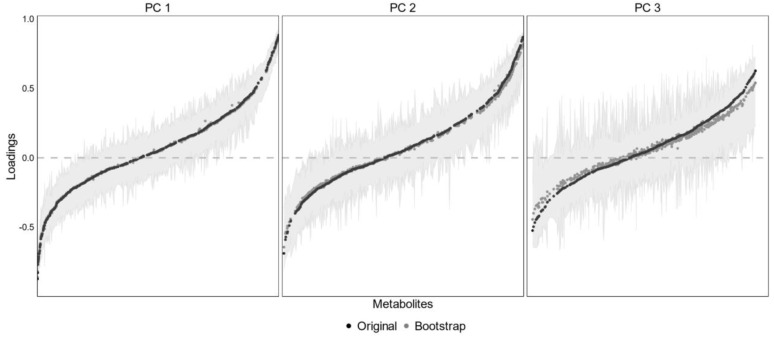
PCA loadings of bootstrap samples.

The characteristics of the HM metabotypes are presented in Figure 1, Tables S1 and S2. PC 1 was positively associated with linoleic acid (LA) metabolism derivatives (including arachidonic acid (ARA), 13S-hydroxyoctadecadienoic acid (13(s)-HPODE), 12,13-epoxy-9-octadecenoic acid (12,13-EpOME), and α-dimorphecolic acid (9(s)-HODE)), ARA metabolism derivatives (including ARA and Δ-12-Prostaglandin J_2_), and a α-linolenic acid metabolism derivative (colnelenic acid) (Table S1). In contrast, PC 1 was negatively associated with purine derivatives (including adenine, adenosine, and 5-hydroxyisourate), and glutamate and glutathione metabolism (D-glutamine, pyroglutamic acid, 4-hydroxy-L-glutamic acid and N2-gamma-Glutamylglutamine). In addition to adenine and adenosine, PC 1 was also negatively (although with weaker loadings: see Table S3) associated with guanine (loading = −0.38) and guanosine (loading = −0.37). Interestingly, PC 1 also showed some characteristics related to bile acid metabolism, including a positive loading for cholesterol (0.54) and a negative loading for 5beta-Cholestane-3alpha,7alpha,24,26-tetrol (5BCT, −0.56), which is a bile acid lipid molecule. In terms of PC 2, positive associations were observed for a group of phospholipids (PL), including glycerophospholipids (mostly phosphatidylethanolamines and phosphatidylcholines) and sphingomyelins (SMs) (Table S2). Notably, region-specific effects were observed (Figure S1A): samples from eastern China tended to cluster together.

To evaluate the robustness of each PC obtained using PCA, 1000 bootstrap samples were drawn and PCA was performed over each bootstrap sample. The bootstrap loadings were averaged and plotted as shown above. The original PCA loadings are colored in black, while mean bootstrap loadings are colored in grey. Light grey areas represent a 95% bootstrap CI, calculated using 1000 bootstrap samples.

### 3.2. Regression and Mediation Analysis

The association between HM metabotypes, maternal BMI and infant growth z-score are shown in Table 2. Higher scores of PC 1 were associated with lower infant WAZ (*β* = −0.06, *p* = 0.004) and LAZ (*β* = −0.08, *p* = 0.004) after adjustment for infant age, sex, city, maternal age, birth weight, and birth length. However, PC 2 was positively associated with maternal postpartum BMI (*β* = 0.08, *p* = 0.018) and the risk of allergies in infants (*OR* = 1.07, *p* = 0.019) after adjustment for infant age, sex, city, maternal age, birth weight, and birth length. The results of the mediation analysis are presented in Figure S2. In general, higher maternal postpartum BMI was directly (*OR* = 1.24, 95% CI: [1.01, 2.26]) related to a higher risk of allergies. Those associations seem to be sex-specific, with associations only significant for boys when analyses were stratified by sex (Table S6). When the mediation effect of PC 2 was considered, the total effect (*OR* = 1.29, 95% CI: [1.03, 2.42]) was also significant; however, the lower 95% CI of *OR* of natural indirect effect was slightly below 1.000 (0.996), suggesting a non-significant indirect effect.

### 3.3. Association between Ambient Temperature, Season and Human Milk Metabolomic Profile

**Figure 2:**
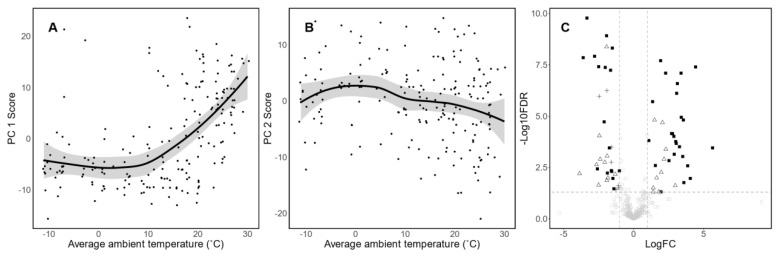
Association between ambient temperature, season, and human milk metabolomic profile. (**A**) Association between average ambient temperature and PC 1 score of each sample collected. Grey area represents *se* of estimates. PC 1 score was positively associated (*β* = 0.34, *p* = 1.29 × 10^−14^) with ambient temperature based on multiple linear regression adjusting for infant age, sex, and maternal age. (**B**) Association between average ambient temperature and PC 2 score of each sample collected. Grey area represents *se* of estimates. PC 2 score was negatively associated (*β* = −0.13, *p* = 0.002) with ambient temperature based on multiple linear regression adjusting for infant age, sex, and maternal age. (**C**) Volcano plot of differentially expressed metabolites between samples collected in summer and in winter. ○—metabolites that were not differentially expressed; ∆—differentially expressed metabolites according to *t*-test and fold change; +—differentially expressed metabolites which were overlapped with characteristics of PC 2; ▪—differentially expressed metabolites which were overlapped with characteristics of PC 1.

The association between average T_a_ and PCA scores are presented in Figure 2A,B. In general, PC1 scores were positively associated (*β* = 0.34, *p* = 1.29 × 10^−14^) with average T_a_ after adjustment for infant age, sex, and maternal age. A sharp increase in PC1 score was observed when T_a_ exceeded 10 °C (Figure 2A). In contrast, a mild decrease in PC2 score (*β* = −0.13, *p* = 0.002) was observed as T_a_ increased (Figure 2B). An independent sample *t*-test was then used to explore the DEMs between samples collected in the summer (*n* = 20) and the winter (*n* = 57), with 70 metabolites being identified (Figure 2C and Table S7). Importantly, 42 out of those 70 DEM overlapped with the PC1 profile (Figure 2 and Table S8), including purine and nucleoside, LA metabolites, bile acid, and glutamate metabolites. In addition, 10 PLs were identified in both the DEMs and the PC 2 profile (Figure 2 and Table S9).

## 4. Discussion

Two robust HM metabotypes were identified which were related to maternal BMI, infant growth rate, and risk of eczema in our study. In general, a HM metabotype (PC 1) characterized with high LA metabolites and low purine nucleosides (adenosine, adenine, guanosine and guanine) and glutamate derivatives was associated with a slower infant growth rate. On the other hand, PC 2 with abundant PLs and SMs was related to maternal BMI and an increased risk of eczema.

Human milk PC 1 identified in our study showed clear characteristics related to reduced cold-induced thermogenesis and increased heat stress. First of all, cold exposure induces the hepatic conversion of cholesterol to bile acids, which results in increased heat production [15]. Additionally, the oral administration of bile acid has been found to increase human BAT activity [16], while heat stress reduces the bile acid secretion of Holstein cows [17]. This is in line with our study, which found that PC 1 showed positive loading for cholesterol and negative loading for bile acid metabolite. Secondly, acute and chronic cold exposure circulated 12,13-dihydroxy-9Z-octadecenoic acid (12,13-diHOME) concentration, which further induces BAT fuel uptake and thermogenesis [17]. The circulating levels of 12,13-diHOME were also negatively correlated with BMI and insulin sensitivity [18]. This coincides with our results; we found that PC 1 had positive loadings for a group of LA metabolites (including 12,13-EpOME, which is the precursor of 12,13-diHOME). Lastly, BAT thermogenesis is mainly regulated by uncoupling protein 1 (UCP1), whose activity is inhibited by purine nucleotide and activated by long chain free fatty acids [19]. Recent studies have also reported that the cold-induced thermogenesis of BAT decreases free purine nucleotides (ATP, ADP and GTP) and increases its respective degradation products [20,21]. This is in agreement with the PC 1 profile, which presented negative loadings for purine nucleosides and glutamate/glutathione derivatives. Taken together, it is reasonable to speculate that human milk samples with a high score of PC 1 were collected under warmer temperature and, therefore, had a lower degree of BAT thermogenesis. This hypothesis is further supported by the finding that PC 1 score was positively associated with ambient temperature (Figure 2A), and by the fact that DEM overlapped with PC1 metabolites by 60% (Table S8).

Moreover, both PC 1 score (*β* = −0.51, *p* = 0.007) and average Ta (*β* = −0.92, *p* = 0.022) were negatively associated with the weight of milk collected from mothers after adjustment for infant age, sex, maternal age, and city. Additionally, the weight of milk collected was positively associated with WAZ (*β* = 0.01, *p* = 0.032), but not LAZ (*β* = 0.01, *p* = 0.210) (Table S10). These results may well be explained by the heat dissipation limit (HDL) theory [22], which suggests that the lactation performance of females largely depends on their heat dissipation ability. The HDL theory is supported by observations that animals (such as mice, swine, and cows) exposed to high ambient temperatures (heat stress) produced less milk [23,24]. In contrast, cold exposure facilitates heat dissipation and, therefore, elevates milk production and weans larger pups [25]. Therefore, it seems that a lower PC 1 score in our study was an indicator of lower Ta, which resulted in higher milk expression and, subsequently, the faster growth rate of infants.

In recent years, great attention has been paid regarding the effects of brown adipose tissue (BAT) and beige adipose tissue (BeAT) thermogenesis on the risk of obesity [19,26]. BAT is abundant in human neonates and helps infants to maintain a normal body temperature. Additionally, BAT decreases while white adipose tissue increases as infants develop [27]. This process may be pivotal, as BAT thermogenesis is important in terms of energy expenditure and increased white adipocyte number is positively associated with early childhood obesity [27,28]. A recent study reported that human milk alkylglycerols maintained beige adipose tissue in infants and prevented the transdifferentiation of beige adipose tissue into lipid-storing white adipose tissue [29]. However, how maternal BAT activity may affect infant adipocyte transformation and infant growth remain largely unknown. Future studies are needed to further characterize the potential crosstalk between human milk metabolites and infant adipocyte transformation.

In our study, human milk PC 2 (characterized by high SM and PL content) was related to the increased risk of allergic events. It has been reported that phospholipids facilitate the intestinal uptake of food allergens by protecting them from gastrointestinal degradation, which may promote allergic processes [30]. This is supported by studies reporting that PC inhibits the digestion of milk allergens β-lactoglobulin and α-lactalbumin which, in turn, leads to the increased allergenic activities of those allergens [31,32]. Our previous study also showed that human milk patterns with lower allergen αs_1_-casein and PLs were associated with a lower risk of allergies [8]. It is therefore possible that higher levels of human milk phospholipids may increase the likelihood of allergies caused by allergens, which merits future investigation. Importantly, the association between PC2 and risk of eczema was only significant for boys (Table S6), which is in line with previous studies showing that boys are more prone to allergies (eczema and asthma) than girls [33,34]. This sex-specific effect may be related to hormonal influences [33].

Interestingly, maternal BMI was negatively associated with PC 2 in our study. Even though we are not aware of any study that has reported such an association between maternal adiposity and HM lipidomic profile, accumulating evidence suggests that plasma SMs and some PLs are significantly higher in obese subjects [35,36,37]. Furthermore, our study showed that higher maternal BMI was related to increased risk of eczema (*OR* = 1.14, *p* < 0.05) after adjustment for infant sex, city, lactation stage, and maternal age. This is supported by numerous studies reporting that maternal adiposity may increase the risk of allergic diseases in infants [38,39,40]. Taken together, we hypothesize that maternal BMI may alter human milk PL and SM compositions, which may further translate into increased the risk of allergic events in the infant. A mediation model was then applied to test this hypothesis; however, the indirect effects of PC2 on the relationship between maternal BMI and the risk of eczema was just below the boundary of significance (*OR* = 1.04, 95% bootstrap CI: 0.996 to 1.142, Figure S2). It might be that the sample size of the present study was not sufficient to capture such an effect, which calls for future investigation.

The limitation of our study is the cross-sectional study design, which only reveals correlation rather than causality. It should be pointed out that maternal BAT activity was not directly determined in our study and future work is needed to validate our observations. In addition, maternal dietary intakes were not assessed, which may affect both human milk composition and BAT activity. Another limitation is that human milk components other than metabolites (such as proteomics) were not assessed, and these may also be involved in the overall metabolism of human milk. Lastly, meteorological temperature rather than room temperature was used in our study, which is a potential limitation as room temperature is directly linked to thermogenesis.

## 5. Conclusions

We identified two robust human milk metabotypes that were associated with either slower infant growth rate or increased risk of allergic events. PC 1 was associated with high linoleic acid metabolites and low purine nucleosides, as well as the metabolites of glutamate and glutathione metabolism. PC 2 was characterized by a high glycerophospholipids and sphingomyelins content. Higher PC1 scores were associated with slower infant growth rate and a higher ambient temperature. Higher PC 2 scores were related to higher maternal BMI and an increased risk of infant allergies. Future work is needed to understand the biologic mechanisms of these human milk metabotypes and to generalize them to other populations.

## Figures and Tables

**Table 1:** Maternal and infant characteristics (*n* = 200).

-	Total (*n* = 200)	
Maternal Characteristics	Mean	SD
Age (years)	30.0	5.4
BMI (kg/m^2^)	22.8	2.8
Gestational weight gain (kg)	14.2	5.4
Parity		
1	196 (98.5%)	
2	3 (1.5%)	
City		
Beijing	27 (13.5%)	
Chengdu	29 (14.5%)	
Guangzhou	25 (12.5%)	
Jinhua	30 (15.0%)	
Lanzhou	36 (18.0%)	
Weihai	20 (10.0%)	
Zhengzhou	33 (16.5%)	
Infant characteristics		
Sex (*n*)		
Male	114 (57.0%)	
Female	86 (43.0%)	
Birth weight (gram)	3361.0	659.1
Birth length (cm)	50.1	2.4
Age		
2 mo	106 (53.0%)	
6 mo	94 (47.0%)	
Self-reported eczema, Yes	63 (31.5%)	
*z* Score		
WAZ	0.57	1.73
LAZ	0.14	2.09
BAZ	0.74	2.27
WFL	0.90	2.50

WAZ—weight-for-age *z* score; LAZ—length-for-age *z* score; BAZ—BMI-for-age *z* score; WFL—weight-for-length *z* score.

**Table 2:** Association between human milk PC scores, infant growth, and risk of eczema (*n* = 200).

		PC 1		PC 2	
-	-	Estimates	*p* Value	Estimates	*p* Value
Maternal BMI (kg/m^2^)	Model 1	−0.03	0.471	0.08	0.018
	Model 2	−0.03	0.497	0.08	0.018
BAZ	Model 1	−0.02	0.472	0.00	0.996
	Model 2	−0.03	0.344	−0.01	0.666
WAZ	Model 1	−0.06	0.007	0.01	0.605
	Model 2	−0.06	0.004	4.9 × 10^−3^	0.795
LAZ	Model 1	−0.09	0.001	0.014	0.554
	Model 2	−0.08	0.004	0.019	0.412
Eczema	Model 1	0.96	0.241	1.06	0.032
	Model 2	0.97	0.306	1.07	0.019

Estimates for allergies are presented as odds ratios. Model 1 adjusted for infant age, sex, maternal age, and city; model 2 adjusted for infant age, sex, city, maternal age, birth weight, and birth length. Further adjustments to infant feeding pattern (breastfeeding vs. breastfeeding + formula feeding) were added for risk of eczema. WAZ—weight-for-age *z* score; LAZ—length-for-age *z* score; BAZ—BMI-for-age *z* score.

## Data Availability

The raw data supporting the conclusions of this article will be made available by the authors, without undue reservation.

## Supplementary Materials

The following are available online at https://www.mdpi.com/article/10.3390/nu14102097/s1, Table S1: Characteristics of human milk PC 1; Table S2: Characteristics of human milk PC 2; Table S3: Details of PC 1 results; Table S4: Details of PC2 results; Table S5: Details of PC3 results; Table S6: Association between human milk PC scores, infant growth and risk of eczema by sex (*n* = 200); Table S7: Differentially expressed human milk metabolites between samples collected in summer and winter (*n* = 77); Table S8: Overlapped human milk metabolites being identified from both PC 1 and *t*-test; Table S9: Overlapped human milk metabolites being identified from both PC 2 and *t*-test; Table S10: Association between weight of milk collected, PC scores, ambient temperature, and infant *z* scores; Figure S1: PCA score plot of human milk metabolites; Figure S2: Mediation analysis between human milk PC scores, maternal postpartum BMI, and risk of allergies; 2 Materials and Methods.
