# Supplementary material for: Human Milk Metabolomics Are Related to Maternal Adiposity, Infant Growth Rate and Allergies: The Chinese Human Milk Project
Source: Nutrients. 2022 May 18;14(10):2097. doi: 10.3390/nu14102097 (PMC9144552; doi:10.3390/nu14102097)
Supplement: Supplementary file 1 [file nutrients-14-02097-s001.zip › Nutrients_Supplementary_Material-2022 05 07.pdf]

Table S1. Characteristics of human milk PC 1.

Metabolites	Class	PCA loadings	Bootstrap 95% CI of loadings		Contribution (%)	FDR
			Lower	Upper		
4-Hydroxy-L-glutamic acid	Organic acids and derivatives	-0.87	-0.91	-0.81	1.56	3.76E-61
Biliverdin	Tetrapyrroles and derivatives	-0.83	-0.87	-0.74	1.82	4.46E-50
Glaucarubin	Prenol lipids	-0.77	-0.82	-0.69	1.46	5.18E-39
Melleolide M	Prenol lipids	-0.75	-0.81	-0.65	1.49	2.07E-36
N2-gamma-Glutamylglutamine	Carboxylic acids and derivatives	-0.75	-0.80	-0.67	1.08	1.02E-35
5-Hydroxyisourate	Imidazopyrimidines	-0.73	-0.81	-0.60	3.81	7.16E-34
D-Glutamine	Carboxylic acids and derivatives	-0.73	-0.81	-0.58	0.76	3.39E-33
Dihydromaleimide	Pyrrolines	-0.71	-0.80	-0.55	0.46	3.33E-31
MG(0:0/22:4(7Z,10Z,13Z,16Z)/0:0)	Glycerolipids	-0.69	-0.78	-0.56	0.85	7.52E-29
Sonchuionoside C	Prenol lipids	-0.69	-0.80	-0.57	1.02	3.81E-28
N-Methylnicotinamide	Pyridines and derivatives	-0.67	-0.75	-0.56	0.45	8.65E-27
Pyroglutamic acid	Carboxylic acids and derivatives	-0.67	-0.78	-0.51	2.21	2.14E-26
PC(18:3(6Z,9Z,12Z)/15:0)	Glycerophospholipids	-0.64	-0.79	-0.44	0.47	8.69E-24
Adenosine	Purine nucleosides	-0.63	-0.74	-0.50	0.63	8.81E-23
Citrulline	Carboxylic acids and derivatives	-0.63	-0.73	-0.46	0.32	1.73E-22
Pyrrolidonecarboxylic acid	Carboxylic acids and derivatives	-0.62	-0.75	-0.45	0.26	6.72E-22
1-(Malonylamino)cyclopropanecarboxylic acid	Carboxylic acids and derivatives	-0.58	-0.73	-0.35	0.46	8.86E-19
5'-Methylthioadenosine	5'-deoxyribonucleosides	-0.57	-0.68	-0.49	0.39	9.50E-18
PE(16:0/20:4(5Z,8Z,11Z,14Z))	Glycerophospholipids	-0.56	-0.70	-0.37	0.43	3.66E-17
Adenine	Imidazopyrimidines	-0.56	-0.66	-0.40	0.42	3.88E-17
5beta-Cholestane-3alpha,7alpha,24,26-tetrol	Steroids and steroid derivatives	-0.56	-0.68	-0.38	0.43	6.42E-17
5-Hydroxy-7-methoxy-2-tritriacontyl-4H-1-benzopyran-4-one	Benzopyrans	-0.56	-0.73	-0.37	0.53	7.30E-17
PE(22:5(4Z,7Z,10Z,13Z,16Z)/16:0)	Glycerophospholipids	-0.55	-0.72	-0.34	0.36	2.40E-16

Lacto-N-biose I	Organooxygen compounds	-0.55	-0.65	-0.40	0.24	2.91E-16
Dihydrouracil	Diazines	-0.52	-0.65	-0.32	0.15	1.30E-14
3-Methylguanine	Imidazopyrimidines	-0.51	-0.66	-0.28	0.28	4.43E-14
D-Maltose	Organooxygen compounds	-0.51	-0.64	-0.31	0.18	6.67E-14
Prolyl-Arginine	Carboxylic acids and derivatives	0.50	0.37	0.61	0.79	3.02E-13
beta-Lactose	Carbohydrates and carbohydrate conjugates	0.50	0.35	0.62	0.59	1.94E-13
Cytidine	Pyrimidine nucleosides	0.51	0.32	0.65	0.30	6.67E-14
Cholesterol	Steroids and steroid derivatives	0.54	0.40	0.66	0.58	1.35E-15
Niacinamide	Pyridines and derivatives	0.56	0.40	0.68	0.38	2.51E-17
PC(22:2(13Z,16Z)/P-18:0)	Glycerophospholipids	0.57	0.41	0.67	0.41	5.18E-18
Ethyl trans-p-methoxycinnamate	Cinnamic acids and derivatives	0.63	0.50	0.71	2.06	2.53E-22
Phenol glucuronide	Organooxygen compounds	0.63	0.48	0.72	0.50	1.88E-22
1-Methyl-1,3-cyclohexadiene	Olefins	0.63	0.49	0.73	0.32	1.07E-22
LysoPE(16:0/0:0)	Glycerophospholipids	0.63	0.50	0.73	0.57	8.89E-23
o-Xylene	Benzene and substituted derivatives	0.65	0.51	0.74	0.40	2.70E-24
LysoPC(P-16:0)	Glycerophospholipids	0.67	0.52	0.78	0.61	2.14E-26
Dihydro-3-(1-octenyl)-2,5-furandione	Oxolanes	0.68	0.54	0.78	0.66	6.19E-28
LysoPC(16:0)	Glycerophospholipids	0.69	0.57	0.77	0.73	4.02E-28
5,12-dihydroxy-6,8,10,14,17-eicosapentaenoic acid	Fatty Acyls	0.69	0.56	0.77	2.04	7.63E-29
3-Methyl-5-pentyl-2-furanundecanoic acid	Fatty Acyls	0.69	0.58	0.78	0.52	7.10E-29
DG(22:5(7Z,10Z,13Z,16Z,19Z)/16:1(9Z)/0:0)	Glycerolipids	0.69	0.56	0.79	0.79	6.18E-29
MG(0:0/18:4(6Z,9Z,12Z,15Z)/0:0)	Glycerolipids	0.71	0.59	0.79	1.05	1.32E-30
DG(22:4(7Z,10Z,13Z,16Z)/18:3(6Z,9Z,12Z)/0:0)	Glycerolipids	0.71	0.60	0.81	1.08	1.06E-30
Lactapiperanol D	Prenol lipids	0.72	0.60	0.79	1.09	1.16E-31
Styrene	Benzene and substituted derivatives	0.74	0.61	0.83	0.50	3.09E-34

Capsiate	Phenols	0.76	0.62	0.83	2.32	4.59E-37
2,6-Di-tert-butyl-4-ethylphenol	Benzene and substituted derivatives	0.76	0.67	0.82	1.05	3.59E-37
4-Hydroxy-3-(16-methylheptadecyl)-2H-pyran-2-one	Pyrans	0.76	0.66	0.83	0.89	1.92E-37
o-Ethyltoluene	Benzene and substituted derivatives	0.77	0.66	0.83	0.71	9.87E-39
6,10,14-Trimethyl-5,9,13-pentadecatrien-2-one	Prenol lipids	0.79	0.69	0.85	0.94	3.11E-42
Indole	Indoles and derivatives	0.79	0.68	0.86	0.59	2.54E-43
(9S,10E,12Z,15Z)-9-Hydroxy-10,12,15-octadecatrienoic acid	Lineolic acids and derivatives	0.80	0.68	0.86	1.38	3.29E-44
Perillyl acetate	Prenol lipids	0.82	0.67	0.89	1.33	8.99E-48
Nandrolone	Steroids and steroid derivatives	0.82	0.72	0.88	2.92	4.97E-49
12,13-EpOME	Fatty Acyls	0.83	0.73	0.88	1.07	4.46E-50
Dihydro-6-isopropyl-2,4-dimethyl-4H-1,3,5-dithiazine	Azacyclic compounds	0.83	0.74	0.88	1.54	1.04E-50
Alpha-dimorphecolic acid	Fatty Acyls	0.83	0.72	0.89	1.79	1.71E-51
Delta-12-Prostaglandin J2	Fatty Acyls	0.84	0.73	0.91	4.09	1.25E-53
13S-hydroxyoctadecadienoic acid	Fatty Acyls	0.85	0.77	0.89	1.27	2.01E-55
Quinceoxepine	Ethers	0.86	0.74	0.91	1.22	3.08E-57
4,8 Dimethylnonanoyl carnitine	Fatty Acyls	0.87	0.77	0.92	2.00	5.65E-60
Corchorifatty acid D	Fatty Acyls	0.87	0.77	0.91	2.02	5.65E-60
Arachidonic acid	Fatty Acyls	0.87	0.77	0.92	1.90	3.41E-61
(2'E,4'Z,7'Z,8E)-Colnelenic acid	Fatty Acyls	0.88	0.78	0.93	2.54	3.12E-64

Metabolites with absolute PCA loading over 0.5 were presented. Bootstrap 95% CIs of loadings was generated by drawing 1,000 bootstrap samples.

Table S2. Characteristics of human milk PC 2

Metabolites	Class	PCA loadings	Bootstrap CI of loadings		Contribution (%)	FDR
			Lower	Upper		
Beta-D-Glucopyranuronic acid	Organooxygen compounds	-0.69	-0.80	-0.36	0.76	2.89E-28
Dihydroneopterin phosphate	Pteridines and derivatives	-0.59	-0.72	-0.26	0.49	3.76E-19
1-(Malonylamino)cyclopropanecarboxylic acid	Carboxylic acids and derivatives	-0.57	-0.73	-0.30	0.72	9.40E-18
3-Methylguanine	Imidazopyrimidines	-0.54	-0.70	-0.27	0.52	7.39E-16
Uridine	Pyrimidine nucleosides	-0.53	-0.67	-0.21	0.41	2.64E-15
Uracil	Diazines	-0.53	-0.67	-0.22	0.41	2.98E-15
SM(d18:1/20:0)	Sphingolipids	0.50	0.29	0.64	0.48	1.91E-13
PC(24:0/14:1(9Z))	Glycerophospholipids	0.50	0.19	0.66	0.67	1.51E-13
Palmitoylethanolamide	Carboximidic acids and derivatives	0.51	0.31	0.63	0.41	1.18E-13
LysoPE(0:0/18:0)	Glycerophospholipids	0.51	0.30	0.67	0.60	6.01E-14
PE(P-18:1(11Z)/22:4(7Z,10Z,13Z,16Z))	Glycerophospholipids	0.52	0.33	0.67	0.54	2.49E-14
PC(20:3(8Z,11Z,14Z)/14:0)	Glycerophospholipids	0.52	0.24	0.65	0.62	1.06E-14
PE(P-18:1(9Z)/18:1(9Z))	Glycerophospholipids	0.53	0.38	0.72	0.48	6.71E-15
PE(P-18:1(11Z)/18:3(6Z,9Z,12Z))	Glycerophospholipids	0.53	0.33	0.65	0.44	6.24E-15
PS(18:0/22:6(4Z,7Z,10Z,13Z,16Z,19Z))	Glycerophospholipids	0.53	0.21	0.70	0.83	2.89E-15
PC(20:3(5Z,8Z,11Z)/20:0)	Glycerophospholipids	0.53	0.25	0.66	0.92	2.62E-15
6-Deoxohomodolichosterone	Steroids and steroid derivatives	0.54	0.30	0.66	0.60	2.08E-15
PC(20:4(8Z,11Z,14Z,17Z)/15:0)	Glycerophospholipids	0.54	0.38	0.68	0.69	9.97E-16
PE(16:0/20:4(5Z,8Z,11Z,14Z))	Glycerophospholipids	0.54	0.31	0.68	0.66	6.44E-16
PC(20:0/20:2(11Z,14Z))	Glycerophospholipids	0.54	0.24	0.68	1.09	6.27E-16
PI(20:3(5Z,8Z,11Z)/16:0)	Glycerophospholipids	0.55	0.26	0.68	0.89	4.55E-16
PC(16:0/16:0)	Glycerophospholipids	0.55	0.32	0.73	0.72	3.28E-16
N-Hexadecanoylpyrrolidine	Pyrrolidines	0.55	0.32	0.70	0.70	1.32E-16
Oleamide	Fatty Acyls	0.56	0.37	0.66	0.44	4.97E-17
Polyoxyethylene (600) monoricinoleate	Fatty Acyls	0.56	0.35	0.69	0.66	3.60E-17

PI(20:2(11Z,14Z)/18:2(9Z,12Z))	Glycerophospholipids	0.56	0.20	0.72	0.62	3.39E-17
PS(14:0/22:2(13Z,16Z))	Glycerophospholipids	0.57	0.21	0.70	0.69	2.04E-17
PC(18:3(6Z,9Z,12Z)/15:0)	Glycerophospholipids	0.57	0.33	0.72	0.61	1.27E-17
PE(18:1(9Z)/16:0)	Glycerophospholipids	0.58	0.36	0.82	0.73	2.63E-18
PC(22:2(13Z,16Z)/15:0)	Glycerophospholipids	0.58	0.23	0.71	0.66	1.64E-18
PI(22:5(4Z,7Z,10Z,13Z,16Z)/16:0)	Glycerophospholipids	0.59	0.23	0.77	0.45	4.19E-19
SM(d18:0/18:1(9Z))	Sphingolipids	0.59	0.40	0.69	0.66	3.57E-19
LysoPC(20:3(5Z,8Z,11Z))	Glycerophospholipids	0.59	0.36	0.73	0.79	1.92E-19
PC(20:0/14:0)	Glycerophospholipids	0.60	0.28	0.73	0.67	6.44E-20
SM(d18:1/24:1(15Z))	Sphingolipids	0.60	0.37	0.71	0.69	4.32E-20
PE(22:5(4Z,7Z,10Z,13Z,16Z)/16:0)	Glycerophospholipids	0.61	0.39	0.76	0.74	4.02E-21
PC(14:0/14:0)	Glycerophospholipids	0.61	0.29	0.73	0.92	4.02E-21
PE(22:6(4Z,7Z,10Z,13Z,16Z,19Z)/P-18:1(11Z))	Glycerophospholipids	0.62	0.31	0.71	0.51	3.15E-21
SM(d18:1/18:1(9Z))	Sphingolipids	0.62	0.41	0.72	0.68	1.81E-21
Cohibin A	Fatty Acyls	0.63	0.33	0.74	1.22	3.34E-22
PC(22:5(7Z,10Z,13Z,16Z,19Z)/15:0)	Glycerophospholipids	0.64	0.45	0.74	0.95	5.27E-23
SM(d18:0/14:0)	Sphingolipids	0.65	0.38	0.73	0.75	7.15E-24
PC(18:3(6Z,9Z,12Z)/18:0)	Glycerophospholipids	0.65	0.36	0.79	0.64	1.35E-24
PC(P-16:0/18:4(6Z,9Z,12Z,15Z))	Glycerophospholipids	0.66	0.45	0.75	0.77	3.58E-25
PE(P-18:1(9Z)/20:5(5Z,8Z,11Z,14Z,17Z))	Glycerophospholipids	0.66	0.41	0.77	0.66	2.37E-25
PC(20:1(11Z)/14:0)	Glycerophospholipids	0.67	0.31	0.78	0.81	1.41E-26
PE(P-18:0/22:4(7Z,10Z,13Z,16Z))	Glycerophospholipids	0.68	0.41	0.79	0.70	2.75E-27
PE(22:2(13Z,16Z)/18:1(11Z))	Glycerophospholipids	0.69	0.35	0.78	0.84	5.11E-28
PC(16:1(9Z)/16:1(9Z))	Glycerophospholipids	0.69	0.48	0.78	0.94	1.25E-28
SM(d18:1/16:0)	Sphingolipids	0.71	0.43	0.81	0.87	3.23E-30
PC(22:4(7Z,10Z,13Z,16Z)/14:0)	Glycerophospholipids	0.71	0.37	0.82	0.75	3.22E-30
Epomusenin A	Fatty Acyls	0.72	0.49	0.80	0.62	1.43E-31
PC(15:0/15:0)	Glycerophospholipids	0.72	0.42	0.82	1.15	9.96E-32
PC(20:2(11Z,14Z)/20:4(5Z,8Z,11Z,14Z))	Glycerophospholipids	0.72	0.41	0.81	0.97	9.96E-32

PE(P-18:0/18:2(9Z,12Z))	Glycerophospholipids	0.72	0.49	0.81	0.63	3.28E-32
SM(d17:1/24:1(15Z))	Sphingolipids	0.72	0.50	0.79	0.78	1.59E-32
Cohibin C	Fatty Acyls	0.73	0.38	0.85	0.86	3.72E-33
2-acetyl-1-alkyl-sn-glycero-3-phosphocholine	Glycerophospholipids	0.74	0.56	0.82	1.18	6.17E-35
PC(20:2(11Z,14Z)/15:0)	Glycerophospholipids	0.76	0.41	0.85	0.79	3.83E-37
PC(16:1(9Z)/15:0)	Glycerophospholipids	0.76	0.40	0.87	0.87	1.22E-37
PC(22:5(7Z,10Z,13Z,16Z,19Z)/16:1(9Z))	Glycerophospholipids	0.76	0.45	0.83	0.90	6.38E-38
PC(22:2(13Z,16Z)/16:1(9Z))	Glycerophospholipids	0.77	0.38	0.86	0.85	2.37E-38
PC(20:2(11Z,14Z)/14:0)	Glycerophospholipids	0.77	0.50	0.85	0.67	1.71E-38
PC(22:6(4Z,7Z,10Z,13Z,16Z,19Z)/15:0)	Glycerophospholipids	0.77	0.55	0.83	1.04	1.07E-39
PE(P-16:0/20:4(5Z,8Z,11Z,14Z))	Glycerophospholipids	0.78	0.53	0.87	0.74	8.74E-40
PC(22:5(4Z,7Z,10Z,13Z,16Z)/16:0)	Glycerophospholipids	0.78	0.46	0.86	0.73	5.86E-41
PC(18:1(11Z)/14:0)	Glycerophospholipids	0.78	0.45	0.86	0.89	4.17E-41
PC(18:2(9Z,12Z)/18:0)	Glycerophospholipids	0.79	0.44	0.87	0.74	2.61E-42
PC(20:1(11Z)/20:4(5Z,8Z,11Z,14Z))	Glycerophospholipids	0.80	0.45	0.88	1.03	2.13E-43
PC(22:4(7Z,10Z,13Z,16Z)/15:0)	Glycerophospholipids	0.81	0.52	0.89	1.05	4.53E-45
PE(20:4(5Z,8Z,11Z,14Z)/P-18:1(11Z))	Glycerophospholipids	0.81	0.53	0.86	0.66	1.80E-45
PC(20:3(8Z,11Z,14Z)/15:0)	Glycerophospholipids	0.84	0.56	0.88	1.07	7.18E-52
PC(22:5(4Z,7Z,10Z,13Z,16Z)/15:0)	Glycerophospholipids	0.85	0.58	0.90	1.18	4.20E-54
PE(20:0/18:4(6Z,9Z,12Z,15Z))	Glycerophospholipids	0.85	0.65	0.89	0.92	8.97E-55
Montecristin	Fatty Acyls	0.87	0.66	0.90	1.08	5.11E-59
PE(16:0/18:2(9Z,12Z))	Glycerophospholipids	0.87	0.65	0.90	1.02	1.20E-59

Metabolites with a absolute PCA loading over 0.5 were presented. Bootstrap 95% CIs of loadings was generated by drawing 1,000 bootstrap samples.

Table S3. Details of PC 1 results

Metabolites	Class	PCA loadings	Bootstrap CI of loadings		Contribution (%)	FDR
			Lower	Upper		

3,3,5-triiodo-L-thyronine-beta-D-glucuronoside	Carboxylic acids and derivatives	0.00	-0.15	0.18	0.00	9.80E-01
Cellobiose	Organooxygen compounds	-0.04	-0.17	0.09	0.00	6.17E-01
Adenine	Imidazopyrimidines	-0.56	-0.66	-0.40	0.42	3.88E-17
1-Amino-propan-2-ol	Organonitrogen compounds	0.14	0.00	0.27	0.02	7.08E-02
1-Pyrroline	Pyrrolines	-0.44	-0.56	-0.26	0.15	2.63E-10
Rheidin A	Anthracenes	-0.34	-0.49	-0.20	0.12	1.83E-06
1-Kestose	Organooxygen compounds	0.07	-0.10	0.23	0.00	4.22E-01
Niacinamide	Pyridines and derivatives	0.56	0.40	0.68	0.38	2.51E-17
5-Methylcytidine	Pyrimidine nucleosides	-0.24	-0.42	-0.06	0.03	1.14E-03
Yuccaol C	2-arylbenzofuran flavonoids	0.09	-0.07	0.24	0.01	2.56E-01
Adenosine	Purine nucleosides	-0.63	-0.74	-0.50	0.63	8.81E-23
1-Methylinosine	Purine nucleosides	-0.33	-0.49	-0.13	0.08	4.37E-06
Choline	Organonitrogen compounds	-0.17	-0.31	-0.01	0.01	2.56E-02
D-Proline	Carboxylic acids and derivatives	0.06	-0.13	0.26	0.00	4.38E-01
1H-Pyrrole-2-carboxaldehyde	Organooxygen compounds	0.38	0.24	0.50	0.05	1.36E-07
4-Aminophenol	Benzene and substituted derivatives	-0.27	-0.40	-0.11	0.04	3.07E-04
Pyrrolidonecarboxylic acid	Carboxylic acids and derivatives	-0.62	-0.75	-0.45	0.26	6.72E-22
Uracil	Diazines	0.04	-0.19	0.27	0.00	6.58E-01

5-Aminopentanoic acid	Carboxylic acids and derivatives	0.06	-0.10	0.20	0.00	4.79E-01
5,12-dihydroxy-6,8,10,14,17-eicosapentaenoic acid	NA	0.69	0.56	0.77	2.04	7.63E-29
Kynurenic acid	Quinolines and derivatives	-0.06	-0.20	0.09	0.00	4.83E-01
Cytidine monophosphate	Pyrimidine nucleotides	-0.45	-0.59	-0.27	0.23	1.23E-10
N2,N2-Dimethylguanosine	Purine nucleosides	-0.36	-0.49	-0.20	0.08	4.72E-07
Gonyautoxin VI	Saxitoxins, gonyautoxins, and derivatives	-0.09	-0.26	0.08	0.01	2.81E-01
4-(3,4-Dihydroxyphenyl)-2-hydroxy-1H-phenalen-1-one	Naphthalenes	0.10	-0.03	0.23	0.00	2.02E-01
Guanine	Imidazopyrimidines	-0.38	-0.52	-0.20	0.17	1.38E-07
Nicotinamide ribotide	NA	0.46	0.28	0.59	0.39	4.81E-11
Prolyl-Aspartate	Carboxylic acids and derivatives	0.19	0.00	0.35	0.03	1.07E-02
1-Methylnicotinamide	Pyridines and derivatives	0.23	0.07	0.39	0.03	1.76E-03
Pipecolic acid	Carboxylic acids and derivatives	0.39	0.24	0.51	0.12	4.27E-08
N-Ornithyl-L-taurine	Carboxylic acids and derivatives	0.05	-0.14	0.20	0.00	5.07E-01
D-Maltose	Organooxygen compounds	-0.51	-0.64	-0.31	0.18	6.67E-14
2-O-(6-Phospho-alpha-mannosyl)-D-glycerate	Organooxygen compounds	0.04	-0.11	0.17	0.00	6.54E-01
Taurine	Organic sulfonic acids and derivatives	0.09	-0.07	0.23	0.01	2.73E-01

2-O-Galloylsucrose	Benzene and substituted derivatives	-0.49	-0.60	-0.34	0.45	8.74E-13
L-Asparagine	Carboxylic acids and derivatives	-0.23	-0.37	-0.06	0.06	1.68E-03
Pyrrolidine	Pyrrolidines	-0.25	-0.39	-0.11	0.09	7.73E-04
Protoanemonin	Dihydrofurans	-0.01	-0.20	0.17	0.00	9.21E-01
Xanthotoxol glucoside	Coumarins and derivatives	-0.21	-0.33	-0.07	0.04	5.75E-03
L-Threonine	Carboxylic acids and derivatives	-0.30	-0.42	-0.13	0.07	4.79E-05
1-Methyladenosine	Purine nucleosides	-0.17	-0.35	0.03	0.02	2.55E-02
Propionylcarnitine	Fatty Acyls	0.23	0.04	0.39	0.04	1.93E-03
Phenylacetaldehyde	Benzene and substituted derivatives	0.13	-0.08	0.29	0.02	9.43E-02
N4-Acetylcytidine	Pyrimidine nucleosides	-0.27	-0.41	-0.12	0.12	3.00E-04
2,5-Dihydro-2,4-dimethyloxazole	Azolines	0.05	-0.11	0.20	0.01	5.21E-01
3-Methylhistidine	Carboxylic acids and derivatives	0.06	-0.12	0.24	0.01	4.57E-01
Sphinganine	Organonitrogen compounds	-0.11	-0.25	0.02	0.05	1.54E-01
Chondroitin	Organooxygen compounds	-0.19	-0.37	0.01	0.02	1.35E-02
2-Methylfuran	Heteroaromatic compounds	0.09	-0.08	0.22	0.05	2.37E-01
trans-3,3',4',5,5',7-Hexahydroxyflavanone	NA	0.03	-0.15	0.18	0.00	7.64E-01
Acetylhydrazine	Carboxylic acids and derivatives	-0.44	-0.59	-0.23	0.11	2.52E-10
Turanose	Fatty Acyls	-0.16	-0.31	0.02	0.02	3.82E-02

L-Palmitoylcarnitine	Fatty Acyls	0.33	0.12	0.51	0.27	5.41E-06
Lacto-N-fucopentaose III	Organooxygen compounds	-0.08	-0.25	0.11	0.01	3.43E-01
3-Acetamidobutanal	Organooxygen compounds	0.13	-0.01	0.27	0.01	9.51E-02
4-Aminohippuric acid	Benzene and substituted derivatives	-0.19	-0.38	0.01	0.03	1.11E-02
L-Phenylalanine	Carboxylic acids and derivatives	-0.07	-0.22	0.13	0.00	3.78E-01
Methyl 2-furoate	Furans	0.01	-0.17	0.17	0.00	8.91E-01
N-Hexadecanoylpyrrolidine	Pyrrolidines	0.36	0.10	0.54	0.18	5.17E-07
1-Methylhypoxanthine	NA	-0.31	-0.47	-0.11	0.07	2.36E-05
erythro-Isoleucine	NA	0.10	-0.06	0.26	0.01	2.19E-01
L-Acetylcarnitine	Fatty Acyls	-0.01	-0.18	0.15	0.00	8.69E-01
Trigonelline		0.20	0.06	0.34	0.08	6.81E-03
D-Serine	Carboxylic acids and derivatives	-0.33	-0.49	-0.13	0.09	5.90E-06
1-Methylguanosine	Purine nucleosides	-0.35	-0.48	-0.18	0.08	1.65E-06
Uridine	Pyrimidine nucleosides	0.07	-0.14	0.29	0.00	3.56E-01
2,5-Dimethyloxazole	Azoles	0.15	-0.05	0.31	0.03	4.96E-02
Adenosine 2'-phosphate	Organooxygen compounds	-0.15	-0.34	0.08	0.05	4.94E-02
Cytidine	Pyrimidine nucleosides	0.51	0.32	0.65	0.30	6.67E-14
5'-Methylthioadenosine	5'-deoxyribonucleosides	-0.57	-0.68	-0.49	0.39	9.50E-18
L-Lysine	Carboxylic acids and derivatives	0.46	0.32	0.57	0.22	4.87E-11
Piperidine	Piperidines	-0.01	-0.17	0.16	0.00	8.91E-01
3-Methylguanine	Imidazopyrimidines	-0.51	-0.66	-0.28	0.28	4.43E-14

Thiamine monophosphate	Diazines	0.29	0.12	0.45	0.08	9.82E-05
Sorbose 1-phosphate	Organooxygen compounds	-0.20	-0.37	-0.02	0.02	7.75E-03
Trimethylamine N-oxide	Organonitrogen compounds	0.19	0.03	0.33	0.04	1.35E-02
D-Glutamine	Carboxylic acids and derivatives	-0.73	-0.81	-0.58	0.76	3.39E-33
2-Butenal	NA	0.14	-0.06	0.31	0.03	7.71E-02
Styrene	Benzene and substituted derivatives	0.74	0.61	0.83	0.50	3.09E-34
Anserine	Peptidomimetics	-0.40	-0.49	-0.28	0.24	2.64E-08
Oleamide	Fatty Acyls	0.29	0.04	0.50	0.07	7.90E-05
Solasodine	Steroids and steroid derivatives	-0.08	-0.23	0.08	0.03	3.02E-01
Dihydroneopterin phosphate	Pteridines and derivatives	-0.39	-0.56	-0.15	0.13	4.24E-08
Diethanolamine	Organonitrogen compounds	0.15	-0.01	0.28	0.02	5.08E-02
2-Fucosyllactose	Organooxygen compounds	-0.08	-0.28	0.10	0.01	3.06E-01
Nicotine	Pyridines and derivatives	-0.08	-0.21	0.08	0.01	3.41E-01
Spinacetin 3-gentiobioside	Flavonoids	0.04	-0.14	0.19	0.01	6.38E-01
Citrulline	Carboxylic acids and derivatives	-0.63	-0.73	-0.46	0.32	1.73E-22
4-Hydroxybenzaldehyde	Organooxygen compounds	-0.01	-0.18	0.15	0.00	8.90E-01
Prolyl-Arginine	Carboxylic acids and derivatives	0.50	0.37	0.61	0.79	3.02E-13
Pantothenic acid	Alcohols and polyols	0.02	-0.12	0.14	0.00	8.04E-01

L-Valine	Carboxylic acids and derivatives	-0.25	-0.41	-0.06	0.04	7.24E-04
3-Amino-2-piperidone	Carboxylic acids and derivatives	0.16	-0.02	0.30	0.02	3.54E-02
Uralenneoside	Benzene and substituted derivatives	0.04	-0.12	0.18	0.00	6.15E-01
Lactaldehyde	Organooxygen compounds	-0.38	-0.54	-0.28	0.18	8.76E-08
Mesalazine	Benzene and substituted derivatives	-0.04	-0.21	0.13	0.00	6.10E-01
Muscomin	Homoisoflavonoids	-0.22	-0.37	-0.04	0.03	3.30E-03
Urocanic acid	Azoles	-0.04	-0.19	0.10	0.00	5.90E-01
Dimethyl dialkyl ammonium chloride	Quaternary ammonium salts	0.23	0.06	0.38	0.26	2.18E-03
NAD	(5'->5')-dinucleotides	-0.27	-0.44	-0.09	0.12	2.13E-04
Sialyl-Lewis X	Organooxygen compounds	-0.30	-0.46	-0.13	0.13	3.68E-05
N-Acetylhistidine	Carboxylic acids and derivatives	0.34	0.18	0.47	0.08	2.47E-06
3alpha,4,5,7alpha-Tetrahydro-5-hydroxy-1H-isoindole-1,3(2H)-dione	Isoindoles and derivatives	-0.20	-0.38	0.01	0.04	7.95E-03
2-Acetyl-3,6-dimethylpyrazine	Carbonyl compounds	-0.01	-0.16	0.14	0.00	8.91E-01
Homo-L-arginine	Carboxylic acids and derivatives	-0.46	-0.57	-0.32	0.25	2.45E-11
3,4',5,6-Tetrahydroxy-3',7-dimethoxyflavone 3-glucuronide	Flavonoids	-0.39	-0.51	-0.27	0.24	2.91E-08
L-Arginine	Carboxylic acids and derivatives	-0.05	-0.18	0.09	0.00	5.07E-01

Ornithine	Carboxylic acids and derivatives	0.28	0.13	0.41	0.08	1.50E-04
Biliverdin	Tetrapyrroles and derivatives	-0.83	-0.87	-0.74	1.82	4.46E-50
4,8 Dimethylnonanoyl carnitine	Fatty Acyls	0.87	0.77	0.92	2.00	5.65E-60
S-Adenosylmethionine	5'-deoxyribonucleosides	-0.14	-0.30	0.03	0.03	6.46E-02
1,2,3,4-Tetrahydro-b-carboline-1,3-dicarboxylic acid	Harmala alkaloids	-0.04	-0.19	0.09	0.00	5.95E-01
Spinatoside	Flavonoids	-0.49	-0.60	-0.36	0.40	1.37E-12
5-Methyl-2-furancarboxaldehyde	Carbonyl compounds	0.10	-0.09	0.25	0.06	2.22E-01
Tryptophan	NA	0.00	-0.19	0.21	0.00	9.51E-01
O-Acetylserine	Carboxylic acids and derivatives	-0.23	-0.39	-0.05	0.02	2.28E-03
(9S,10E,12Z,15Z)-9-Hydroxy-10,12,15-octadecatrienoic acid	Lineolic acids and derivatives	0.80	0.68	0.86	1.38	3.29E-44
FAPy-adenine	Diazines	-0.12	-0.29	0.03	0.02	1.22E-01
5-HEPE	Fatty Acyls	0.45	0.30	0.58	0.75	7.96E-11
7-Aminoclonazepam	Benzodiazepines	-0.18	-0.36	0.02	0.08	1.54E-02
Linoleamide	Fatty Acyls	0.04	-0.22	0.34	0.00	6.58E-01
Gerberinol	Coumarins and derivatives	0.02	-0.15	0.16	0.00	7.75E-01
Saccharopine	Carboxylic acids and derivatives	-0.32	-0.47	-0.15	0.18	7.81E-06
Pyroglutamic acid	Carboxylic acids and derivatives	-0.67	-0.78	-0.51	2.21	2.14E-26
LysoPE(18:1(9Z)/0:0)	Glycerophospholipids	-0.16	-0.37	0.06	0.04	3.88E-02
Octadecanamide	Fatty Acyls	0.08	-0.13	0.29	0.01	3.01E-01
DG(22:5(7Z,10Z,13Z,16Z,19Z)/16:1(9Z)/0:0)	Glycerolipids	0.69	0.56	0.79	0.79	6.18E-29

Trimethylaminoacetone	Organooxygen compounds	0.23	0.06	0.37	0.03	2.62E-03
Cholesterol	Steroids and steroid derivatives	0.54	0.40	0.66	0.58	1.35E-15
1-Salicylate glucuronide	Organooxygen compounds	-0.24	-0.41	-0.04	0.04	1.49E-03
Racemethionine	NA	-0.07	-0.25	0.08	0.00	3.52E-01
Allysine	Carboxylic acids and derivatives	-0.05	-0.20	0.10	0.00	5.66E-01
Pyro-L-glutaminyl-L-glutamine	Carboxylic acids and derivatives	-0.37	-0.51	-0.22	0.11	2.14E-07
UDP-N-acetyl-alpha-D-galactosamine	Pyrimidine nucleotides	-0.28	-0.42	-0.14	0.16	1.14E-04
4-Amino-2-methylenebutanoic acid	Carboxylic acids and derivatives	0.17	-0.02	0.32	0.03	3.10E-02
2-Furanmethanol	Heteroaromatic compounds	0.16	-0.01	0.30	0.04	3.43E-02
Ecgonine	Tropane alkaloids	0.12	-0.06	0.27	0.01	1.11E-01
5,6-Dihydroxyindole	Indoles and derivatives	-0.04	-0.19	0.10	0.00	6.02E-01
Valyl-Lysine	Carboxylic acids and derivatives	-0.36	-0.50	-0.18	0.07	4.69E-07
AICA-riboside	Imidazole ribonucleosides and ribonucleotides	-0.17	-0.34	0.00	0.05	2.57E-02
Beta-D-Glucopyranuronic acid	Organooxygen compounds	-0.42	-0.62	-0.12	0.17	3.42E-09
3-Oxohexadecanoyl-CoA	Fatty Acyls	-0.15	-0.35	0.05	0.04	4.38E-02
Homoarecoline		-0.11	-0.24	0.04	0.01	1.58E-01
Lacto-N-difucopentaose II	Organooxygen compounds	0.06	-0.13	0.21	0.00	4.56E-01

LysoPE(0:0/22:4(7Z,10Z,13Z,16Z))	Glycerophospholipids	-0.10	-0.32	0.14	0.01	2.18E-01
alpha-Zearalenol	Macrolides and analogues	-0.47	-0.61	-0.25	0.17	1.19E-11
L-Tyrosine	Carboxylic acids and derivatives	-0.06	-0.19	0.13	0.01	4.25E-01
LysoPE(20:1(11Z)/0:0)	Glycerophospholipids	0.43	0.26	0.58	0.29	5.99E-10
Thiamine	Diazines	-0.41	-0.52	-0.26	0.27	4.07E-09
LysoPE(16:0/0:0)	Glycerophospholipids	0.63	0.50	0.73	0.57	8.89E-23
Guanosine	Purine nucleosides	-0.37	-0.51	-0.21	0.19	2.24E-07
LysoPE(0:0/18:3(6Z,9Z,12Z))	Glycerophospholipids	-0.30	-0.45	-0.13	0.16	5.00E-05
beta-Lactose	Carbohydrates and carbohydrate conjugates	0.50	0.35	0.62	0.59	1.94E-13
3,4,5-Trimethoxycinnamic acid	Cinnamic acids and derivatives	-0.21	-0.36	-0.03	0.04	5.75E-03
Delta-12-Prostaglandin J2	Fatty Acyls	0.84	0.73	0.91	4.09	1.25E-53
N-Acetyl-L-alanine	Carboxylic acids and derivatives	0.16	0.01	0.30	0.03	3.18E-02
3-Hydroxyisovalerylcarnitine	Fatty Acyls	-0.12	-0.29	0.06	0.02	1.37E-01
Nicotinic acid	Pyridines and derivatives	0.13	-0.07	0.31	0.01	9.96E-02
4-Acetyl-3-methylpyridine	Carbonyl compounds	0.03	-0.13	0.20	0.00	7.22E-01
Phosphocreatine	Carboxylic acids and derivatives	0.23	0.08	0.36	0.30	2.05E-03
Palmitoylethanolamide	Carboximidic acids and derivatives	0.32	0.09	0.51	0.10	1.30E-05
3'-Sialyllactose	Organooxygen compounds	-0.03	-0.22	0.14	0.00	7.26E-01
2-Aminoacrylic acid	Carboxylic acids and derivatives	-0.28	-0.46	-0.07	0.22	1.30E-04
2,4-Dimethyloxazole	Azoles	0.26	0.12	0.38	0.02	4.69E-04

LysoPE(0:0/20:4(5Z,8Z,11Z,14Z))	Glycerophospholipids	-0.20	-0.41	0.03	0.06	6.81E-03
2-Azetidinecarboxylic acid	NA	-0.26	-0.41	-0.08	0.03	4.49E-04
Epsilon-caprolactam	Lactams	0.17	0.03	0.31	0.03	2.81E-02
Polyoxyethylene (600) monoricinoleate	Fatty Acyls	0.38	0.12	0.56	0.18	1.50E-07
1,3-Diisopropylbenzene	Benzene and substituted derivatives	0.37	0.23	0.49	0.11	3.49E-07
Triethanolamine	Amines	-0.07	-0.20	0.08	0.01	3.98E-01
Aminofructose 6-phosphate	Organooxygen compounds	-0.02	-0.17	0.14	0.00	8.18E-01
1-Methyl-1,3-cyclohexadiene	Olefins	0.63	0.49	0.73	0.32	1.07E-22
Epidermin	Organooxygen compounds	0.45	0.28	0.59	0.26	1.07E-10
Proline betaine	Carboxylic acids and derivatives	0.11	-0.03	0.28	0.03	1.69E-01
O-Phosphotyrosine	Carboxylic acids and derivatives	0.20	0.04	0.36	0.03	8.49E-03
N-Acetylputrescine	Carboximidic acids and derivatives	-0.29	-0.42	-0.11	0.08	8.83E-05
Dehydrophytosphingosine	Organonitrogen compounds	0.25	0.07	0.41	0.11	5.99E-04
13S-hydroxyoctadecadienoic acid	Fatty Acyls	0.85	0.77	0.89	1.27	2.01E-55
o-Xylene	Benzene and substituted derivatives	0.65	0.51	0.74	0.40	2.70E-24
Asparaginyl-Hydroxyproline	Carboxylic acids and derivatives	0.21	0.07	0.47	0.07	4.67E-03
Pivaloylcarnitine	Fatty Acyls	-0.12	-0.27	0.03	0.03	1.14E-01
Norvaline	Carboxylic acids and derivatives	-0.36	-0.49	-0.20	0.06	3.85E-07

SM(d18:1/16:0)	Sphingolipids	0.43	0.14	0.62	0.19	1.26E-09
(2R)-2-Hydroxy-2-methylbutanenitrile	Organooxygen compounds	0.05	-0.10	0.18	0.00	5.31E-01
Nandrolone	Steroids and steroid derivatives	0.82	0.72	0.88	2.92	4.97E-49
Arachidonic acid	Fatty Acyls	0.87	0.77	0.92	1.90	3.41E-61
2-trans-6-cis-Dodecadienal	Organooxygen compounds	0.35	0.21	0.47	0.12	1.51E-06
MG(22:2(13Z,16Z)/0:0/0:0)	Glycerolipids	-0.12	-0.33	0.07	0.02	1.11E-01
D-4'-Phosphopantothenate	Carboxylic acids and derivatives	-0.16	-0.30	-0.01	0.02	3.83E-02
Bilirubin	Organoheterocyclic compounds	-0.16	-0.37	0.08	0.05	3.18E-02
Vinylacetylglycine	Carboxylic acids and derivatives	0.26	0.08	0.41	0.03	5.88E-04
Alpha-dimorphecolic acid	Fatty Acyls	0.83	0.72	0.89	1.79	1.71E-51
(alpha-D-mannosyl)7-beta-D-mannosyl-diacetylchitobiosyl-L-asparagine, isoform A (protein)	Carboxylic acids and derivatives	-0.32	-0.45	-0.14	0.04	9.51E-06
Dihydro-6-isopropyl-2,4-dimethyl-4H-1,3,5-dithiazine	Azacyclic compounds	0.83	0.74	0.88	1.54	1.04E-50
Cohibin A	Fatty Acyls	0.26	0.02	0.43	0.13	4.98E-04
L-4-Hydroxyglutamate semialdehyde	Carboxylic acids and derivatives	0.00	-0.16	0.16	0.00	9.51E-01
N2-gamma-Glutamylglutamine	Carboxylic acids and derivatives	-0.75	-0.80	-0.67	1.08	1.02E-35
2-Methoxy-3,5-dimethylpyrimidine	Diazines	-0.22	-0.36	-0.06	0.03	3.40E-03

3-Hydroxy-4-butanolide	Organooxygen compounds	0.17	0.03	0.31	0.01	2.44E-02
N-Methylnicotinamide	Pyridines and derivatives	-0.67	-0.75	-0.56	0.45	8.65E-27
N6-Carbamoyl-L-threonyladenosine	Purine nucleosides	0.11	-0.07	0.28	0.01	1.69E-01
6-Chloro-N-(1-methylethyl)-1,3,5-triazine-2,4-diamine	Triazines	0.22	0.06	0.37	0.09	3.01E-03
L-alpha-Aspartyl-L-hydroxyproline	NA	0.16	0.04	0.27	0.04	3.64E-02
Glutaminylaspartic acid	Carboxylic acids and derivatives	0.10	-0.05	0.26	0.01	2.11E-01
N-Acetyldopamine	Phenols	0.14	-0.01	0.27	0.07	7.41E-02
1,4-Dideoxy-1,4-imino-D-ribitol	Pyrrolidines	-0.26	-0.38	-0.12	0.02	4.20E-04
Butyrylcarnitine	Fatty Acyls	-0.21	-0.36	-0.04	0.02	5.40E-03
Muricatacin		0.43	0.30	0.54	0.21	1.29E-09
Sonchuionoside C	Prenol lipids	-0.69	-0.80	-0.57	1.02	3.81E-28
Lactosylceramide (d18:1/16:0)	Sphingolipids	0.16	-0.04	0.35	0.04	3.50E-02
N-a-Acetyl-L-arginine	Carboxylic acids and derivatives	0.04	-0.17	0.23	0.00	6.54E-01
Avocadyne 4-acetate	Fatty Acyls	0.25	0.07	0.40	0.09	7.19E-04
4-(4-Methyl-3-pentenyl)-3-cyclohexene-1-carboxaldehyde	Prenol lipids	0.05	-0.11	0.20	0.00	5.86E-01
4-Hydroxymandelonitrile	NA	-0.21	-0.39	-0.01	0.04	5.93E-03
L-Aspartic acid	Carboxylic acids and derivatives	-0.15	-0.32	0.03	0.09	4.31E-02
LysoPE(0:0/22:1(13Z))	Glycerophospholipids	0.38	0.24	0.50	0.38	1.28E-07
Quinceoxepine	Ethers	0.86	0.74	0.91	1.22	3.08E-57
1-(beta-D-Ribofuranosyl)-1,4-dihydronicotinamide	NA	-0.12	-0.27	0.03	0.01	1.19E-01
LysoPE(0:0/18:0)	Glycerophospholipids	0.33	0.09	0.52	0.15	6.19E-06

N1-Methyl-4-pyridone-3-carboxamide	Pyridines and derivatives	0.02	-0.16	0.19	0.00	8.23E-01
Imidazole-4-acetaldehyde	Azoles	0.06	-0.10	0.24	0.00	4.50E-01
Capsiate	Phenols	0.76	0.62	0.83	2.32	4.59E-37
3-Dehydroxycarnitine	Fatty Acyls	-0.10	-0.25	0.04	0.01	1.92E-01
LysoPE(22:5(4Z,7Z,10Z,13Z,16Z)/0:0)	Glycerophospholipids	-0.14	-0.35	0.09	0.03	6.39E-02
Corchorifatty acid D	Fatty Acyls	0.87	0.77	0.91	2.02	5.65E-60
SM(d18:1/24:1(15Z))	Sphingolipids	0.26	-0.01	0.46	0.08	3.71E-04
HDMBOA-Glc	Organooxygen compounds	-0.25	-0.37	-0.12	0.04	7.81E-04
Dieporeticenin	Fatty Acyls	0.19	0.01	0.43	0.03	1.47E-02
LysoPE(18:2(9Z,12Z)/0:0)	Glycerophospholipids	-0.43	-0.60	-0.22	0.29	1.04E-09
Acrylamide	Carboximidic acids and derivatives	-0.08	-0.27	0.10	0.01	3.04E-01
N-Acetylneuraminic acid	Organooxygen compounds	-0.21	-0.36	-0.04	0.02	4.74E-03
Hydroxyisocaproic acid	Fatty Acyls	0.17	-0.07	0.36	0.01	2.89E-02
Dihydrouracil	Diazines	-0.52	-0.65	-0.32	0.15	1.30E-14
PC(16:0/16:0)	Glycerophospholipids	0.48	0.27	0.68	0.33	6.96E-12
Indole	Indoles and derivatives	0.79	0.68	0.86	0.59	2.54E-43
Formiminoglutamic acid	Carboxylic acids and derivatives	0.21	0.06	0.35	0.02	4.46E-03
PC(20:0/14:0)	Glycerophospholipids	0.48	0.24	0.63	0.26	3.41E-12
PC(18:1(11Z)/14:0)	Glycerophospholipids	0.30	-0.01	0.53	0.08	5.15E-05
LysoPE(22:6(4Z,7Z,10Z,13Z,16Z,19Z)/0:0)	Glycerophospholipids	-0.14	-0.36	0.10	0.03	7.66E-02
Hypoxanthine	Imidazopyrimidines	0.24	0.10	0.34	0.21	1.59E-03
LysoPE(16:1(9Z)/0:0)	Glycerophospholipids	0.03	-0.17	0.25	0.00	7.09E-01
Glucosamine 6-phosphate	Organooxygen compounds	-0.09	-0.25	0.07	0.01	2.56E-01

2-Methylpiperidine	Piperidines	-0.13	-0.23	0.01	0.02	8.50E-02
2-O-(5,8,11,14,17-Eicosapentaenoyl)-1-O-hexadecylglycero-3-phosphocholine	Glycerophospholipids	0.42	0.26	0.56	0.30	1.66E-09
PE(16:0/20:4(5Z,8Z,11Z,14Z))	Glycerophospholipids	-0.56	-0.70	-0.37	0.43	3.66E-17
10-Nitrolinoleic acid	Fatty Acyls	0.26	0.13	0.37	0.12	5.33E-04
PE(22:5(4Z,7Z,10Z,13Z,16Z)/16:0)	Glycerophospholipids	-0.55	-0.72	-0.34	0.36	2.40E-16
4-Hydroxy-L-glutamic acid	Organic acids and derivatives	-0.87	-0.91	-0.81	1.56	3.76E-61
Pipericine	Fatty Acyls	-0.01	-0.14	0.12	0.00	8.68E-01
PE(16:0/18:2(9Z,12Z))	Glycerophospholipids	-0.05	-0.35	0.23	0.00	5.22E-01
Montecristin	Fatty Acyls	-0.04	-0.34	0.24	0.00	6.18E-01
(4-Hydroxybenzoyl)choline	Carbonyl compounds	0.03	-0.12	0.15	0.00	7.62E-01
Deoxyeritadenine	Imidazopyrimidines	0.20	0.02	0.34	0.02	9.43E-03
PC(18:3(6Z,9Z,12Z)/18:0)	Glycerophospholipids	-0.42	-0.63	-0.17	0.16	3.25E-09
Nephritogenoside	Fatty Acyls	0.46	0.26	0.59	0.25	3.37E-11
o-Ethyltoluene	Benzene and substituted derivatives	0.77	0.66	0.83	0.71	9.87E-39
PE(P-18:1(11Z)/18:3(6Z,9Z,12Z))	Glycerophospholipids	0.10	-0.17	0.37	0.01	1.92E-01
beta-Alanine	Carboxylic acids and derivatives	-0.21	-0.35	-0.03	0.02	4.28E-03
PC(22:2(13Z,16Z)/P-18:0)	Glycerophospholipids	0.57	0.41	0.67	0.41	5.18E-18
DG(18:4(6Z,9Z,12Z,15Z)/16:1(9Z)/0:0)	Glycerolipids	0.29	0.11	0.45	0.09	6.31E-05
LysoPC(P-18:1(9Z))	Glycerophospholipids	0.47	0.24	0.66	0.45	1.28E-11
MG(0:0/22:4(7Z,10Z,13Z,16Z)/0:0)	Glycerolipids	-0.69	-0.78	-0.56	0.85	7.52E-29
SM(d16:1/24:1(15Z))	Sphingolipids	0.01	-0.20	0.23	0.00	8.92E-01
4-Aminobutyraldehyde	Organooxygen compounds	-0.29	-0.43	-0.12	0.04	8.15E-05

1-(Malonylamino)cyclopropanecarboxylic acid	Carboxylic acids and derivatives	-0.58	-0.73	-0.35	0.46	8.86E-19
Quercetin	Flavonoids	-0.31	-0.49	-0.09	0.22	2.09E-05
N-Acetyl-a-neuraminic acid	Organooxygen compounds	-0.14	-0.28	0.02	0.01	7.89E-02
Ethyl trans-p-methoxycinnamate	Cinnamic acids and derivatives	0.63	0.50	0.71	2.06	2.53E-22
4-Oxo-2-nonenal	Organooxygen compounds	0.01	-0.18	0.18	0.00	9.34E-01
3-Methyl sulfolene	Dihydrothiophenes	-0.15	-0.32	0.07	0.02	5.88E-02
PC(18:2(9Z,12Z)/18:0)	Glycerophospholipids	0.26	-0.06	0.50	0.05	4.48E-04
SM(d17:1/24:0)	Sphingolipids	-0.05	-0.22	0.12	0.00	5.04E-01
L-Octanoylcarnitine	Fatty Acyls	-0.02	-0.21	0.19	0.00	8.25E-01
gamma-Glutamylglutamic acid	Carboxylic acids and derivatives	-0.32	-0.44	-0.17	0.17	1.24E-05
Ipomeatetrahydrofuran	Prenol lipids	0.32	0.15	0.45	0.09	1.39E-05
LysoPE(20:5(5Z,8Z,11Z,14Z,17Z)/0:0)	Glycerophospholipids	-0.50	-0.63	-0.32	0.40	3.73E-13
Prolyl-Glutamine	Carboxylic acids and derivatives	0.06	-0.12	0.25	0.00	4.50E-01
PC(22:5(7Z,10Z,13Z,16Z,19Z)/15:0)	Glycerophospholipids	-0.07	-0.34	0.19	0.01	3.74E-01
PC(20:2(11Z,14Z)/14:0)	Glycerophospholipids	-0.01	-0.30	0.29	0.00	8.69E-01
PC(20:1(11Z)/14:0)	Glycerophospholipids	0.22	-0.06	0.44	0.05	4.09E-03
(R)-2-Hydroxysterculic acid	Fatty Acyls	0.38	0.27	0.56	0.20	1.03E-07
Fagomine	Piperidines	-0.10	-0.27	0.07	0.01	1.86E-01
Isoleucyl-Alanine	Carboxylic acids and derivatives	-0.22	-0.38	-0.04	0.03	3.98E-03
Glutamyllysine	Carboxylic acids and derivatives	-0.08	-0.24	0.08	0.02	2.92E-01

PC(15:0/15:0)	Glycerophospholipids	0.39	0.11	0.58	0.21	2.87E-08
MG(0:0/18:4(6Z,9Z,12Z,15Z)/0:0)	Glycerolipids	0.71	0.59	0.79	1.05	1.32E-30
N2-Maltulosylarginine	Saccharolipids	-0.18	-0.34	-0.03	0.12	1.57E-02
PC(22:2(13Z,16Z)/16:1(9Z))	Glycerophospholipids	0.33	0.03	0.57	0.10	3.69E-06
SM(d18:1/20:0)	Sphingolipids	0.05	-0.17	0.24	0.00	5.70E-01
Kojibiose	Fatty Acyls	0.12	-0.07	0.29	0.02	1.14E-01
Cytokinin B	Imidazopyrimidines	-0.11	-0.24	0.09	0.01	1.58E-01
DG(18:4(6Z,9Z,12Z,15Z)/14:1(9Z)/0:0)	Glycerolipids	0.14	-0.05	0.31	0.02	7.53E-02
PC(20:2(11Z,14Z)/20:4(5Z,8Z,11Z,14Z))	Glycerophospholipids	0.35	0.06	0.58	0.14	1.49E-06
SM(d17:1/24:1(15Z))	Sphingolipids	0.21	-0.08	0.44	0.04	4.46E-03
SM(d18:0/18:1(9Z))	Sphingolipids	0.09	-0.16	0.32	0.01	2.36E-01
5beta-Cholestane-3alpha,7alpha,24,26-tetrol	NA	-0.56	-0.68	-0.38	0.43	6.42E-17
PC(22:5(4Z,7Z,10Z,13Z,16Z)/16:0)	Glycerophospholipids	-0.18	-0.45	0.13	0.02	1.78E-02
PC(22:5(4Z,7Z,10Z,13Z,16Z)/P-18:0)	Glycerophospholipids	0.22	-0.01	0.42	0.09	3.96E-03
PC(24:0/14:1(9Z))	Glycerophospholipids	0.44	0.20	0.60	0.31	2.67E-10
LysoPC(16:0)	Glycerophospholipids	0.69	0.57	0.77	0.73	4.02E-28
Sialyllacto-N-tetraose b	Organooxygen compounds	0.00	-0.19	0.17	0.00	9.96E-01
SM(d18:1/14:0)	Sphingolipids	-0.14	-0.34	0.09	0.03	6.46E-02
PI(22:5(4Z,7Z,10Z,13Z,16Z)/16:0)	Glycerophospholipids	-0.23	-0.44	0.00	0.04	1.76E-03
Lactapiperanol D	Prenol lipids	0.72	0.60	0.79	1.09	1.16E-31
L-Gulonolactone	Lactones	0.16	0.00	0.30	0.01	3.42E-02
2,6-Di-tert-butyl-4-ethylphenol	Benzene and substituted derivatives	0.76	0.67	0.82	1.05	3.59E-37
PC(18:3(6Z,9Z,12Z)/18:1(11Z))	Glycerophospholipids	-0.45	-0.62	-0.22	0.32	1.04E-10
PC(22:4(7Z,10Z,13Z,16Z)/16:0)	Glycerophospholipids	-0.11	-0.35	0.13	0.02	1.73E-01
PC(22:5(4Z,7Z,10Z,13Z,16Z)/15:0)	Glycerophospholipids	0.21	-0.13	0.45	0.04	6.24E-03
PC(14:0/14:0)	Glycerophospholipids	0.30	0.05	0.48	0.13	5.32E-05

dADP	Purine nucleotides	-0.01	-0.18	0.14	0.00	9.40E-01
PC(20:1(11Z)/20:4(5Z,8Z,11Z,14Z))	Glycerophospholipids	0.33	0.01	0.57	0.11	6.61E-06
3-Benzyl-4-heptanone	Benzene and substituted derivatives	0.44	0.28	0.56	0.23	5.31E-10
Betonicine	Carboxylic acids and derivatives	-0.18	-0.31	-0.01	0.05	1.97E-02
Diepomuricanin A	NA	0.10	-0.11	0.36	0.01	1.94E-01
LysoPE(0:0/14:0)	Glycerophospholipids	0.50	0.35	0.60	0.62	4.33E-13
SM(d18:0/14:0)	Sphingolipids	0.12	-0.11	0.32	0.02	1.10E-01
PC(20:2(11Z,14Z)/15:0)	Glycerophospholipids	0.16	-0.13	0.40	0.02	3.55E-02
PC(22:2(13Z,16Z)/15:0)	Glycerophospholipids	0.15	-0.10	0.35	0.03	5.66E-02
2-(acetylamino)-1,5-anhydro-2-deoxy-3-O-b-D-galactopyranosyl-D-arabino-Hex-1-enitol	Organooxygen compounds	0.42	0.26	0.53	0.22	3.26E-09
PC(20:3(5Z,8Z,11Z)/20:0)	Glycerophospholipids	0.18	-0.05	0.37	0.06	1.82E-02
LysoPC(18:2(9Z,12Z))	Glycerophospholipids	0.15	-0.09	0.41	0.03	4.49E-02
Urothion	Pteridines and derivatives	0.32	0.20	0.42	0.42	1.05E-05
(2'E,4'Z,7'Z,8E)-Colnelenic acid	Fatty Acyls	0.88	0.78	0.93	2.54	3.12E-64
PC(20:0/20:2(11Z,14Z))	Glycerophospholipids	0.25	0.00	0.43	0.14	7.57E-04
Glycerylphosphorylethanolamine	Organic phosphoric acids and derivatives	-0.16	-0.29	-0.02	0.04	3.99E-02
6,10,14-Trimethyl-5,9,13-pentadecatrien-2-one	Prenol lipids	0.79	0.69	0.85	0.94	3.11E-42
trans-3,4-Dihydro-4,8-dihydroxy-3-methyl-1H-2-benzopyran-1-one	Benzopyrans	0.16	-0.03	0.33	0.02	3.42E-02
Pyridoxamine	Pyridines and derivatives	0.03	-0.11	0.18	0.00	7.20E-01
PC(22:4(7Z,10Z,13Z,16Z)/14:0)	Glycerophospholipids	-0.39	-0.61	-0.09	0.14	6.07E-08
PE(20:0/18:4(6Z,9Z,12Z,15Z))	Glycerophospholipids	0.14	-0.18	0.39	0.01	7.37E-02
SM(d18:1/18:1(9Z))	Sphingolipids	0.34	0.08	0.55	0.12	3.55E-06

Cohibin C	Fatty Acyls	0.43	0.14	0.63	0.18	6.63E-10
8-Hydroxy-2-octene-4,6-diynoic acid	Fatty Acyls	0.14	-0.07	0.28	0.09	7.79E-02
5-Hydroxy-L-tryptophan	Indoles and derivatives	-0.08	-0.22	0.06	0.00	2.96E-01
PC(20:4(8Z,11Z,14Z,17Z)/P-18:0)	Glycerophospholipids	0.28	0.07	0.46	0.09	1.34E-04
Phenylacetylglutamine	Carboxylic acids and derivatives	0.17	-0.01	0.33	0.02	2.70E-02
Isoeugenitol	Benzopyrans	-0.10	-0.25	0.06	0.01	1.85E-01
PC(16:1(9Z)/16:1(9Z))	Glycerophospholipids	-0.22	-0.45	0.03	0.06	4.09E-03
gamma-L-Glutamyl-gamma-L-glutamyl-L-methionine		-0.14	-0.28	0.01	0.03	7.91E-02
3-Oxoadipic acid	Keto acids and derivatives	0.42	0.26	0.52	0.07	3.83E-09
6-Deoxohomodolichosterone	Steroids and steroid derivatives	-0.47	-0.60	-0.30	0.28	1.19E-11
PC(18:1(9Z)/P-18:1(11Z))	Glycerophospholipids	0.33	0.08	0.54	0.10	3.69E-06
N-Salicyloylaspartic acid	Carboxylic acids and derivatives	-0.05	-0.23	0.12	0.00	5.15E-01
Vanilpyruvic acid	Benzene and substituted derivatives	0.10	-0.06	0.23	0.01	2.05E-01
PC(22:5(7Z,10Z,13Z,16Z,19Z)/16:1(9Z))	Glycerophospholipids	-0.10	-0.36	0.21	0.01	2.24E-01
LysoPC(22:6(4Z,7Z,10Z,13Z,16Z,19Z))	Glycerophospholipids	0.31	0.08	0.53	0.13	1.49E-05
Norsanguinarine	Quinolines and derivatives	0.24	0.05	0.39	0.05	1.25E-03
3-Methyl-5-pentyl-2-furanundecanoic acid	Fatty Acyls	0.69	0.58	0.78	0.52	7.10E-29
3-Aminopropionaldehyde	Organooxygen compounds	-0.43	-0.57	-0.23	0.12	1.36E-09
PC(22:5(7Z,10Z,13Z,16Z,19Z)/16:0)	Glycerophospholipids	-0.30	-0.54	-0.02	0.14	5.54E-05
PC(20:3(8Z,11Z,14Z)/14:0)	Glycerophospholipids	-0.13	-0.36	0.09	0.02	9.51E-02
Dihydromaleimide	NA	-0.71	-0.80	-0.55	0.46	3.33E-31

2,5-Dioxopentanoate	Keto acids and derivatives	-0.01	-0.14	0.14	0.00	9.34E-01
CS-S-Methylcysteine sulfoxide	NA	-0.24	-0.40	-0.09	0.21	1.03E-03
Oxonantenine	Aporphines	0.16	0.00	0.30	0.02	3.18E-02
DG(14:1(9Z)/18:3(6Z,9Z,12Z)/0:0)	Glycerolipids	0.42	0.23	0.56	0.17	3.25E-09
2-Acetylpyridine	Carbonyl compounds	-0.19	-0.33	-0.03	0.02	1.40E-02
2-Hydroxy-6,7-dimethoxybenzoxazole	Benzoxazoles	0.34	0.17	0.48	0.07	2.69E-06
Perillyl acetate	NA	0.82	0.67	0.89	1.33	8.99E-48
N-[(4E,8E)-1,3-dihydroxyoctadeca-4,8-dien-2-yl]hexadecanamide	Sphingolipids	0.14	-0.03	0.27	0.03	7.51E-02
Dihydro-3-(1-octenyl)-2,5-furandione	Oxolanes	0.68	0.54	0.78	0.66	6.19E-28
Artonin Q	Benzopyrans	-0.08	-0.29	0.15	0.01	3.18E-01
2-Aminomuconic acid semialdehyde	Carboxylic acids and derivatives	-0.09	-0.25	0.08	0.02	2.29E-01
DG(20:4(8Z,11Z,14Z,17Z)/14:0/0:0)	Glycerolipids	0.38	0.21	0.54	0.16	8.67E-08
Lacto-N-triose I	Organooxygen compounds	0.40	0.23	0.52	0.19	2.64E-08
PC(P-18:1(9Z)/14:1(9Z))	Glycerophospholipids	0.01	-0.17	0.17	0.00	8.91E-01
(2S,4S)-Pinnatanine	Carboxylic acids and derivatives	0.04	-0.17	0.24	0.00	5.97E-01
O-6-deoxy-a-L-galactopyranosyl-(1->2)-O-b-D-galactopyranosyl-(1->3)-2-(acetylamino)-1,5-anhydro-2-deoxy-D-arabino-Hex-1-enitol	Organooxygen compounds	0.42	0.18	0.58	0.20	1.65E-09
Persicachrome	Prenol lipids	-0.10	-0.33	0.15	0.01	2.05E-01
L-trans-5-Hydroxy-2-piperidinecarboxylic acid	Carboxylic acids and derivatives	-0.04	-0.19	0.12	0.00	6.17E-01
2-acetyl-1-alkyl-sn-glycero-3-phosphocholine	Glycerophospholipids	0.19	-0.10	0.44	0.05	1.10E-02

PC(16:1(9Z)/15:0)	Glycerophospholipids	0.40	0.11	0.61	0.15	1.12E-08
LysoPC(P-16:0)	Glycerophospholipids	0.67	0.52	0.78	0.61	2.14E-26
Phenol glucuronide	Organooxygen compounds	0.63	0.48	0.72	0.50	1.88E-22
PC(18:3(6Z,9Z,12Z)/15:0)	Glycerophospholipids	-0.64	-0.79	-0.44	0.47	8.69E-24
PI(20:2(11Z,14Z)/18:2(9Z,12Z))	Glycerophospholipids	0.44	0.20	0.60	0.23	3.39E-10
Glycine	Carboxylic acids and derivatives	-0.40	-0.53	-0.24	0.11	1.12E-08
DG(14:1(9Z)/18:2(9Z,12Z)/0:0)	Glycerolipids	0.38	0.22	0.51	0.15	1.02E-07
Epomusenin A	Fatty Acyls	0.31	0.05	0.51	0.07	2.41E-05
Troxerutin	Flavonoids	0.19	0.04	0.35	0.06	1.27E-02
LysoPE(20:2(11Z,14Z)/0:0)	Glycerophospholipids	0.25	0.06	0.43	0.12	7.80E-04
PE(P-18:1(11Z)/22:4(7Z,10Z,13Z,16Z))	Glycerophospholipids	0.04	-0.23	0.30	0.00	6.65E-01
PC(22:4(7Z,10Z,13Z,16Z)/15:0)	Glycerophospholipids	0.26	-0.03	0.48	0.07	3.71E-04
1-Methylpyrrole	Pyrroles	0.15	-0.02	0.29	0.02	5.56E-02
LysoPC(22:5(4Z,7Z,10Z,13Z,16Z))	Glycerophospholipids	0.31	0.10	0.49	0.24	2.36E-05
5-Hydroxyindoleacetic acid	Indoles and derivatives	0.32	0.19	0.44	0.09	7.65E-06
Seryltyrosine	Carboxylic acids and derivatives	-0.16	-0.30	-0.01	0.01	3.24E-02
5-Hydroxyisourate		-0.73	-0.81	-0.60	3.81	7.16E-34
Lacto-N-biose I	Organooxygen compounds	-0.55	-0.65	-0.40	0.24	2.91E-16
PE(P-18:1(11Z)/18:2(9Z,12Z))	Glycerophospholipids	-0.14	-0.41	0.10	0.03	7.08E-02
3-(3,4-Dihydroxy-5-methoxy)-2-propenoic acid	Cinnamic acids and derivatives	-0.03	-0.20	0.13	0.00	7.56E-01
4-Hydroxy-3-(16-methylheptadecyl)-2H-pyran-2-one	Pyrans	0.76	0.66	0.83	0.89	1.92E-37
LysoPC(20:3(5Z,8Z,11Z))	Glycerophospholipids	0.13	-0.12	0.36	0.02	8.12E-02

PI(20:3(5Z,8Z,11Z)/16:0)	Glycerophospholipids	-0.44	-0.60	-0.24	0.35	4.97E-10
1-(3,5-Dihydroxyphenyl)-2-pentadecanone	Phenols	0.24	0.09	0.39	0.11	1.30E-03
Glucocheirolin	Organooxygen compounds	-0.22	-0.41	-0.02	0.07	3.08E-03
PE(18:1(9Z)/16:0)	Glycerophospholipids	0.23	0.02	0.42	0.07	1.99E-03
5-Hydroxy-7-methoxy-2-tritriacontyl-4H-1-benzopyran-4-one	Benzopyrans	-0.56	-0.73	-0.37	0.53	7.30E-17
DG(14:1(9Z)/22:5(4Z,7Z,10Z,13Z,16Z)/0:0)	Glycerolipids	-0.07	-0.24	0.11	0.02	3.78E-01
12,13-EpOME	Fatty Acyls	0.83	0.73	0.88	1.07	4.46E-50
PE(20:4(8Z,11Z,14Z,17Z)/P-16:0)	Glycerophospholipids	0.34	0.20	0.61	0.14	3.29E-06
3,5-Dichloro-4-hydroxy-2-methoxy-6-methylbenzoic acid	Benzene and substituted derivatives	-0.05	-0.23	0.11	0.00	5.21E-01
Hydroxyphenylacetylglycine	Carboxylic acids and derivatives	-0.11	-0.22	0.02	0.00	1.58E-01
DG(22:4(7Z,10Z,13Z,16Z)/18:3(6Z,9Z,12Z)/0:0)	Glycerolipids	0.71	0.60	0.81	1.08	1.06E-30
PC(22:6(4Z,7Z,10Z,13Z,16Z,19Z)/15:0)	Glycerophospholipids	0.23	-0.08	0.46	0.05	2.61E-03
PS(14:0/22:2(13Z,16Z))	Glycerophospholipids	0.27	0.05	0.44	0.09	2.95E-04
PC(20:4(8Z,11Z,14Z,17Z)/15:0)	Glycerophospholipids	-0.02	-0.29	0.21	0.00	8.10E-01
Epiafzelechin 3-gallate		0.09	-0.05	0.25	0.02	2.40E-01
PE(18:1(11Z)/18:2(9Z,12Z))	Glycerophospholipids	-0.49	-0.78	-0.18	0.41	1.45E-12
PE(P-18:1(9Z)/16:1(9Z))	Glycerophospholipids	0.18	0.02	0.38	0.04	1.75E-02
Demethylated antipyrine	Azoles	0.30	0.13	0.44	0.10	4.21E-05
PE(P-18:1(11Z)/22:5(4Z,7Z,10Z,13Z,16Z))	Glycerophospholipids	0.10	-0.15	0.32	0.01	2.23E-01
3-Fucosyllactose	Organooxygen compounds	-0.47	-0.59	-0.32	0.32	2.13E-11
LysoPI(18:0/0:0)	Glycerophospholipids	-0.12	-0.26	0.03	0.01	1.12E-01
Isolinderanolide	Tetrahydrofurans	-0.44	-0.61	-0.21	0.28	2.67E-10

PE(P-18:1(9Z)/20:3(5Z,8Z,11Z))	Glycerophospholipids	0.37	0.21	0.60	0.18	2.75E-07
Marmesin rhamnoside	Coumarins and derivatives	0.08	-0.09	0.24	0.00	3.42E-01
1-Isothiocyanato-6-(methylthio)hexane	Isothiocyanates	-0.39	-0.52	-0.24	0.12	2.87E-08
PE(P-18:1(9Z)/20:5(5Z,8Z,11Z,14Z,17Z))	Glycerophospholipids	0.40	0.14	0.60	0.15	1.68E-08
PC(P-16:0/18:4(6Z,9Z,12Z,15Z))	Glycerophospholipids	0.32	0.05	0.52	0.11	1.33E-05
Obtusilactone A	Tetrahydrofurans	0.25	0.08	0.40	0.06	8.20E-04
PE(P-18:1(9Z)/18:1(9Z))	Glycerophospholipids	0.24	0.05	0.44	0.06	1.26E-03
PE(22:6(4Z,7Z,10Z,13Z,16Z,19Z)/P-18:1(11Z))	Glycerophospholipids	0.17	-0.09	0.42	0.02	2.84E-02
PE(20:4(5Z,8Z,11Z,14Z)/P-18:1(11Z))	Glycerophospholipids	0.16	-0.14	0.44	0.02	3.31E-02
Cohibin B	Fatty Acyls	0.33	0.16	0.51	0.09	6.37E-06
Dide-O-methyl-4-O-alpha-D-glucopyranosylsimmondsin	Organooxygen compounds	0.27	-0.01	0.48	0.06	2.35E-04
PC(P-18:1(9Z)/16:1(9Z))	Glycerophospholipids	0.42	0.34	0.49	0.62	3.16E-09
1-Isothiocyanato-6-(methylsulfinyl)hexane	Sulfoxides	0.04	-0.16	0.22	0.00	6.62E-01
PC(20:3(8Z,11Z,14Z)/15:0)	Glycerophospholipids	0.16	-0.14	0.40	0.02	3.94E-02
3-Isopropenylpentanedioic acid	Fatty Acyls	-0.21	-0.37	-0.05	0.02	4.39E-03
PE(P-16:0/20:4(5Z,8Z,11Z,14Z))	Glycerophospholipids	0.44	0.14	0.65	0.14	5.10E-10
Glaucarubin	Prenol lipids	-0.77	-0.82	-0.69	1.46	5.18E-39
PE(22:2(13Z,16Z)/18:1(11Z))	Glycerophospholipids	0.22	-0.06	0.44	0.05	2.87E-03
PE(P-18:0/18:2(9Z,12Z))	Glycerophospholipids	0.34	0.06	0.55	0.08	3.60E-06
Melleolide M	Prenol lipids	-0.75	-0.81	-0.65	1.49	2.07E-36
PE(P-18:0/22:4(7Z,10Z,13Z,16Z))	Glycerophospholipids	0.44	0.18	0.63	0.18	3.50E-10
Metiamide	Azoles	-0.30	-0.44	-0.11	0.09	3.71E-05
PS(18:0/22:6(4Z,7Z,10Z,13Z,16Z,19Z))	Glycerophospholipids	0.46	0.25	0.60	0.38	3.12E-11

Table S4. Details of PC2 results

Metabolites	Class	PCA loadings	Bootstrap CI of loadings		Contribution (%)	FDR
			Lower	Upper		
3,3,5-triiodo-L-thyronine-beta-D-glucuronoside	Carboxylic acids and derivatives	0.03	-0.15	0.23	0.00	7.18E-01
Cellobiose	Organooxygen compounds	-0.07	-0.21	0.10	0.01	4.14E-01
Adenine	Imidazopyrimidines	-0.31	-0.49	-0.07	0.20	3.27E-05
1-Amino-propan-2-ol	Organonitrogen compounds	0.05	-0.24	0.28	0.00	5.22E-01
1-Pyrroline	Pyrrolines	-0.35	-0.52	-0.15	0.16	8.60E-07
Rheidin A	Anthracenes	-0.12	-0.36	0.13	0.02	1.30E-01
1-Kestose	Organooxygen compounds	0.25	0.03	0.41	0.06	9.46E-04
Niacinamide	Pyridines and derivatives	-0.12	-0.29	0.09	0.03	1.40E-01
5-Methylcytidine	Pyrimidine nucleosides	-0.39	-0.67	-0.10	0.15	4.25E-08
Yuccaol C	2-arylbenzofuran flavonoids	0.27	0.05	0.42	0.08	3.67E-04
Adenosine	Purine nucleosides	0.07	-0.16	0.27	0.01	4.19E-01
1-Methylinosine	Purine nucleosides	-0.32	-0.49	-0.03	0.13	1.17E-05
Choline	Organonitrogen compounds	-0.17	-0.38	0.08	0.02	3.36E-02
D-Proline	Carboxylic acids and derivatives	0.14	-0.02	0.29	0.01	7.95E-02
1H-Pyrrole-2-carboxaldehyde	Organooxygen compounds	0.10	-0.11	0.25	0.01	2.24E-01

4-Aminophenol	Benzene and substituted derivatives	-0.18	-0.33	0.00	0.03	1.68E-02
Pyrrolidonecarboxylic acid	Carboxylic acids and derivatives	-0.27	-0.58	-0.01	0.08	3.65E-04
Uracil	Diazines	-0.53	-0.67	-0.22	0.41	2.98E-15
5-Aminopentanoic acid	Carboxylic acids and derivatives	0.12	-0.16	0.33	0.02	1.33E-01
5,12-dihydroxy-6,8,10,14,17-eicosapentaenoic acid	NA	-0.38	-0.56	-0.13	1.01	1.16E-07
Kynurenic acid	Quinolines and derivatives	-0.06	-0.21	0.15	0.01	4.62E-01
Cytidine monophosphate	Pyrimidine nucleotides	0.04	-0.36	0.27	0.00	6.57E-01
N2,N2-Dimethylguanosine	Purine nucleosides	-0.19	-0.38	0.13	0.04	1.31E-02
Gonyautoxin VI	Saxitoxins, gonyautoxins, and derivatives	-0.22	-0.47	0.00	0.07	4.67E-03
4-(3,4-Dihydroxyphenyl)-2-hydroxy-1H-phenalen-1-one	Naphthalenes	-0.07	-0.21	0.06	0.00	4.17E-01
Guanine	Imidazopyrimidines	-0.14	-0.38	0.15	0.04	7.20E-02
Nicotinamide ribotide	NA	0.18	-0.13	0.42	0.10	2.11E-02
Prolyl-Aspartate	Carboxylic acids and derivatives	0.27	-0.02	0.47	0.08	3.03E-04
1-Methylnicotinamide	Pyridines and derivatives	0.21	0.02	0.39	0.04	5.15E-03
Pipecolic acid	Carboxylic acids and derivatives	-0.25	-0.41	0.02	0.08	1.06E-03

N-Ornithyl-L-taurine	Carboxylic acids and derivatives	0.16	0.01	0.39	0.05	3.81E-02
D-Maltose	Organooxygen compounds	-0.38	-0.57	-0.07	0.16	1.09E-07
2-O-(6-Phospho-alpha-mannosyl)-D-glycerate	Organooxygen compounds	-0.09	-0.23	0.09	0.01	2.88E-01
Taurine	Organic sulfonic acids and derivatives	0.00	-0.22	0.20	0.00	9.88E-01
2-O-Galloylsucrose	Benzene and substituted derivatives	-0.14	-0.37	0.14	0.06	6.78E-02
L-Asparagine	Carboxylic acids and derivatives	-0.10	-0.29	0.10	0.02	2.36E-01
Pyrrolidine	Pyrrolidines	0.03	-0.14	0.19	0.00	7.08E-01
Protoanemonin	Dihydrofurans	0.06	-0.17	0.41	0.01	4.43E-01
Xanthotoxol glucoside	Coumarins and derivatives	0.02	-0.19	0.33	0.00	7.96E-01
L-Threonine	Carboxylic acids and derivatives	-0.37	-0.54	-0.12	0.18	2.52E-07
1-Methyladenosine	Purine nucleosides	-0.26	-0.45	0.04	0.09	4.04E-04
Propionylcarnitine	Fatty Acyls	0.36	0.12	0.61	0.16	6.44E-07
Phenylacetaldehyde	Benzene and substituted derivatives	0.04	-0.26	0.41	0.00	6.24E-01
N4-Acetylcytidine	Pyrimidine nucleosides	-0.13	-0.52	0.28	0.05	1.16E-01
2,5-Dihydro-2,4-dimethyloxazole	Azolines	0.10	-0.08	0.25	0.03	2.47E-01
3-Methylhistidine	Carboxylic acids and derivatives	-0.16	-0.36	0.04	0.08	4.63E-02

Sphinganine	Organonitrogen compounds	0.07	-0.11	0.21	0.03	4.19E-01
Chondroitin	Organooxygen compounds	-0.29	-0.47	-0.08	0.08	7.96E-05
2-Methylfuran	Heteroaromatic compounds	-0.06	-0.28	0.18	0.04	4.39E-01
trans-3,3',4',5,5',7-Hexahydroxyflavanone	NA	-0.02	-0.19	0.18	0.00	8.01E-01
Acetylhydrazine	Carboxylic acids and derivatives	-0.40	-0.60	-0.13	0.15	1.81E-08
Turanose	Fatty Acyls	-0.22	-0.43	-0.03	0.05	3.00E-03
L-Palmitoylcarnitine	Fatty Acyls	0.45	0.26	0.58	0.83	1.58E-10
Lacto-N-fucopentaose III	Organooxygen compounds	0.00	-0.31	0.22	0.00	9.90E-01
3-Acetamidobutanal	Organooxygen compounds	-0.08	-0.24	0.08	0.01	3.13E-01
4-Aminohippuric acid	Benzene and substituted derivatives	0.30	0.04	0.48	0.11	4.07E-05
L-Phenylalanine	Carboxylic acids and derivatives	0.05	-0.13	0.23	0.00	5.95E-01
Methyl 2-furoate	Furans	-0.02	-0.27	0.36	0.00	8.54E-01
N-Hexadecanoylpyrrolidine	Pyrrolidines	0.55	0.32	0.70	0.70	1.32E-16
1-Methylhypoxanthine	NA	-0.35	-0.51	-0.08	0.16	1.10E-06
erythro-Isoleucine	NA	0.06	-0.13	0.25	0.00	4.66E-01
L-Acetylcarnitine	Fatty Acyls	0.17	-0.06	0.55	0.02	2.43E-02
Trigonelline		-0.10	-0.30	0.11	0.03	2.24E-01
D-Serine	Carboxylic acids and derivatives	-0.28	-0.49	0.03	0.11	1.71E-04
1-Methylguanosine	Purine nucleosides	-0.03	-0.22	0.23	0.00	7.49E-01

Uridine	Pyrimidine nucleosides	-0.53	-0.67	-0.21	0.41	2.64E-15
2,5-Dimethyloxazole	Azoles	-0.08	-0.30	0.20	0.01	3.66E-01
Adenosine 2'-phosphate	Organooxygen compounds	0.42	-0.05	0.58	0.68	3.98E-09
Cytidine	Pyrimidine nucleosides	-0.37	-0.53	-0.11	0.27	1.79E-07
5'-Methylthioadenosine	5'-deoxyribonucleosides	0.02	-0.23	0.28	0.00	8.14E-01
L-Lysine	Carboxylic acids and derivatives	-0.21	-0.37	0.03	0.08	4.96E-03
Piperidine	Piperidines	-0.04	-0.19	0.13	0.00	5.99E-01
3-Methylguanine	Imidazopyrimidines	-0.54	-0.70	-0.27	0.52	7.39E-16
Thiamine monophosphate	Diazines	-0.02	-0.43	0.23	0.00	8.00E-01
Sorbose 1-phosphate	Organooxygen compounds	-0.30	-0.42	-0.15	0.07	4.09E-05
Trimethylamine N-oxide	Organonitrogen compounds	0.08	-0.10	0.24	0.01	3.72E-01
D-Glutamine	Carboxylic acids and derivatives	-0.21	-0.52	0.04	0.10	6.31E-03
2-Butenal	NA	-0.07	-0.29	0.22	0.01	4.06E-01
Styrene	Benzene and substituted derivatives	-0.15	-0.33	0.10	0.03	5.55E-02
Anserine	Peptidomimetics	-0.06	-0.23	0.10	0.01	4.39E-01
Oleamide	Fatty Acyls	0.56	0.37	0.66	0.44	4.97E-17
Solasodine	Steroids and steroid derivatives	0.17	-0.03	0.33	0.21	2.66E-02

Dihydroneopterin phosphate	Pteridines and derivatives	-0.59	-0.72	-0.26	0.49	3.76E-19
Diethanolamine	Organonitrogen compounds	-0.13	-0.35	0.04	0.02	9.21E-02
2-Fucosyllactose	Organooxygen compounds	-0.08	-0.32	0.26	0.02	3.64E-01
Nicotine	Pyridines and derivatives	-0.03	-0.16	0.11	0.00	6.88E-01
Spinacetin 3-gentiobioside	Flavonoids	-0.10	-0.30	0.20	0.06	2.44E-01
Citrulline	Carboxylic acids and derivatives	-0.32	-0.52	-0.03	0.13	1.68E-05
4-Hydroxybenzaldehyde	Organooxygen compounds	-0.02	-0.14	0.12	0.00	8.53E-01
Prolyl-Arginine	Carboxylic acids and derivatives	-0.18	-0.35	0.06	0.18	1.71E-02
Pantothenic acid	Alcohols and polyols	-0.02	-0.16	0.16	0.00	8.35E-01
L-Valine	Carboxylic acids and derivatives	-0.33	-0.55	-0.08	0.10	6.86E-06
3-Amino-2-piperidone	Carboxylic acids and derivatives	-0.11	-0.27	0.10	0.01	1.64E-01
Uralenneoside	Benzene and substituted derivatives	-0.08	-0.25	0.15	0.01	3.72E-01
Lactaldehyde	Organooxygen compounds	-0.01	-0.25	0.22	0.00	9.11E-01
Mesalazine	Benzene and substituted derivatives	-0.09	-0.23	0.06	0.01	2.67E-01
Muscomin	Homoisoflavonoids	-0.13	-0.36	0.09	0.02	1.18E-01

Urocanic acid	Azoles	0.12	-0.03	0.29	0.05	1.45E-01
Dimethyl dialkyl ammonium chloride	Quaternary ammonium salts	0.00	-0.20	0.22	0.00	9.88E-01
NAD	(5'->5')-dinucleotides	0.43	0.12	0.60	0.47	1.16E-09
Sialyl-Lewis X	Organooxygen compounds	-0.24	-0.51	0.06	0.13	1.42E-03
N-Acetylhistidine	Carboxylic acids and derivatives	0.20	-0.04	0.35	0.05	9.36E-03
3alpha,4,5,7alpha-Tetrahydro-5-hydroxy-1H-isoindole-1,3(2H)-dione	Isoindoles and derivatives	-0.20	-0.49	0.08	0.07	8.12E-03
2-Acetyl-3,6-dimethylpyrazine	Carbonyl compounds	0.06	-0.10	0.19	0.00	4.93E-01
Homo-L-arginine	Carboxylic acids and derivatives	-0.23	-0.42	0.05	0.10	2.00E-03
3,4',5,6-Tetrahydroxy-3',7-dimethoxyflavone 3-glucuronide	Flavonoids	-0.15	-0.40	0.13	0.06	5.75E-02
L-Arginine	Carboxylic acids and derivatives	-0.19	-0.39	0.09	0.05	1.55E-02
Ornithine	Carboxylic acids and derivatives	-0.06	-0.23	0.11	0.01	4.41E-01
Biliverdin	Tetrapyrroles and derivatives	0.13	-0.13	0.36	0.08	9.21E-02
4,8 Dimethylnonanoyl carnitine	Fatty Acyls	-0.29	-0.47	-0.04	0.36	1.10E-04
S-Adenosylmethionine	5'-deoxyribonucleosides	-0.10	-0.49	0.28	0.02	2.03E-01
1,2,3,4-Tetrahydro-b-carboline-1,3-dicarboxylic acid	Harmala alkaloids	-0.06	-0.21	0.11	0.00	4.90E-01
Spinatoside	Flavonoids	-0.14	-0.37	0.16	0.05	9.01E-02
5-Methyl-2-furancarboxaldehyde	Carbonyl compounds	-0.06	-0.27	0.20	0.04	4.62E-01
Tryptophan	NA	0.04	-0.13	0.21	0.00	6.78E-01

O-Acetylserine	Carboxylic acids and derivatives	-0.34	-0.63	-0.08	0.08	4.00E-06
(9S,10E,12Z,15Z)-9-Hydroxy-10,12,15-octadecatrienoic acid	Lineolic acids and derivatives	-0.30	-0.49	-0.05	0.33	3.85E-05
FAPy-adenine	Diazines	-0.20	-0.43	-0.06	0.09	8.55E-03
5-HEPE	Fatty Acyls	-0.04	-0.22	0.14	0.01	6.41E-01
7-Aminoclonazepam	Benzodiazepines	-0.37	-0.53	-0.12	0.51	2.07E-07
Linoleamide	Fatty Acyls	0.36	0.19	0.52	0.23	4.08E-07
Gerberinol	Coumarins and derivatives	-0.09	-0.35	0.12	0.01	2.94E-01
Saccharopine	Carboxylic acids and derivatives	-0.10	-0.28	0.12	0.03	2.45E-01
Pyroglutamic acid	Carboxylic acids and derivatives	-0.20	-0.47	0.06	0.33	8.49E-03
LysoPE(18:1(9Z)/0:0)	Glycerophospholipids	0.40	0.11	0.59	0.36	2.74E-08
Octadecanamide	Fatty Acyls	0.46	0.26	0.58	0.33	3.68E-11
DG(22:5(7Z,10Z,13Z,16Z,19Z)/16:1(9Z)/0:0)	Glycerolipids	-0.22	-0.42	0.02	0.13	3.52E-03
Trimethylaminoacetone	Organooxygen compounds	0.17	0.00	0.31	0.03	2.73E-02
Cholesterol	Steroids and steroid derivatives	-0.14	-0.31	0.14	0.06	8.83E-02
1-Salicylate glucuronide	Organooxygen compounds	-0.46	-0.56	-0.29	0.26	4.69E-11
Racemethionine	NA	-0.07	-0.21	0.15	0.01	4.06E-01
Allysine	Carboxylic acids and derivatives	0.09	-0.12	0.25	0.02	2.96E-01
Pyro-L-glutaminyl-L-glutamine	Carboxylic acids and derivatives	-0.27	-0.60	0.07	0.10	3.08E-04

UDP-N-acetyl-alpha-D-galactosamine	Pyrimidine nucleotides	-0.09	-0.44	0.14	0.03	2.79E-01
4-Amino-2-methylenebutanoic acid	Carboxylic acids and derivatives	-0.02	-0.19	0.14	0.00	8.14E-01
2-Furanmethanol	Heteroaromatic compounds	-0.06	-0.27	0.21	0.01	4.99E-01
Ecgonine	Tropane alkaloids	0.24	0.05	0.47	0.08	1.78E-03
5,6-Dihydroxyindole	Indoles and derivatives	0.06	-0.09	0.21	0.00	4.50E-01
Valyl-Lysine	Carboxylic acids and derivatives	-0.29	-0.44	-0.03	0.08	1.16E-04
AICA-riboside	Imidazole ribonucleosides and ribonucleotides	-0.22	-0.44	-0.03	0.13	3.21E-03
Beta-D-Glucopyranuronic acid	Organooxygen compounds	-0.69	-0.80	-0.36	0.76	2.89E-28
3-Oxohexadecanoyl-CoA	Fatty Acyls	-0.05	-0.24	0.18	0.01	5.60E-01
Homoarecoline		0.11	-0.07	0.26	0.02	1.94E-01
Lacto-N-difucopentaose II	Organooxygen compounds	0.05	-0.14	0.23	0.01	5.52E-01
LysoPE(0:0/22:4(7Z,10Z,13Z,16Z))	Glycerophospholipids	0.39	0.01	0.59	0.38	4.25E-08
alpha-Zearalenol	Macrolides and analogues	-0.49	-0.68	-0.19	0.31	1.26E-12
L-Tyrosine	Carboxylic acids and derivatives	0.03	-0.13	0.17	0.00	7.77E-01
LysoPE(20:1(11Z)/0:0)	Glycerophospholipids	0.12	-0.28	0.39	0.03	1.56E-01
Thiamine	Diazines	-0.23	-0.42	0.02	0.13	2.44E-03
LysoPE(16:0/0:0)	Glycerophospholipids	0.17	-0.09	0.36	0.07	2.90E-02
Guanosine	Purine nucleosides	-0.10	-0.28	0.09	0.02	2.15E-01

LysoPE(0:0/18:3(6Z,9Z,12Z))	Glycerophospholipids	0.27	-0.06	0.50	0.22	2.32E-04
beta-Lactose	Carbohydrates and carbohydrate conjugates	0.31	0.00	0.51	0.38	1.90E-05
3,4,5-Trimethoxycinnamic acid	Cinnamic acids and derivatives	-0.02	-0.28	0.40	0.00	8.20E-01
Delta-12-Prostaglandin J2	Fatty Acyls	-0.37	-0.55	-0.13	1.27	3.57E-07
N-Acetyl-L-alanine	Carboxylic acids and derivatives	-0.08	-0.26	0.11	0.01	3.64E-01
3-Hydroxyisovalerylcarnitine	Fatty Acyls	0.30	0.09	0.55	0.19	4.04E-05
Nicotinic acid	Pyridines and derivatives	-0.05	-0.29	0.16	0.00	5.08E-01
4-Acetyl-3-methylpyridine	Carbonyl compounds	0.02	-0.14	0.18	0.00	8.53E-01
Phosphocreatine	Carboxylic acids and derivatives	0.17	-0.16	0.39	0.26	3.24E-02
Palmitoylethanolamide	Carboximidic acids and derivatives	0.51	0.31	0.63	0.41	1.18E-13
3'-Sialyllactose	Organooxygen compounds	0.12	-0.14	0.41	0.01	1.39E-01
2-Aminoacrylic acid	Carboxylic acids and derivatives	-0.17	-0.49	0.17	0.13	2.75E-02
2,4-Dimethyloxazole	Azoles	0.00	-0.18	0.14	0.00	9.88E-01
LysoPE(0:0/20:4(5Z,8Z,11Z,14Z))	Glycerophospholipids	0.28	-0.03	0.53	0.18	2.05E-04
2-Azetidinecarboxylic acid	NA	-0.30	-0.50	-0.11	0.06	5.43E-05
Epsilon-caprolactam	Lactams	-0.13	-0.31	0.09	0.03	1.00E-01
Polyoxyethylene (600) monoricinoleate	Fatty Acyls	0.56	0.35	0.69	0.66	3.60E-17
1,3-Diisopropylbenzene	Benzene and substituted derivatives	-0.12	-0.31	0.13	0.02	1.34E-01

Triethanolamine	Amines	0.02	-0.15	0.17	0.00	8.15E-01
Aminofructose 6-phosphate	Organooxygen compounds	-0.29	-0.44	-0.07	0.11	7.39E-05
1-Methyl-1,3-cyclohexadiene	Olefins	-0.32	-0.51	-0.02	0.13	1.39E-05
Epidermin	Organooxygen compounds	0.36	0.12	0.52	0.27	7.00E-07
Proline betaine	Carboxylic acids and derivatives	0.14	-0.03	0.32	0.08	8.62E-02
O-Phosphotyrosine	Carboxylic acids and derivatives	-0.08	-0.36	0.11	0.01	3.35E-01
N-Acetylputrescine	Carboximidic acids and derivatives	-0.06	-0.29	0.18	0.01	4.82E-01
Dehydrophytosphingosine	Organonitrogen compounds	0.28	0.12	0.42	0.21	1.43E-04
13S-hydroxyoctadecadienoic acid	Fatty Acyls	-0.20	-0.40	0.05	0.12	7.86E-03
o-Xylene	Benzene and substituted derivatives	-0.22	-0.40	0.05	0.07	4.07E-03
Asparaginyl-Hydroxyproline	Carboxylic acids and derivatives	-0.14	-0.53	0.14	0.05	8.21E-02
Pivaloylcarnitine	Fatty Acyls	0.26	0.08	0.51	0.23	4.93E-04
Norvaline	Carboxylic acids and derivatives	0.08	-0.09	0.24	0.00	3.59E-01
SM(d18:1/16:0)	Sphingolipids	0.71	0.43	0.81	0.87	3.23E-30
(2R)-2-Hydroxy-2-methylbutanenitrile	Organooxygen compounds	0.07	-0.11	0.25	0.01	4.10E-01
Nandrolone	Steroids and steroid derivatives	-0.27	-0.47	0.00	0.53	2.33E-04
Arachidonic acid	Fatty Acyls	-0.31	-0.50	-0.05	0.40	1.90E-05

2-trans-6-cis-Dodecadienal	Organooxygen compounds	-0.08	-0.28	0.17	0.01	3.22E-01
MG(22:2(13Z,16Z)/0:0/0:0)	Glycerolipids	0.43	0.24	0.56	0.43	7.87E-10
D-4'-Phosphopantothenate	Carboxylic acids and derivatives	-0.04	-0.20	0.13	0.00	6.30E-01
Bilirubin	Organoheterocyclic compounds	0.35	0.09	0.48	0.41	9.42E-07
Vinylacetylglycine	Carboxylic acids and derivatives	0.07	-0.12	0.23	0.00	3.86E-01
Alpha-dimorphecolic acid	Fatty Acyls	-0.29	-0.51	0.00	0.35	9.48E-05
(alpha-D-mannosyl)7-beta-D-mannosyl-diacetylchitobiosyl-L-asparagine, isoform A (protein)	Carboxylic acids and derivatives	-0.21	-0.49	0.03	0.03	5.15E-03
Dihydro-6-isopropyl-2,4-dimethyl-4H-1,3,5-dithiazine	Azacyclic compounds	-0.20	-0.38	0.03	0.14	9.59E-03
Cohibin A	Fatty Acyls	0.63	0.33	0.74	1.22	3.34E-22
L-4-Hydroxyglutamate semialdehyde	Carboxylic acids and derivatives	-0.03	-0.23	0.15	0.00	7.77E-01
N2-gamma-Glutamylglutamine	Carboxylic acids and derivatives	-0.07	-0.29	0.12	0.01	4.36E-01
2-Methoxy-3,5-dimethylpyrimidine	Diazines	-0.20	-0.35	-0.04	0.03	1.06E-02
3-Hydroxy-4-butanolide	Organooxygen compounds	-0.07	-0.27	0.14	0.00	4.36E-01
N-Methylnicotinamide	Pyridines and derivatives	0.07	-0.19	0.31	0.01	4.15E-01
N6-Carbamoyl-L-threonyladenosine	Purine nucleosides	0.25	0.00	0.57	0.06	8.08E-04
6-Chloro-N-(1-methylethyl)-1,3,5-triazine-2,4-diamine	Triazines	0.25	0.06	0.42	0.18	8.66E-04
L-alpha-Aspartyl-L-hydroxyproline	NA	0.03	-0.14	0.21	0.00	7.14E-01

Glutaminylaspartic acid	Carboxylic acids and derivatives	-0.20	-0.34	-0.01	0.06	8.03E-03
N-Acetyldopamine	Phenols	0.02	-0.16	0.20	0.00	8.12E-01
1,4-Dideoxy-1,4-imino-D-ribitol	Pyrrolidines	-0.11	-0.25	0.05	0.01	1.64E-01
Butyrylcarnitine	Fatty Acyls	0.16	-0.08	0.50	0.02	4.65E-02
Muricatacin		-0.07	-0.30	0.18	0.01	4.15E-01
Sonchuionoside C	Prenol lipids	-0.08	-0.33	0.14	0.02	3.50E-01
Lactosylceramide (d18:1/16:0)	Sphingolipids	0.42	0.22	0.53	0.42	2.79E-09
N-a-Acetyl-L-arginine	Carboxylic acids and derivatives	0.11	-0.13	0.37	0.01	1.64E-01
Avocadyne 4-acetate	Fatty Acyls	0.02	-0.25	0.31	0.00	8.28E-01
4-(4-Methyl-3-pentenyl)-3-cyclohexene-1-carboxaldehyde	Prenol lipids	-0.21	-0.36	0.01	0.07	4.86E-03
4-Hydroxymandelonitrile	NA	-0.19	-0.49	0.09	0.06	1.45E-02
L-Aspartic acid	Carboxylic acids and derivatives	-0.09	-0.40	0.17	0.05	2.57E-01
LysoPE(0:0/22:1(13Z))	Glycerophospholipids	0.00	-0.31	0.25	0.00	9.90E-01
Quinceoxepine	Ethers	-0.31	-0.50	-0.04	0.26	2.79E-05
1-(beta-D-Ribofuranosyl)-1,4-dihydronicotinamide	NA	-0.16	-0.39	0.30	0.02	4.24E-02
LysoPE(0:0/18:0)	Glycerophospholipids	0.51	0.30	0.67	0.60	6.01E-14
N1-Methyl-4-pyridone-3-carboxamide	Pyridines and derivatives	-0.16	-0.29	0.00	0.04	4.30E-02
Imidazole-4-acetaldehyde	Azoles	-0.17	-0.32	0.00	0.02	2.90E-02
Capsiate	Phenols	-0.36	-0.56	-0.10	0.87	5.24E-07
3-Dehydroxycarnitine	Fatty Acyls	-0.07	-0.45	0.20	0.01	3.91E-01
LysoPE(22:5(4Z,7Z,10Z,13Z,16Z)/0:0)	Glycerophospholipids	0.39	-0.04	0.58	0.40	3.89E-08
Corchorifatty acid D	Fatty Acyls	-0.29	-0.49	-0.02	0.38	7.39E-05
SM(d18:1/24:1(15Z))	Sphingolipids	0.60	0.37	0.71	0.69	4.32E-20

HDMBOA-Glc	Organooxygen compounds	0.06	-0.15	0.28	0.00	4.81E-01
Dieporeticenin	Fatty Acyls	0.14	-0.09	0.36	0.02	7.28E-02
LysoPE(18:2(9Z,12Z)/0:0)	Glycerophospholipids	0.36	0.02	0.60	0.32	8.21E-07
Acrylamide	Carboximidic acids and derivatives	-0.26	-0.38	-0.11	0.13	5.16E-04
N-Acetylneuraminic acid	Organooxygen compounds	-0.11	-0.30	0.09	0.01	1.77E-01
Hydroxyisocaproic acid	Fatty Acyls	-0.16	-0.30	0.03	0.02	3.89E-02
Dihydrouracil	Diazines	-0.37	-0.53	-0.10	0.12	3.64E-07
PC(16:0/16:0)	Glycerophospholipids	0.55	0.32	0.73	0.72	3.28E-16
Indole	Indoles and derivatives	-0.26	-0.46	0.04	0.10	5.27E-04
Formiminoglutamic acid	Carboxylic acids and derivatives	0.03	-0.21	0.27	0.00	7.47E-01
PC(20:0/14:0)	Glycerophospholipids	0.60	0.28	0.73	0.67	6.44E-20
PC(18:1(11Z)/14:0)	Glycerophospholipids	0.78	0.45	0.86	0.89	4.17E-41
LysoPE(22:6(4Z,7Z,10Z,13Z,16Z,19Z)/0:0)	Glycerophospholipids	0.41	0.04	0.57	0.42	7.05E-09
Hypoxanthine	Imidazopyrimidines	-0.20	-0.45	-0.02	0.25	8.73E-03
LysoPE(16:1(9Z)/0:0)	Glycerophospholipids	0.25	-0.09	0.47	0.14	8.49E-04
Glucosamine 6-phosphate	Organooxygen compounds	-0.15	-0.32	0.07	0.03	6.16E-02
2-Methylpiperidine	Piperidines	0.01	-0.13	0.17	0.00	9.11E-01
2-O-(5,8,11,14,17-Eicosapentaenoyl)-1-O-hexadecylglycero-3-phosphocholine	Glycerophospholipids	0.34	0.14	0.48	0.31	3.51E-06
PE(16:0/20:4(5Z,8Z,11Z,14Z))	Glycerophospholipids	0.54	0.31	0.68	0.66	6.44E-16
10-Nitrolinoleic acid	Fatty Acyls	-0.07	-0.19	0.08	0.01	4.06E-01
PE(22:5(4Z,7Z,10Z,13Z,16Z)/16:0)	Glycerophospholipids	0.61	0.39	0.76	0.74	4.02E-21

4-Hydroxy-L-glutamic acid	Organic acids and derivatives	-0.02	-0.24	0.17	0.00	8.14E-01
Pipericine	Fatty Acyls	0.11	-0.06	0.26	0.02	1.95E-01
PE(16:0/18:2(9Z,12Z))	Glycerophospholipids	0.87	0.65	0.90	1.02	1.20E-59
Montecristin	Fatty Acyls	0.87	0.66	0.90	1.08	5.11E-59
(4-Hydroxybenzoyl)choline	Carbonyl compounds	-0.10	-0.26	0.05	0.01	2.15E-01
Deoxyeritadenine	Imidazopyrimidines	0.07	-0.10	0.23	0.00	3.97E-01
PC(18:3(6Z,9Z,12Z)/18:0)	Glycerophospholipids	0.65	0.36	0.79	0.64	1.35E-24
Nephritogenoside	Fatty Acyls	0.37	0.08	0.57	0.27	3.20E-07
o-Ethyltoluene	Benzene and substituted derivatives	-0.27	-0.48	0.04	0.15	2.51E-04
PE(P-18:1(11Z)/18:3(6Z,9Z,12Z))	Glycerophospholipids	0.53	0.33	0.65	0.44	6.24E-15
beta-Alanine	Carboxylic acids and derivatives	-0.25	-0.51	-0.02	0.04	8.74E-04
PC(22:2(13Z,16Z)/P-18:0)	Glycerophospholipids	0.31	0.02	0.49	0.20	2.62E-05
DG(18:4(6Z,9Z,12Z,15Z)/16:1(9Z)/0:0)	Glycerolipids	0.09	-0.22	0.36	0.01	3.00E-01
LysoPC(P-18:1(9Z))	Glycerophospholipids	0.24	0.03	0.39	0.19	1.32E-03
MG(0:0/22:4(7Z,10Z,13Z,16Z)/0:0)	Glycerolipids	0.35	0.13	0.53	0.36	1.10E-06
SM(d16:1/24:1(15Z))	Sphingolipids	0.46	0.28	0.63	0.73	3.18E-11
4-Aminobutyraldehyde	Organooxygen compounds	0.04	-0.14	0.22	0.00	6.22E-01
1-(Malonylamino)cyclopropanecarboxylic acid	Carboxylic acids and derivatives	-0.57	-0.73	-0.30	0.72	9.40E-18
Quercetin	Flavonoids	-0.11	-0.42	0.07	0.04	1.95E-01
N-Acetyl-a-neuraminic acid	Organooxygen compounds	-0.02	-0.22	0.22	0.00	8.04E-01

Ethyl trans-p-methoxycinnamate	Cinnamic acids and derivatives	-0.18	-0.35	0.04	0.29	1.84E-02
4-Oxo-2-nonenal	Organooxygen compounds	-0.17	-0.30	0.01	0.05	3.21E-02
3-Methyl sulfolene	Dihydrothiophenes	-0.06	-0.25	0.15	0.01	4.43E-01
PC(18:2(9Z,12Z)/18:0)	Glycerophospholipids	0.79	0.44	0.87	0.74	2.61E-42
SM(d17:1/24:0)	Sphingolipids	0.36	0.11	0.51	0.28	4.76E-07
L-Octanoylcarnitine	Fatty Acyls	0.31	0.11	0.46	0.10	2.02E-05
gamma-Glutamylglutamic acid	Carboxylic acids and derivatives	0.14	-0.14	0.33	0.05	7.60E-02
Ipomeatetrahydrofuran	Prenol lipids	0.04	-0.22	0.32	0.00	6.15E-01
LysoPE(20:5(5Z,8Z,11Z,14Z,17Z)/0:0)	Glycerophospholipids	0.12	-0.17	0.39	0.04	1.46E-01
Prolyl-Glutamine	Carboxylic acids and derivatives	-0.15	-0.43	0.28	0.03	6.11E-02
PC(22:5(7Z,10Z,13Z,16Z,19Z)/15:0)	Glycerophospholipids	0.64	0.45	0.74	0.95	5.27E-23
PC(20:2(11Z,14Z)/14:0)	Glycerophospholipids	0.77	0.50	0.85	0.67	1.71E-38
PC(20:1(11Z)/14:0)	Glycerophospholipids	0.67	0.31	0.78	0.81	1.41E-26
(R)-2-Hydroxysterculic acid	Fatty Acyls	-0.15	-0.37	0.15	0.05	5.34E-02
Fagomine	Piperidines	-0.20	-0.44	0.01	0.04	9.45E-03
Isoleucyl-Alanine	Carboxylic acids and derivatives	-0.15	-0.32	0.09	0.03	5.18E-02
Glutamyllysine	Carboxylic acids and derivatives	-0.01	-0.18	0.16	0.00	9.38E-01
PC(15:0/15:0)	Glycerophospholipids	0.72	0.42	0.82	1.15	9.96E-32
MG(0:0/18:4(6Z,9Z,12Z,15Z)/0:0)	Glycerolipids	-0.17	-0.41	0.15	0.10	2.90E-02
N2-Maltulosylarginine	Saccharolipids	0.09	-0.08	0.27	0.05	2.54E-01
PC(22:2(13Z,16Z)/16:1(9Z))	Glycerophospholipids	0.77	0.38	0.86	0.85	2.37E-38
SM(d18:1/20:0)	Sphingolipids	0.50	0.29	0.64	0.48	1.91E-13
Kojibiose	Fatty Acyls	-0.10	-0.33	0.18	0.02	2.16E-01

Cytokinin B	Imidazopyrimidines	0.16	0.01	0.44	0.05	3.79E-02
DG(18:4(6Z,9Z,12Z,15Z)/14:1(9Z)/0:0)	Glycerolipids	0.12	-0.21	0.39	0.02	1.53E-01
PC(20:2(11Z,14Z)/20:4(5Z,8Z,11Z,14Z))	Glycerophospholipids	0.72	0.41	0.81	0.97	9.96E-32
SM(d17:1/24:1(15Z))	Sphingolipids	0.72	0.50	0.79	0.78	1.59E-32
SM(d18:0/18:1(9Z))	Sphingolipids	0.59	0.40	0.69	0.66	3.57E-19
5beta-Cholestane-3alpha,7alpha,24,26-tetrol	NA	0.46	0.20	0.61	0.47	6.40E-11
PC(22:5(4Z,7Z,10Z,13Z,16Z)/16:0)	Glycerophospholipids	0.78	0.46	0.86	0.73	5.86E-41
PC(22:5(4Z,7Z,10Z,13Z,16Z)/P-18:0)	Glycerophospholipids	0.40	0.23	0.52	0.53	1.66E-08
PC(24:0/14:1(9Z))	Glycerophospholipids	0.50	0.19	0.66	0.67	1.51E-13
LysoPC(16:0)	Glycerophospholipids	0.20	-0.05	0.38	0.10	8.49E-03
Sialyllacto-N-tetraose b	Organooxygen compounds	-0.07	-0.24	0.14	0.01	3.91E-01
SM(d18:1/14:0)	Sphingolipids	0.43	0.19	0.65	0.49	1.23E-09
PI(22:5(4Z,7Z,10Z,13Z,16Z)/16:0)	Glycerophospholipids	0.59	0.23	0.77	0.45	4.19E-19
Lactapiperanol D	Prenol lipids	-0.25	-0.43	0.01	0.22	7.50E-04
L-Gulonolactone	Lactones	-0.05	-0.22	0.10	0.00	5.22E-01
2,6-Di-tert-butyl-4-ethylphenol	Benzene and substituted derivatives	-0.20	-0.43	0.08	0.12	7.99E-03
PC(18:3(6Z,9Z,12Z)/18:1(11Z))	Glycerophospholipids	0.50	0.26	0.67	0.64	3.05E-13
PC(22:4(7Z,10Z,13Z,16Z)/16:0)	Glycerophospholipids	0.42	0.17	0.64	0.52	1.72E-09
PC(22:5(4Z,7Z,10Z,13Z,16Z)/15:0)	Glycerophospholipids	0.85	0.58	0.90	1.18	4.20E-54
PC(14:0/14:0)	Glycerophospholipids	0.61	0.29	0.73	0.92	4.02E-21
dADP	Purine nucleotides	-0.11	-0.29	0.06	0.01	1.62E-01
PC(20:1(11Z)/20:4(5Z,8Z,11Z,14Z))	Glycerophospholipids	0.80	0.45	0.88	1.03	2.13E-43
3-Benzyl-4-heptanone	Benzene and substituted derivatives	0.04	-0.23	0.30	0.00	6.78E-01

Betonicine	Carboxylic acids and derivatives	0.01	-0.14	0.19	0.00	8.96E-01
Diepomuricanin A	NA	0.07	-0.14	0.32	0.01	3.86E-01
LysoPE(0:0/14:0)	Glycerophospholipids	0.16	-0.18	0.38	0.11	3.59E-02
SM(d18:0/14:0)	Sphingolipids	0.65	0.38	0.73	0.75	7.15E-24
PC(20:2(11Z,14Z)/15:0)	Glycerophospholipids	0.76	0.41	0.85	0.79	3.83E-37
PC(22:2(13Z,16Z)/15:0)	Glycerophospholipids	0.58	0.23	0.71	0.66	1.64E-18
2-(acetylamino)-1,5-anhydro-2-deoxy-3-O-b-D-galactopyranosyl-D-arabino-Hex-1-enitol	Organooxygen compounds	0.15	-0.15	0.35	0.05	6.05E-02
PC(20:3(5Z,8Z,11Z)/20:0)	Glycerophospholipids	0.53	0.25	0.66	0.92	2.62E-15
LysoPC(18:2(9Z,12Z))	Glycerophospholipids	0.45	0.06	0.64	0.46	1.11E-10
Urothion	Pteridines and derivatives	0.07	-0.18	0.26	0.03	4.19E-01
(2'E,4'Z,7'Z,8E)-Colnelenic acid	Fatty Acyls	-0.31	-0.51	-0.05	0.51	2.78E-05
PC(20:0/20:2(11Z,14Z))	Glycerophospholipids	0.54	0.24	0.68	1.09	6.27E-16
Glycerylphosphorylethanolamine	Organic phosphoric acids and derivatives	-0.07	-0.29	0.14	0.01	4.06E-01
6,10,14-Trimethyl-5,9,13-pentadecatrien-2-one	Prenol lipids	-0.27	-0.50	0.03	0.19	2.33E-04
trans-3,4-Dihydro-4,8-dihydroxy-3-methyl-1H-2-benzopyran-1-one	Benzopyrans	-0.06	-0.27	0.18	0.00	4.92E-01
Pyridoxamine	Pyridines and derivatives	-0.05	-0.20	0.12	0.00	5.63E-01
PC(22:4(7Z,10Z,13Z,16Z)/14:0)	Glycerophospholipids	0.71	0.37	0.82	0.75	3.22E-30
PE(20:0/18:4(6Z,9Z,12Z,15Z))	Glycerophospholipids	0.85	0.65	0.89	0.92	8.97E-55
SM(d18:1/18:1(9Z))	Sphingolipids	0.62	0.41	0.72	0.68	1.81E-21
Cohibin C	Fatty Acyls	0.73	0.38	0.85	0.86	3.72E-33
8-Hydroxy-2-octene-4,6-diynoic acid	Fatty Acyls	0.00	-0.19	0.23	0.00	9.98E-01

5-Hydroxy-L-tryptophan	Indoles and derivatives	-0.03	-0.17	0.11	0.00	7.49E-01
PC(20:4(8Z,11Z,14Z,17Z)/P-18:0)	Glycerophospholipids	0.36	0.14	0.50	0.24	4.79E-07
Phenylacetylglutamine	Carboxylic acids and derivatives	0.13	-0.09	0.31	0.02	1.17E-01
Isoeugenitol	Benzopyrans	-0.23	-0.41	-0.01	0.06	2.55E-03
PC(16:1(9Z)/16:1(9Z))	Glycerophospholipids	0.69	0.48	0.78	0.94	1.25E-28
gamma-L-Glutamyl-gamma-L-glutamyl-L-methionine		0.25	-0.01	0.42	0.17	9.93E-04
3-Oxoadipic acid	Keto acids and derivatives	-0.12	-0.33	0.10	0.01	1.52E-01
6-Deoxohomodolichosterone	Steroids and steroid derivatives	0.54	0.30	0.66	0.60	2.08E-15
PC(18:1(9Z)/P-18:1(11Z))	Glycerophospholipids	0.49	0.27	0.60	0.35	1.22E-12
N-Salicyloylaspartic acid	Carboxylic acids and derivatives	-0.27	-0.40	-0.10	0.05	3.08E-04
Vanilpyruvic acid	Benzene and substituted derivatives	0.01	-0.16	0.15	0.00	9.36E-01
PC(22:5(7Z,10Z,13Z,16Z,19Z)/16:1(9Z))	Glycerophospholipids	0.76	0.45	0.83	0.90	6.38E-38
LysoPC(22:6(4Z,7Z,10Z,13Z,16Z,19Z))	Glycerophospholipids	0.40	0.09	0.56	0.34	1.81E-08
Norsanguinarine	Quinolines and derivatives	-0.09	-0.27	0.11	0.01	3.08E-01
3-Methyl-5-pentyl-2-furanundecanoic acid	Fatty Acyls	-0.27	-0.50	0.04	0.13	2.23E-04
3-Aminopropionaldehyde	Organooxygen compounds	-0.32	-0.53	-0.09	0.11	1.53E-05
PC(22:5(7Z,10Z,13Z,16Z,19Z)/16:0)	Glycerophospholipids	0.48	0.24	0.66	0.62	1.91E-12
PC(20:3(8Z,11Z,14Z)/14:0)	Glycerophospholipids	0.52	0.24	0.65	0.62	1.06E-14

Dihydromaleimide	NA	-0.31	-0.52	-0.07	0.15	1.96E-05
2,5-Dioxopentanoate	Keto acids and derivatives	-0.04	-0.23	0.11	0.00	6.57E-01
CS-S-Methylcysteine sulfoxide	NA	-0.15	-0.44	0.08	0.13	5.85E-02
Oxonantenine	Aporphines	0.01	-0.31	0.26	0.00	9.35E-01
DG(14:1(9Z)/18:3(6Z,9Z,12Z)/0:0)	Glycerolipids	-0.04	-0.32	0.27	0.00	6.35E-01
2-Acetylpyridine	Carbonyl compounds	-0.07	-0.24	0.11	0.00	4.31E-01
2-Hydroxy-6,7-dimethoxybenzoxazole	Benzoxazoles	0.23	-0.07	0.54	0.06	2.00E-03
Perillyl acetate	NA	-0.28	-0.45	-0.01	0.25	2.00E-04
N-[(4E,8E)-1,3-dihydroxyoctadeca-4,8-dien-2-yl]hexadecanamide	Sphingolipids	0.25	-0.01	0.41	0.18	8.42E-04
Dihydro-3-(1-octenyl)-2,5-furandione	Oxolanes	-0.25	-0.42	-0.01	0.14	1.07E-03
Artonin Q	Benzopyrans	0.15	-0.04	0.38	0.04	5.10E-02
2-Aminomuconic acid semialdehyde	Carboxylic acids and derivatives	-0.08	-0.26	0.17	0.02	3.57E-01
DG(20:4(8Z,11Z,14Z,17Z)/14:0/0:0)	Glycerolipids	0.02	-0.28	0.28	0.00	8.54E-01
Lacto-N-triose I	Organooxygen compounds	0.20	-0.11	0.39	0.08	9.88E-03
PC(P-18:1(9Z)/14:1(9Z))	Glycerophospholipids	0.36	0.15	0.49	0.27	5.98E-07
(2S,4S)-Pinnatanine	Carboxylic acids and derivatives	0.08	-0.16	0.29	0.00	3.72E-01
O-6-deoxy-a-L-galactopyranosyl-(1->2)-O-b-D-galactopyranosyl-(1->3)-2-(acetylamino)-1,5-anhydro-2-deoxy-D-arabino-Hex-1-enitol	Organooxygen compounds	0.31	0.06	0.47	0.18	2.25E-05
Persicachrome	Prenol lipids	0.48	0.09	0.62	0.49	6.07E-12
L-trans-5-Hydroxy-2-piperidinecarboxylic acid	Carboxylic acids and derivatives	-0.06	-0.23	0.11	0.00	4.64E-01

2-acetyl-1-alkyl-sn-glycero-3-phosphocholine	Glycerophospholipids	0.74	0.56	0.82	1.18	6.17E-35
PC(16:1(9Z)/15:0)	Glycerophospholipids	0.76	0.40	0.87	0.87	1.22E-37
LysoPC(P-16:0)	Glycerophospholipids	0.30	0.07	0.47	0.20	4.62E-05
Phenol glucuronide	Organooxygen compounds	0.19	-0.11	0.41	0.08	1.33E-02
PC(18:3(6Z,9Z,12Z)/15:0)	Glycerophospholipids	0.57	0.33	0.72	0.61	1.27E-17
PI(20:2(11Z,14Z)/18:2(9Z,12Z))	Glycerophospholipids	0.56	0.20	0.72	0.62	3.39E-17
Glycine	Carboxylic acids and derivatives	-0.19	-0.43	0.10	0.04	1.56E-02
DG(14:1(9Z)/18:2(9Z,12Z)/0:0)	Glycerolipids	-0.02	-0.27	0.27	0.00	8.51E-01
Epomusenin A	Fatty Acyls	0.72	0.49	0.80	0.62	1.43E-31
Troxerutin	Flavonoids	0.09	-0.37	0.33	0.02	2.88E-01
LysoPE(20:2(11Z,14Z)/0:0)	Glycerophospholipids	0.21	-0.37	0.52	0.14	6.34E-03
PE(P-18:1(11Z)/22:4(7Z,10Z,13Z,16Z))	Glycerophospholipids	0.52	0.33	0.67	0.54	2.49E-14
PC(22:4(7Z,10Z,13Z,16Z)/15:0)	Glycerophospholipids	0.81	0.52	0.89	1.05	4.53E-45
1-Methylpyrrole	Pyrroles	0.10	-0.06	0.25	0.01	2.10E-01
LysoPC(22:5(4Z,7Z,10Z,13Z,16Z))	Glycerophospholipids	0.36	0.05	0.52	0.53	6.27E-07
5-Hydroxyindoleacetic acid	Indoles and derivatives	-0.11	-0.25	0.05	0.02	1.62E-01
Seryltyrosine	Carboxylic acids and derivatives	0.09	-0.08	0.23	0.01	2.96E-01
5-Hydroxyisourate		-0.12	-0.37	0.14	0.17	1.37E-01
Lacto-N-biose I	Organooxygen compounds	-0.30	-0.48	-0.06	0.12	4.81E-05
PE(P-18:1(11Z)/18:2(9Z,12Z))	Glycerophospholipids	0.36	0.14	0.56	0.29	8.37E-07
3-(3,4-Dihydroxy-5-methoxy)-2-propenoic acid	Cinnamic acids and derivatives	-0.18	-0.32	-0.01	0.02	2.36E-02

4-Hydroxy-3-(16-methylheptadecyl)-2H-pyran-2-one	Pyrans	-0.26	-0.46	0.00	0.18	3.95E-04
LysoPC(20:3(5Z,8Z,11Z))	Glycerophospholipids	0.59	0.36	0.73	0.79	1.92E-19
PI(20:3(5Z,8Z,11Z)/16:0)	Glycerophospholipids	0.55	0.26	0.68	0.89	4.55E-16
1-(3,5-Dihydroxyphenyl)-2-pentadecanone	Phenols	0.02	-0.20	0.22	0.00	8.45E-01
Glucocheirolin	Organooxygen compounds	-0.45	-0.72	-0.27	0.50	7.97E-11
PE(18:1(9Z)/16:0)	Glycerophospholipids	0.58	0.36	0.82	0.73	2.63E-18
5-Hydroxy-7-methoxy-2-tritriacontyl-4H-1-benzopyran-4-one	Benzopyrans	0.37	0.13	0.51	0.39	2.18E-07
DG(14:1(9Z)/22:5(4Z,7Z,10Z,13Z,16Z)/0:0)	Glycerolipids	0.12	-0.09	0.34	0.08	1.49E-01
12,13-EpOME	Fatty Acyls	-0.26	-0.46	0.04	0.17	5.54E-04
PE(20:4(8Z,11Z,14Z,17Z)/P-16:0)	Glycerophospholipids	0.45	0.32	0.69	0.42	1.26E-10
3,5-Dichloro-4-hydroxy-2-methoxy-6-methylbenzoic acid	Benzene and substituted derivatives	-0.18	-0.36	-0.02	0.02	1.84E-02
Hydroxyphenylacetylglycine	Carboxylic acids and derivatives	-0.02	-0.20	0.18	0.00	8.46E-01
DG(22:4(7Z,10Z,13Z,16Z)/18:3(6Z,9Z,12Z)/0:0)	Glycerolipids	-0.24	-0.42	-0.01	0.21	1.15E-03
PC(22:6(4Z,7Z,10Z,13Z,16Z,19Z)/15:0)	Glycerophospholipids	0.77	0.55	0.83	1.04	1.07E-39
PS(14:0/22:2(13Z,16Z))	Glycerophospholipids	0.57	0.21	0.70	0.69	2.04E-17
PC(20:4(8Z,11Z,14Z,17Z)/15:0)	Glycerophospholipids	0.54	0.38	0.68	0.69	9.97E-16
Epiafzelechin 3-gallate		-0.11	-0.49	0.11	0.04	1.66E-01
PE(18:1(11Z)/18:2(9Z,12Z))	Glycerophospholipids	0.46	0.28	0.65	0.61	3.16E-11
PE(P-18:1(9Z)/16:1(9Z))	Glycerophospholipids	0.48	0.35	0.73	0.46	5.96E-12
Demethylated antipyrine	Azoles	-0.19	-0.39	-0.01	0.06	1.58E-02
PE(P-18:1(11Z)/22:5(4Z,7Z,10Z,13Z,16Z))	Glycerophospholipids	0.46	0.28	0.62	0.52	2.74E-11

3-Fucosyllactose	Organooxygen compounds	-0.06	-0.28	0.21	0.01	4.90E-01
LysoPI(18:0/0:0)	Glycerophospholipids	0.26	0.03	0.44	0.10	5.27E-04
Isolinderanolide	Tetrahydrofurans	0.36	0.01	0.60	0.31	4.58E-07
PE(P-18:1(9Z)/20:3(5Z,8Z,11Z))	Glycerophospholipids	0.47	0.31	0.68	0.47	1.73E-11
Marmesin rhamnoside	Coumarins and derivatives	0.19	0.01	0.43	0.03	1.58E-02
1-Isothiocyanato-6-(methylthio)hexane	Isothiocyanates	0.13	-0.08	0.29	0.02	9.35E-02
PE(P-18:1(9Z)/20:5(5Z,8Z,11Z,14Z,17Z))	Glycerophospholipids	0.66	0.41	0.77	0.66	2.37E-25
PC(P-16:0/18:4(6Z,9Z,12Z,15Z))	Glycerophospholipids	0.66	0.45	0.75	0.77	3.58E-25
Obtusilactone A	Tetrahydrofurans	0.08	-0.20	0.35	0.01	3.46E-01
PE(P-18:1(9Z)/18:1(9Z))	Glycerophospholipids	0.53	0.38	0.72	0.48	6.71E-15
PE(22:6(4Z,7Z,10Z,13Z,16Z,19Z)/P-18:1(11Z))	Glycerophospholipids	0.62	0.31	0.71	0.51	3.15E-21
PE(20:4(5Z,8Z,11Z,14Z)/P-18:1(11Z))	Glycerophospholipids	0.81	0.53	0.86	0.66	1.80E-45
Cohibin B	Fatty Acyls	0.20	0.00	0.34	0.06	9.83E-03
Dide-O-methyl-4-O-alpha-D-glucopyranosylsimmondsin	Organooxygen compounds	0.14	-0.11	0.31	0.02	7.91E-02
PC(P-18:1(9Z)/16:1(9Z))	Glycerophospholipids	0.02	-0.13	0.19	0.00	7.91E-01
1-Isothiocyanato-6-(methylsulfinyl)hexane	Sulfoxides	0.18	0.03	0.29	0.04	1.86E-02
PC(20:3(8Z,11Z,14Z)/15:0)	Glycerophospholipids	0.84	0.56	0.88	1.07	7.18E-52
3-Isopropenylpentanedioic acid	Fatty Acyls	0.09	-0.16	0.49	0.00	2.85E-01
PE(P-16:0/20:4(5Z,8Z,11Z,14Z))	Glycerophospholipids	0.78	0.53	0.87	0.74	8.74E-40
Glaucarubin	Prenol lipids	0.05	-0.20	0.28	0.01	5.72E-01
PE(22:2(13Z,16Z)/18:1(11Z))	Glycerophospholipids	0.69	0.35	0.78	0.84	5.11E-28
PE(P-18:0/18:2(9Z,12Z))	Glycerophospholipids	0.72	0.49	0.81	0.63	3.28E-32
Melleolide M	Prenol lipids	-0.07	-0.32	0.15	0.02	4.27E-01
PE(P-18:0/22:4(7Z,10Z,13Z,16Z))	Glycerophospholipids	0.68	0.41	0.79	0.70	2.75E-27

Metiamide	Azoles	-0.29	-0.48	-0.01	0.14	8.92E-05
PS(18:0/22:6(4Z,7Z,10Z,13Z,16Z,19Z))	Glycerophospholipids	0.53	0.21	0.70	0.83	2.89E-15

Table S5. Details of PC3 results

Metabolites	Class	PCA loadings	Bootstrap CI of loadings		Contribution (%)	FDR
			Lower	Upper		
3,3,5-triiodo-L-thyronine-beta-D-glucuronoside	Carboxylic acids and derivatives	0.17	-0.13	0.41	0.04	3.65E-02
Cellobiose	Organooxygen compounds	-0.06	-0.24	0.17	0.01	5.24E-01
Adenine	Imidazopyrimidines	0.08	-0.23	0.28	0.02	3.46E-01
1-Amino-propan-2-ol	Organonitrogen compounds	0.03	-0.29	0.40	0.00	7.70E-01
1-Pyrroline	Pyrrolines	0.16	-0.18	0.34	0.05	4.76E-02
Rheidin A	Anthracenes	0.10	-0.26	0.43	0.03	2.39E-01
1-Kestose	Organooxygen compounds	-0.18	-0.36	0.07	0.06	2.33E-02
Niacinamide	Pyridines and derivatives	0.12	-0.16	0.34	0.05	1.40E-01
5-Methylcytidine	Pyrimidine nucleosides	0.36	0.02	0.60	0.21	7.23E-07
Yuccaol C	2-arylbenzofuran flavonoids	-0.18	-0.38	0.10	0.06	2.19E-02
Adenosine	Purine nucleosides	0.33	0.07	0.42	0.44	1.34E-05
1-Methylinosine	Purine nucleosides	0.20	-0.24	0.46	0.08	1.24E-02
Choline	Organonitrogen compounds	0.16	-0.35	0.54	0.03	4.78E-02
D-Proline	Carboxylic acids and derivatives	-0.06	-0.32	0.33	0.00	5.45E-01

1H-Pyrrole-2-carboxaldehyde	Organooxygen compounds	0.00	-0.24	0.20	0.00	9.67E-01
4-Aminophenol	Benzene and substituted derivatives	0.02	-0.19	0.19	0.00	8.15E-01
Pyrrolidonecarboxylic acid	Carboxylic acids and derivatives	0.34	-0.04	0.56	0.20	5.85E-06
Uracil	Diazines	0.27	-0.13	0.52	0.17	4.49E-04
5-Aminopentanoic acid	Carboxylic acids and derivatives	-0.38	-0.51	0.00	0.38	2.31E-07
5,12-dihydroxy-6,8,10,14,17-eicosapentaenoic acid	NA	0.30	-0.02	0.43	1.02	5.85E-05
Kynurenic acid	Quinolines and derivatives	-0.11	-0.36	0.17	0.04	1.72E-01
Cytidine monophosphate	Pyrimidine nucleotides	0.23	-0.22	0.57	0.16	2.83E-03
N2,N2-Dimethylguanosine	Purine nucleosides	-0.02	-0.42	0.37	0.00	8.51E-01
Gonyautoxin VI	Saxitoxins, gonyautoxins, and derivatives	0.25	-0.22	0.55	0.14	1.40E-03
4-(3,4-Dihydroxyphenyl)-2-hydroxy-1H-phenalen-1-one	Naphthalenes	0.04	-0.15	0.19	0.00	7.07E-01
Guanine	Imidazopyrimidines	0.42	0.08	0.54	0.54	6.52E-09
Nicotinamide ribotide	NA	-0.07	-0.50	0.48	0.03	4.18E-01
Prolyl-Aspartate	Carboxylic acids and derivatives	-0.26	-0.56	0.23	0.12	8.61E-04
1-Methylnicotinamide	Pyridines and derivatives	-0.23	-0.36	0.03	0.08	2.69E-03

Pipecolic acid	Carboxylic acids and derivatives	-0.01	-0.40	0.35	0.00	8.88E-01
N-Ornithyl-L-taurine	Carboxylic acids and derivatives	-0.10	-0.35	0.23	0.03	2.39E-01
D-Maltose	Organooxygen compounds	0.37	-0.04	0.52	0.25	3.73E-07
2-O-(6-Phospho-alpha-mannosyl)-D-glycerate	Organooxygen compounds	0.00	-0.20	0.17	0.00	9.91E-01
Taurine	Organic sulfonic acids and derivatives	-0.04	-0.24	0.24	0.00	7.07E-01
2-O-Galloylsucrose	Benzene and substituted derivatives	0.27	-0.20	0.53	0.35	4.92E-04
L-Asparagine	Carboxylic acids and derivatives	0.13	-0.16	0.29	0.05	1.19E-01
Pyrrolidine	Pyrrolidines	0.05	-0.17	0.24	0.01	6.22E-01
Protoanemonin	Dihydrofurans	-0.43	-0.61	0.13	0.44	2.56E-09
Xanthotoxol glucoside	Coumarins and derivatives	-0.18	-0.46	0.21	0.07	2.52E-02
L-Threonine	Carboxylic acids and derivatives	0.32	-0.01	0.46	0.21	1.51E-05
1-Methyladenosine	Purine nucleosides	0.34	-0.03	0.51	0.25	3.36E-06
Propionylcarnitine	Fatty Acyls	-0.36	-0.52	0.01	0.25	1.37E-06
Phenylacetaldehyde	Benzene and substituted derivatives	-0.22	-0.57	0.08	0.13	4.28E-03
N4-Acetylcytidine	Pyrimidine nucleosides	0.40	0.11	0.66	0.71	4.86E-08
2,5-Dihydro-2,4-dimethyloxazole	Azolines	-0.02	-0.16	0.14	0.00	8.66E-01

3-Methylhistidine	Carboxylic acids and derivatives	0.01	-0.26	0.29	0.00	8.76E-01
Sphinganine	Organonitrogen compounds	-0.04	-0.22	0.14	0.02	6.78E-01
Chondroitin	Organooxygen compounds	0.12	-0.18	0.38	0.02	1.59E-01
2-Methylfuran	Heteroaromatic compounds	-0.39	-0.56	0.14	2.18	1.24E-07
trans-3,3',4',5,5',7-Hexahydroxyflavanone	NA	-0.02	-0.21	0.16	0.00	8.67E-01
Acetylhydrazine	Carboxylic acids and derivatives	0.36	-0.02	0.52	0.19	1.37E-06
Turanose	Fatty Acyls	0.18	-0.10	0.37	0.05	2.40E-02
L-Palmitoylcarnitine	Fatty Acyls	0.01	-0.18	0.26	0.00	8.82E-01
Lacto-N-fucopentaose III	Organooxygen compounds	0.29	-0.09	0.52	0.25	1.04E-04
3-Acetamidobutanal	Organooxygen compounds	0.14	-0.10	0.31	0.03	8.76E-02
4-Aminohippuric acid	Benzene and substituted derivatives	-0.28	-0.44	0.03	0.15	2.76E-04
L-Phenylalanine	Carboxylic acids and derivatives	0.22	-0.07	0.45	0.08	4.35E-03
Methyl 2-furoate	Furans	-0.50	-0.65	0.12	0.49	1.59E-12
N-Hexadecanoylpyrrolidine	Pyrrolidines	0.12	-0.23	0.41	0.05	1.60E-01
1-Methylhypoxanthine	NA	0.17	-0.27	0.44	0.06	3.00E-02
erythro-Isoleucine	NA	0.05	-0.22	0.32	0.00	5.89E-01
L-Acetylcarnitine	Fatty Acyls	-0.43	-0.64	0.00	0.16	3.50E-09
Trigonelline		-0.18	-0.32	0.08	0.16	2.06E-02

D-Serine	Carboxylic acids and derivatives	0.27	-0.08	0.53	0.17	4.20E-04
1-Methylguanosine	Purine nucleosides	0.11	-0.21	0.40	0.02	1.86E-01
Uridine	Pyrimidine nucleosides	0.25	-0.17	0.52	0.15	9.31E-04
2,5-Dimethyloxazole	Azoles	-0.43	-0.64	0.11	0.55	1.57E-09
Adenosine 2'-phosphate	Organooxygen compounds	0.26	-0.32	0.72	0.44	5.70E-04
Cytidine	Pyrimidine nucleosides	0.08	-0.16	0.30	0.02	3.93E-01
5'-Methylthioadenosine	5'-deoxyribonucleosides	0.16	-0.06	0.41	0.08	5.20E-02
L-Lysine	Carboxylic acids and derivatives	0.00	-0.34	0.33	0.00	9.81E-01
Piperidine	Piperidines	0.14	-0.13	0.33	0.03	9.82E-02
3-Methylguanine	Imidazopyrimidines	0.19	-0.26	0.44	0.10	1.84E-02
Thiamine monophosphate	Diazines	0.11	-0.39	0.60	0.03	1.72E-01
Sorbose 1-phosphate	Organooxygen compounds	0.04	-0.17	0.20	0.00	6.63E-01
Trimethylamine N-oxide	Organonitrogen compounds	-0.19	-0.34	0.12	0.10	1.74E-02
D-Glutamine	Carboxylic acids and derivatives	0.29	-0.01	0.48	0.33	9.70E-05
2-Butenal	NA	-0.44	-0.65	0.15	0.92	6.24E-10
Styrene	Benzene and substituted derivatives	0.24	-0.03	0.37	0.14	1.74E-03
Anserine	Peptidomimetics	0.11	-0.11	0.25	0.05	2.05E-01

Oleamide	Fatty Acyls	-0.04	-0.29	0.28	0.00	6.63E-01
Solasodine	Steroids and steroid derivatives	0.27	0.00	0.41	0.82	3.33E-04
Dihydroneopterin phosphate	Pteridines and derivatives	0.22	-0.43	0.54	0.11	5.31E-03
Diethanolamine	Organonitrogen compounds	0.17	-0.06	0.33	0.06	2.98E-02
2-Fucosyllactose	Organooxygen compounds	-0.38	-0.62	0.29	0.64	2.96E-07
Nicotine	Pyridines and derivatives	0.02	-0.17	0.17	0.00	8.67E-01
Spinacetin 3-gentiobioside	Flavonoids	-0.45	-0.65	0.18	2.02	5.24E-10
Citrulline	Carboxylic acids and derivatives	0.31	-0.05	0.47	0.21	2.87E-05
4-Hydroxybenzaldehyde	Organooxygen compounds	0.04	-0.15	0.31	0.01	6.79E-01
Prolyl-Arginine	Carboxylic acids and derivatives	-0.01	-0.37	0.27	0.00	9.08E-01
Pantothenic acid	Alcohols and polyols	-0.05	-0.25	0.12	0.01	6.22E-01
L-Valine	Carboxylic acids and derivatives	0.34	-0.07	0.50	0.17	3.95E-06
3-Amino-2-piperidone	Carboxylic acids and derivatives	-0.08	-0.25	0.13	0.01	3.48E-01
Uralenneoside	Benzene and substituted derivatives	-0.20	-0.41	0.21	0.08	9.45E-03
Lactaldehyde	Organooxygen compounds	0.05	-0.25	0.36	0.01	6.22E-01

Mesalazine	Benzene and substituted derivatives	-0.06	-0.21	0.14	0.01	5.40E-01
Muscomin	Homoisoflavonoids	0.29	0.01	0.44	0.13	9.32E-05
Urocanic acid	Azoles	0.03	-0.21	0.26	0.01	7.19E-01
Dimethyl dialkyl ammonium chloride	Quaternary ammonium salts	0.27	-0.11	0.47	0.99	3.21E-04
NAD	(5'->5')-dinucleotides	0.35	-0.04	0.55	0.49	3.11E-06
Sialyl-Lewis X	Organooxygen compounds	0.30	0.00	0.50	0.33	6.46E-05
N-Acetylhistidine	Carboxylic acids and derivatives	-0.16	-0.38	0.13	0.05	4.84E-02
3alpha,4,5,7alpha-Tetrahydro-5-hydroxy-1H-isoindole-1,3(2H)-dione	Isoindoles and derivatives	0.43	0.13	0.57	0.48	1.57E-09
2-Acetyl-3,6-dimethylpyrazine	Carbonyl compounds	-0.01	-0.20	0.18	0.00	8.88E-01
Homo-L-arginine	Carboxylic acids and derivatives	0.34	-0.12	0.51	0.36	4.70E-06
3,4',5,6-Tetrahydroxy-3',7-dimethoxyflavone 3-glucuronide	Flavonoids	0.17	-0.23	0.46	0.12	2.96E-02
L-Arginine	Carboxylic acids and derivatives	0.14	-0.37	0.49	0.05	7.55E-02
Ornithine	Carboxylic acids and derivatives	-0.15	-0.28	0.05	0.06	6.20E-02
Biliverdin	Tetrapyrroles and derivatives	0.06	-0.20	0.26	0.02	5.20E-01
4,8 Dimethylnonanoyl carnitine	Fatty Acyls	0.12	-0.10	0.27	0.11	1.33E-01

S-Adenosylmethionine	5'-deoxyribonucleosides	0.37	-0.01	0.64	0.46	5.94E-07
1,2,3,4-Tetrahydro-b-carboline-1,3-dicarboxylic acid	Harmala alkaloids	0.04	-0.11	0.19	0.00	6.87E-01
Spinatoside	Flavonoids	0.29	-0.17	0.55	0.38	1.12E-04
5-Methyl-2-furancarboxaldehyde	Carbonyl compounds	-0.42	-0.62	0.15	2.99	5.20E-09
Tryptophan	NA	0.11	-0.12	0.39	0.02	1.80E-01
O-Acetylserine	Carboxylic acids and derivatives	0.44	0.09	0.61	0.23	6.24E-10
(9S,10E,12Z,15Z)-9-Hydroxy-10,12,15-octadecatrienoic acid	Lineolic acids and derivatives	0.28	-0.02	0.40	0.43	2.89E-04
FAPy-adenine	Diazines	0.16	-0.15	0.40	0.09	4.80E-02
5-HEPE	Fatty Acyls	-0.02	-0.20	0.15	0.01	8.21E-01
7-Aminoclonazepam	Benzodiazepines	-0.10	-0.40	0.39	0.06	2.56E-01
Linoleamide	Fatty Acyls	0.03	-0.21	0.29	0.00	7.94E-01
Gerberinol	Coumarins and derivatives	0.06	-0.20	0.36	0.01	4.83E-01
Saccharopine	Carboxylic acids and derivatives	-0.08	-0.31	0.22	0.03	3.86E-01
Pyroglutamic acid	Carboxylic acids and derivatives	0.32	0.02	0.50	1.32	1.85E-05
LysoPE(18:1(9Z)/0:0)	Glycerophospholipids	0.46	0.03	0.67	0.77	1.79E-10
Octadecanamide	Fatty Acyls	0.01	-0.28	0.37	0.00	8.72E-01
DG(22:5(7Z,10Z,13Z,16Z,19Z)/16:1(9Z)/0:0)	Glycerolipids	0.26	0.02	0.36	0.30	5.55E-04

Trimethylaminoacetone	Organooxygen compounds	-0.13	-0.26	0.11	0.03	1.02E-01
Cholesterol	Steroids and steroid derivatives	-0.22	-0.40	0.08	0.25	5.31E-03
1-Salicylate glucuronide	Organooxygen compounds	-0.04	-0.27	0.13	0.00	6.71E-01
Racemethionine	NA	0.16	-0.03	0.32	0.05	5.22E-02
Allysine	Carboxylic acids and derivatives	0.04	-0.19	0.25	0.01	6.75E-01
Pyro-L-glutaminyl-L-glutamine	Carboxylic acids and derivatives	0.43	0.04	0.64	0.41	2.42E-09
UDP-N-acetyl-alpha-D-galactosamine	Pyrimidine nucleotides	0.29	-0.09	0.59	0.45	1.13E-04
4-Amino-2-methylenebutanoic acid	Carboxylic acids and derivatives	-0.04	-0.19	0.11	0.00	7.08E-01
2-Furanmethanol	Heteroaromatic compounds	-0.47	-0.65	0.03	0.88	5.72E-11
Ecgonine	Tropane alkaloids	-0.20	-0.37	0.09	0.09	1.35E-02
5,6-Dihydroxyindole	Indoles and derivatives	-0.04	-0.21	0.18	0.00	6.87E-01
Valyl-Lysine	Carboxylic acids and derivatives	0.09	-0.32	0.39	0.01	2.81E-01
AICA-riboside	Imidazole ribonucleosides and ribonucleotides	0.19	-0.05	0.35	0.15	1.79E-02
Beta-D-Glucopyranuronic acid	Organooxygen compounds	0.02	-0.52	0.32	0.00	8.63E-01
3-Oxohexadecanoyl-CoA	Fatty Acyls	-0.04	-0.35	0.42	0.01	6.71E-01
Homoarecoline		-0.13	-0.25	0.08	0.04	1.14E-01

Lacto-N-difucopentaose II	Organooxygen compounds	-0.16	-0.38	0.23	0.09	5.15E-02
LysoPE(0:0/22:4(7Z,10Z,13Z,16Z))	Glycerophospholipids	0.59	0.11	0.69	1.35	6.22E-18
alpha-Zearalenol	Macrolides and analogues	0.34	0.00	0.52	0.25	3.36E-06
L-Tyrosine	Carboxylic acids and derivatives	0.14	-0.15	0.42	0.07	9.82E-02
LysoPE(20:1(11Z)/0:0)	Glycerophospholipids	0.58	0.14	0.68	1.39	6.70E-18
Thiamine	Diazines	0.23	-0.23	0.52	0.22	3.12E-03
LysoPE(16:0/0:0)	Glycerophospholipids	0.25	-0.08	0.45	0.24	9.88E-04
Guanosine	Purine nucleosides	0.06	-0.21	0.26	0.01	5.28E-01
LysoPE(0:0/18:3(6Z,9Z,12Z))	Glycerophospholipids	0.58	0.21	0.67	1.58	6.53E-18
beta-Lactose	Carbohydrates and carbohydrate conjugates	-0.42	-0.58	0.06	1.08	6.26E-09
3,4,5-Trimethoxycinnamic acid	Cinnamic acids and derivatives	-0.32	-0.65	0.37	0.25	1.71E-05
Delta-12-Prostaglandin J2	Fatty Acyls	0.10	-0.16	0.25	0.15	2.51E-01
N-Acetyl-L-alanine	Carboxylic acids and derivatives	-0.07	-0.24	0.15	0.01	4.50E-01
3-Hydroxyisovalerylcarnitine	Fatty Acyls	-0.15	-0.44	0.29	0.07	7.42E-02
Nicotinic acid	Pyridines and derivatives	-0.04	-0.39	0.24	0.00	6.43E-01
4-Acetyl-3-methylpyridine	Carbonyl compounds	0.04	-0.18	0.36	0.00	7.10E-01

Phosphocreatine	Carboxylic acids and derivatives	-0.24	-0.67	0.42	0.86	1.84E-03
Palmitoylethanolamide	Carboximidic acids and derivatives	-0.05	-0.27	0.25	0.01	6.22E-01
3'-Sialyllactose	Organooxygen compounds	-0.11	-0.51	0.40	0.02	1.71E-01
2-Aminoacrylic acid	Carboxylic acids and derivatives	0.32	-0.11	0.61	0.73	1.98E-05
2,4-Dimethyloxazole	Azoles	-0.09	-0.32	0.14	0.01	2.98E-01
LysoPE(0:0/20:4(5Z,8Z,11Z,14Z))	Glycerophospholipid s	0.63	0.22	0.72	1.49	1.41E-20
2-Azetidinecarboxylic acid	NA	0.28	0.00	0.42	0.09	1.91E-04
Epsilon-caprolactam	Lactams	0.15	-0.19	0.36	0.06	6.94E-02
Polyoxyethylene (600) monoricinoleate	Fatty Acyls	0.07	-0.26	0.36	0.02	4.54E-01
1,3-Diisopropylbenzene	Benzene and substituted derivatives	0.33	-0.02	0.49	0.25	6.47E-06
Triethanolamine	Amines	0.14	-0.09	0.27	0.11	8.69E-02
Aminofructose 6-phosphate	Organooxygen compounds	0.15	-0.23	0.43	0.05	6.20E-02
1-Methyl-1,3-cyclohexadiene	Olefins	0.36	-0.08	0.53	0.27	9.83E-07
Epidermin	Organooxygen compounds	-0.30	-0.43	0.01	0.31	5.09E-05
Proline betaine	Carboxylic acids and derivatives	-0.04	-0.21	0.25	0.01	7.10E-01
O-Phosphotyrosine	Carboxylic acids and derivatives	0.00	-0.39	0.42	0.00	9.74E-01
N-Acetylputrescine	Carboximidic acids and derivatives	0.43	0.07	0.55	0.50	1.57E-09

Dehydrophytosphingosine	Organonitrogen compounds	0.02	-0.19	0.25	0.00	8.65E-01
13S-hydroxyoctadecadienoic acid	Fatty Acyls	0.14	-0.06	0.27	0.09	9.20E-02
o-Xylene	Benzene and substituted derivatives	0.24	-0.13	0.42	0.15	1.72E-03
Asparaginyl-Hydroxyproline	Carboxylic acids and derivatives	0.15	-0.37	0.59	0.09	6.80E-02
Pivaloylcarnitine	Fatty Acyls	-0.16	-0.44	0.28	0.14	4.49E-02
Norvaline	Carboxylic acids and derivatives	-0.04	-0.19	0.12	0.00	6.63E-01
SM(d18:1/16:0)	Sphingolipids	-0.07	-0.35	0.40	0.01	4.55E-01
(2R)-2-Hydroxy-2-methylbutanenitrile	Organooxygen compounds	-0.06	-0.26	0.13	0.01	4.92E-01
Nandrolone	Steroids and steroid derivatives	0.23	-0.04	0.36	0.59	3.27E-03
Arachidonic acid	Fatty Acyls	0.22	-0.07	0.33	0.33	4.23E-03
2-trans-6-cis-Dodecadienal	Organooxygen compounds	0.33	-0.01	0.49	0.30	7.23E-06
MG(22:2(13Z,16Z)/0:0/0:0)	Glycerolipids	-0.10	-0.31	0.25	0.04	2.42E-01
D-4'-Phosphopantothenate	Carboxylic acids and derivatives	0.03	-0.15	0.24	0.00	7.12E-01
Bilirubin	Organoheterocyclic compounds	0.20	-0.07	0.41	0.21	1.24E-02
Vinylacetylglycine	Carboxylic acids and derivatives	-0.02	-0.31	0.24	0.00	8.21E-01
Alpha-dimorphecolic acid	Fatty Acyls	0.34	0.03	0.43	0.80	3.57E-06

(alpha-D-mannosyl)7-beta-D-mannosyl-diacetylchitobiosyl-L-asparagine, isoform A (protein)	Carboxylic acids and derivatives	0.39	0.11	0.54	0.16	9.34E-08
Dihydro-6-isopropyl-2,4-dimethyl-4H-1,3,5-dithiazine	Azacyclic compounds	-0.03	-0.16	0.11	0.00	8.03E-01
Cohibin A	Fatty Acyls	-0.05	-0.37	0.38	0.01	6.22E-01
L-4-Hydroxyglutamate semialdehyde	Carboxylic acids and derivatives	-0.10	-0.30	0.10	0.02	2.39E-01
N2-gamma-Glutamylglutamine	Carboxylic acids and derivatives	0.06	-0.11	0.25	0.02	4.90E-01
2-Methoxy-3,5-dimethylpyrimidine	Diazines	0.08	-0.17	0.23	0.01	3.71E-01
3-Hydroxy-4-butanolide	Organooxygen compounds	-0.21	-0.43	0.13	0.05	6.17E-03
N-Methylnicotinamide	Pyridines and derivatives	0.16	-0.28	0.43	0.06	5.08E-02
N6-Carbamoyl-L-threonyladenosine	Purine nucleosides	-0.42	-0.61	-0.02	0.26	4.79E-09
6-Chloro-N-(1-methylethyl)-1,3,5-triazine-2,4-diamine	Triazines	-0.19	-0.32	0.03	0.17	1.79E-02
L-alpha-Aspartyl-L-hydroxyproline	NA	-0.07	-0.34	0.32	0.02	4.50E-01
Glutaminylaspartic acid	Carboxylic acids and derivatives	0.13	-0.12	0.30	0.04	1.02E-01
N-Acetyldopamine	Phenols	-0.22	-0.36	0.01	0.48	4.80E-03
1,4-Dideoxy-1,4-imino-D-ribitol	Pyrrolidines	-0.03	-0.17	0.11	0.00	7.19E-01
Butyrylcarnitine	Fatty Acyls	-0.22	-0.57	0.23	0.05	4.64E-03
Muricatacin		0.46	0.08	0.64	0.65	1.15E-10
Sonchuionoside C	Prenol lipids	0.28	0.01	0.45	0.46	1.69E-04
Lactosylceramide (d18:1/16:0)	Sphingolipids	-0.07	-0.28	0.23	0.02	4.14E-01
N-a-Acetyl-L-arginine	Carboxylic acids and derivatives	-0.37	-0.50	-0.08	0.17	3.28E-07

Avocadyne 4-acetate	Fatty Acyls	0.55	0.10	0.72	1.13	5.74E-16
4-(4-Methyl-3-pentenyl)-3-cyclohexene-1-carboxaldehyde	Prenol lipids	0.25	-0.10	0.43	0.15	1.44E-03
4-Hydroxymandelonitrile	NA	0.44	0.13	0.57	0.51	7.58E-10
L-Aspartic acid	Carboxylic acids and derivatives	0.34	0.03	0.54	1.06	6.00E-06
LysoPE(0:0/22:1(13Z))	Glycerophospholipid s	0.47	0.06	0.60	1.54	5.36E-11
Quinceoxepine	Ethers	0.18	-0.10	0.31	0.15	2.16E-02
1-(beta-D-Ribofuranosyl)-1,4-dihydronicotinamide	NA	-0.22	-0.66	0.31	0.05	5.15E-03
LysoPE(0:0/18:0)	Glycerophospholipid s	0.11	-0.19	0.39	0.05	1.83E-01
N1-Methyl-4-pyridone-3-carboxamide	Pyridines and derivatives	0.03	-0.18	0.23	0.00	7.66E-01
Imidazole-4-acetaldehyde	Azoles	0.08	-0.24	0.30	0.01	3.48E-01
Capsiate	Phenols	0.19	-0.10	0.32	0.40	1.50E-02
3-Dehydroxycarnitine	Fatty Acyls	0.07	-0.33	0.52	0.01	4.73E-01
LysoPE(22:5(4Z,7Z,10Z,13Z,16Z)/0:0)	Glycerophospholipid s	0.51	0.06	0.69	1.08	4.02E-13
Corchorifatty acid D	Fatty Acyls	0.21	-0.07	0.32	0.32	6.24E-03
SM(d18:1/24:1(15Z))	Sphingolipids	0.09	-0.20	0.39	0.03	2.80E-01
HDMBOA-Glc	Organooxygen compounds	0.13	-0.22	0.36	0.03	1.28E-01
Dieporeticenin	Fatty Acyls	0.38	0.09	0.65	0.29	1.42E-07
LysoPE(18:2(9Z,12Z)/0:0)	Glycerophospholipid s	0.61	0.22	0.71	1.51	2.65E-19
Acrylamide	Carboximidic acids and derivatives	-0.02	-0.23	0.17	0.00	8.15E-01

N-Acetylneuraminic acid	Organooxygen compounds	-0.13	-0.33	0.18	0.02	1.15E-01
Hydroxyisocaproic acid	Fatty Acyls	0.02	-0.23	0.23	0.00	8.67E-01
Dihydrouracil	Diazines	0.27	-0.06	0.42	0.11	3.60E-04
PC(16:0/16:0)	Glycerophospholipids	0.05	-0.19	0.45	0.01	6.22E-01
Indole	Indoles and derivatives	0.32	-0.04	0.48	0.25	1.71E-05
Formiminoglutamic acid	Carboxylic acids and derivatives	-0.42	-0.55	-0.06	0.25	4.18E-09
PC(20:0/14:0)	Glycerophospholipids	-0.07	-0.38	0.41	0.01	4.79E-01
PC(18:1(11Z)/14:0)	Glycerophospholipids	0.18	-0.12	0.61	0.07	2.52E-02
LysoPE(22:6(4Z,7Z,10Z,13Z,16Z,19Z)/0:0)	Glycerophospholipids	0.42	0.01	0.62	0.72	4.18E-09
Hypoxanthine	Imidazopyrimidines	0.28	-0.02	0.47	0.75	2.96E-04
LysoPE(16:1(9Z)/0:0)	Glycerophospholipids	0.47	0.08	0.59	0.80	5.82E-11
Glucosamine 6-phosphate	Organooxygen compounds	0.12	-0.26	0.39	0.03	1.69E-01
2-Methylpiperidine	Piperidines	0.02	-0.22	0.22	0.00	8.27E-01
2-O-(5,8,11,14,17-Eicosapentaenoyl)-1-O-hexadecylglycero-3-phosphocholine	Glycerophospholipids	0.04	-0.16	0.25	0.01	7.10E-01
PE(16:0/20:4(5Z,8Z,11Z,14Z))	Glycerophospholipids	0.16	-0.07	0.38	0.10	4.38E-02
10-Nitrolinoleic acid	Fatty Acyls	0.05	-0.15	0.21	0.01	6.20E-01
PE(22:5(4Z,7Z,10Z,13Z,16Z)/16:0)	Glycerophospholipids	0.09	-0.14	0.36	0.02	3.34E-01

4-Hydroxy-L-glutamic acid	Organic acids and derivatives	0.13	-0.02	0.24	0.09	1.21E-01
Pipericine	Fatty Acyls	0.01	-0.15	0.19	0.00	8.88E-01
PE(16:0/18:2(9Z,12Z))	Glycerophospholipids	0.12	-0.16	0.49	0.03	1.51E-01
Montecristin	Fatty Acyls	0.12	-0.18	0.49	0.03	1.64E-01
(4-Hydroxybenzoyl)choline	Carbonyl compounds	-0.08	-0.29	0.14	0.01	3.81E-01
Deoxyeritadenine	Imidazopyrimidines	0.16	-0.05	0.29	0.04	4.62E-02
PC(18:3(6Z,9Z,12Z)/18:0)	Glycerophospholipids	0.39	0.04	0.58	0.36	1.35E-07
Nephritogenoside	Fatty Acyls	-0.42	-0.55	-0.03	0.57	3.90E-09
o-Ethyltoluene	Benzene and substituted derivatives	0.36	-0.05	0.51	0.40	1.29E-06
PE(P-18:1(11Z)/18:3(6Z,9Z,12Z))	Glycerophospholipids	0.01	-0.22	0.36	0.00	8.72E-01
beta-Alanine	Carboxylic acids and derivatives	0.38	0.10	0.51	0.15	1.96E-07
PC(22:2(13Z,16Z)/P-18:0)	Glycerophospholipids	0.02	-0.26	0.36	0.00	8.67E-01
DG(18:4(6Z,9Z,12Z,15Z)/16:1(9Z)/0:0)	Glycerolipids	0.58	0.19	0.67	0.94	1.59E-17
LysoPC(P-18:1(9Z))	Glycerophospholipids	0.09	-0.17	0.28	0.04	3.21E-01
MG(0:0/22:4(7Z,10Z,13Z,16Z)/0:0)	Glycerolipids	0.00	-0.18	0.20	0.00	9.81E-01
SM(d16:1/24:1(15Z))	Sphingolipids	0.09	-0.12	0.36	0.04	3.02E-01
4-Aminobutyraldehyde	Organooxygen compounds	0.17	-0.09	0.37	0.04	2.98E-02

1-(Malonylamino)cyclopropanecarboxylic acid	Carboxylic acids and derivatives	0.16	-0.35	0.40	0.09	5.21E-02
Quercetin	Flavonoids	0.19	-0.19	0.50	0.21	1.95E-02
N-Acetyl-a-neuraminic acid	Organooxygen compounds	-0.10	-0.28	0.17	0.02	2.29E-01
Ethyl trans-p-methoxycinnamate	Cinnamic acids and derivatives	0.09	-0.16	0.24	0.11	3.12E-01
4-Oxo-2-nonenal	Organooxygen compounds	0.02	-0.19	0.20	0.00	8.27E-01
3-Methyl sulfolene	Dihydrothiophenes	0.26	0.01	0.46	0.16	6.14E-04
PC(18:2(9Z,12Z)/18:0)	Glycerophospholipids	0.24	-0.09	0.62	0.11	1.48E-03
SM(d17:1/24:0)	Sphingolipids	-0.18	-0.42	0.19	0.11	2.45E-02
L-Octanoylcarnitine	Fatty Acyls	-0.12	-0.30	0.15	0.03	1.37E-01
gamma-Glutamylglutamic acid	Carboxylic acids and derivatives	-0.08	-0.49	0.43	0.03	3.52E-01
Ipomeatetrahydrofuran	Prenol lipids	0.56	0.13	0.70	0.72	4.45E-16
LysoPE(20:5(5Z,8Z,11Z,14Z,17Z)/0:0)	Glycerophospholipids	0.60	0.20	0.69	1.52	8.76E-19
Prolyl-Glutamine	Carboxylic acids and derivatives	-0.07	-0.60	0.38	0.01	4.25E-01
PC(22:5(7Z,10Z,13Z,16Z,19Z)/15:0)	Glycerophospholipids	0.02	-0.22	0.42	0.00	8.43E-01
PC(20:2(11Z,14Z)/14:0)	Glycerophospholipids	0.32	0.01	0.60	0.19	1.64E-05
PC(20:1(11Z)/14:0)	Glycerophospholipids	0.13	-0.17	0.44	0.05	1.24E-01
(R)-2-Hydroxysterculic acid	Fatty Acyls	0.48	0.09	0.62	0.81	2.02E-11

Fagomine	Piperidines	0.28	-0.10	0.51	0.12	2.22E-04
Isoleucyl-Alanine	Carboxylic acids and derivatives	-0.12	-0.32	0.11	0.02	1.69E-01
Glutamyllysine	Carboxylic acids and derivatives	-0.14	-0.31	0.12	0.18	7.77E-02
PC(15:0/15:0)	Glycerophospholipids	-0.06	-0.32	0.45	0.01	5.44E-01
MG(0:0/18:4(6Z,9Z,12Z,15Z)/0:0)	Glycerolipids	0.45	0.04	0.59	1.13	2.53E-10
N2-Maltulosylarginine	Saccharolipids	-0.17	-0.31	0.06	0.27	3.44E-02
PC(22:2(13Z,16Z)/16:1(9Z))	Glycerophospholipids	0.21	-0.12	0.63	0.10	7.00E-03
SM(d18:1/20:0)	Sphingolipids	0.08	-0.14	0.35	0.02	3.63E-01
Kojibiose	Fatty Acyls	-0.44	-0.64	0.15	0.59	1.22E-09
Cytokinin B	Imidazopyrimidines	-0.25	-0.50	0.02	0.18	1.22E-03
DG(18:4(6Z,9Z,12Z,15Z)/14:1(9Z)/0:0)	Glycerolipids	0.58	0.20	0.68	0.95	1.59E-17
PC(20:2(11Z,14Z)/20:4(5Z,8Z,11Z,14Z))	Glycerophospholipids	0.14	-0.17	0.52	0.06	9.96E-02
SM(d17:1/24:1(15Z))	Sphingolipids	-0.02	-0.27	0.37	0.00	8.39E-01
SM(d18:0/18:1(9Z))	Sphingolipids	0.06	-0.18	0.39	0.01	4.99E-01
5beta-Cholestane-3alpha,7alpha,24,26-tetrol	NA	-0.06	-0.30	0.23	0.01	4.98E-01
PC(22:5(4Z,7Z,10Z,13Z,16Z)/16:0)	Glycerophospholipids	0.30	-0.03	0.63	0.17	7.46E-05
PC(22:5(4Z,7Z,10Z,13Z,16Z)/P-18:0)	Glycerophospholipids	-0.03	-0.22	0.25	0.00	7.84E-01
PC(24:0/14:1(9Z))	Glycerophospholipids	0.18	-0.12	0.42	0.14	2.20E-02
LysoPC(16:0)	Glycerophospholipids	0.16	-0.12	0.34	0.10	4.80E-02

Sialyllacto-N-tetraose b	Organooxygen compounds	-0.18	-0.40	0.35	0.10	2.72E-02
SM(d18:1/14:0)	Sphingolipids	0.08	-0.14	0.41	0.03	3.56E-01
PI(22:5(4Z,7Z,10Z,13Z,16Z)/16:0)	Glycerophospholipids	0.01	-0.32	0.51	0.00	9.08E-01
Lactapiperanol D	Prenol lipids	0.10	-0.17	0.25	0.05	2.54E-01
L-Gulonolactone	Lactones	-0.19	-0.39	0.04	0.05	1.79E-02
2,6-Di-tert-butyl-4-ethylphenol	Benzene and substituted derivatives	0.37	0.02	0.49	0.67	3.34E-07
PC(18:3(6Z,9Z,12Z)/18:1(11Z))	Glycerophospholipids	0.24	0.00	0.44	0.23	2.25E-03
PC(22:4(7Z,10Z,13Z,16Z)/16:0)	Glycerophospholipids	0.24	-0.02	0.53	0.27	1.70E-03
PC(22:5(4Z,7Z,10Z,13Z,16Z)/15:0)	Glycerophospholipids	0.02	-0.29	0.53	0.00	8.63E-01
PC(14:0/14:0)	Glycerophospholipids	0.00	-0.27	0.46	0.00	9.81E-01
dADP	Purine nucleotides	-0.04	-0.25	0.11	0.00	6.81E-01
PC(20:1(11Z)/20:4(5Z,8Z,11Z,14Z))	Glycerophospholipids	0.18	-0.15	0.62	0.08	2.44E-02
3-Benzyl-4-heptanone	Benzene and substituted derivatives	0.53	0.18	0.64	0.91	1.81E-14
Betonicine	Carboxylic acids and derivatives	-0.19	-0.33	0.05	0.14	1.43E-02
Diepomuricanin A	NA	0.21	-0.04	0.43	0.10	7.85E-03
LysoPE(0:0/14:0)	Glycerophospholipids	0.39	0.03	0.55	1.00	8.72E-08

SM(d18:0/14:0)	Sphingolipids	0.13	-0.13	0.46	0.05	1.15E-01
PC(20:2(11Z,14Z)/15:0)	Glycerophospholipids	0.27	-0.07	0.64	0.17	3.30E-04
PC(22:2(13Z,16Z)/15:0)	Glycerophospholipids	0.32	-0.02	0.55	0.33	1.36E-05
2-(acetylamino)-1,5-anhydro-2-deoxy-3-O-b-D-galactopyranosyl-D-arabino-Hex-1-enitol	Organooxygen compounds	-0.04	-0.45	0.39	0.00	7.11E-01
PC(20:3(5Z,8Z,11Z)/20:0)	Glycerophospholipids	0.23	-0.07	0.48	0.28	2.63E-03
LysoPC(18:2(9Z,12Z))	Glycerophospholipids	0.57	0.16	0.71	1.18	3.83E-17
Urothion	Pteridines and derivatives	-0.13	-0.43	0.29	0.18	1.15E-01
(2'E,4'Z,7'Z,8E)-Colnelenic acid	Fatty Acyls	0.16	-0.10	0.28	0.22	4.80E-02
PC(20:0/20:2(11Z,14Z))	Glycerophospholipids	0.24	-0.06	0.48	0.35	1.56E-03
Glycerylphosphorylethanolamine	Organic phosphoric acids and derivatives	0.23	-0.09	0.46	0.20	2.90E-03
6,10,14-Trimethyl-5,9,13-pentadecatrien-2-one	Prenol lipids	0.40	0.02	0.50	0.64	3.20E-08
trans-3,4-Dihydro-4,8-dihydroxy-3-methyl-1H-2-benzopyran-1-one	Benzopyrans	-0.38	-0.57	0.09	0.27	1.60E-07
Pyridoxamine	Pyridines and derivatives	-0.02	-0.17	0.18	0.00	8.67E-01
PC(22:4(7Z,10Z,13Z,16Z)/14:0)	Glycerophospholipids	0.32	-0.02	0.61	0.24	2.19E-05
PE(20:0/18:4(6Z,9Z,12Z,15Z))	Glycerophospholipids	0.07	-0.20	0.46	0.01	4.35E-01

SM(d18:1/18:1(9Z))	Sphingolipids	-0.05	-0.29	0.37	0.01	6.22E-01
Cohibin C	Fatty Acyls	0.10	-0.25	0.56	0.03	2.34E-01
8-Hydroxy-2-octene-4,6-diynoic acid	Fatty Acyls	-0.26	-0.48	0.14	0.84	7.90E-04
5-Hydroxy-L-tryptophan	Indoles and derivatives	-0.01	-0.17	0.18	0.00	9.14E-01
PC(20:4(8Z,11Z,14Z,17Z)/P-18:0)	Glycerophospholipids	0.00	-0.21	0.29	0.00	9.81E-01
Phenylacetylglutamine	Carboxylic acids and derivatives	-0.22	-0.35	0.06	0.11	4.99E-03
Isoeugenitol	Benzopyrans	0.27	-0.03	0.43	0.13	3.95E-04
PC(16:1(9Z)/16:1(9Z))	Glycerophospholipids	0.18	-0.07	0.47	0.10	2.57E-02
gamma-L-Glutamyl-gamma-L-glutamyl-L-methionine		-0.32	-0.46	-0.03	0.45	1.98E-05
3-Oxoadipic acid	Keto acids and derivatives	-0.22	-0.41	0.06	0.05	4.92E-03
6-Deoxohomodolichosterone	Steroids and steroid derivatives	-0.08	-0.33	0.28	0.02	3.43E-01
PC(18:1(9Z)/P-18:1(11Z))	Glycerophospholipids	0.09	-0.14	0.32	0.02	2.82E-01
N-Salicyloylaspartic acid	Carboxylic acids and derivatives	0.13	-0.19	0.30	0.02	1.08E-01
Vanilpyruvic acid	Benzene and substituted derivatives	-0.11	-0.28	0.07	0.02	1.76E-01
PC(22:5(7Z,10Z,13Z,16Z,19Z)/16:1(9Z))	Glycerophospholipids	0.18	-0.14	0.58	0.08	2.44E-02
LysoPC(22:6(4Z,7Z,10Z,13Z,16Z,19Z))	Glycerophospholipids	0.34	-0.02	0.52	0.39	4.58E-06

Norsanguinarine	Quinolines and derivatives	0.13	-0.14	0.28	0.04	1.15E-01
3-Methyl-5-pentyl-2-furanundecanoic acid	Fatty Acyls	0.49	0.09	0.57	0.67	5.50E-12
3-Aminopropionaldehyde	Organooxygen compounds	0.30	-0.01	0.45	0.16	6.46E-05
PC(22:5(7Z,10Z,13Z,16Z,19Z)/16:0)	Glycerophospholipid s	0.25	0.01	0.52	0.26	1.22E-03
PC(20:3(8Z,11Z,14Z)/14:0)	Glycerophospholipid s	0.34	0.05	0.50	0.42	3.95E-06
Dihydromaleimide	NA	0.21	-0.08	0.33	0.10	8.76E-03
2,5-Dioxopentanoate	Keto acids and derivatives	-0.10	-0.27	0.08	0.01	2.56E-01
CS-S-Methylcysteine sulfoxide	NA	0.19	-0.22	0.50	0.35	1.50E-02
Oxonantenine	Aporphines	-0.15	-0.52	0.42	0.04	6.38E-02
DG(14:1(9Z)/18:3(6Z,9Z,12Z)/0:0)	Glycerolipids	0.60	0.16	0.73	0.93	7.09E-19
2-Acetylpyridine	Carbonyl compounds	0.05	-0.15	0.23	0.00	5.73E-01
2-Hydroxy-6,7-dimethoxybenzoxazole	Benzoxazoles	-0.52	-0.61	-0.18	0.47	3.85E-14
Perillyl acetate	NA	0.06	-0.23	0.24	0.02	5.17E-01
N-[(4E,8E)-1,3-dihydroxyoctadeca-4,8-dien-2-yl]hexadecanamide	Sphingolipids	-0.10	-0.32	0.12	0.04	2.56E-01
Dihydro-3-(1-octenyl)-2,5-furandione	Oxolanes	-0.05	-0.20	0.13	0.01	6.22E-01
Artonin Q	Benzopyrans	-0.08	-0.35	0.25	0.02	3.93E-01
2-Aminomuconic acid semialdehyde	Carboxylic acids and derivatives	0.04	-0.18	0.25	0.01	6.75E-01
DG(20:4(8Z,11Z,14Z,17Z)/14:0/0:0)	Glycerolipids	0.54	0.17	0.68	0.83	3.27E-15
Lacto-N-triose I	Organooxygen compounds	-0.02	-0.45	0.42	0.00	8.39E-01

PC(P-18:1(9Z)/14:1(9Z))	Glycerophospholipids	0.02	-0.19	0.23	0.00	8.55E-01
(2S,4S)-Pinnatanine	Carboxylic acids and derivatives	-0.04	-0.33	0.27	0.00	6.75E-01
O-6-deoxy-a-L-galactopyranosyl-(1->2)-O-b-D-galactopyranosyl-(1->3)-2-(acetylamino)-1,5-anhydro-2-deoxy-D-arabino-Hex-1-enitol	Organooxygen compounds	-0.25	-0.44	0.12	0.18	1.34E-03
Persicachrome	Prenol lipids	0.42	0.01	0.62	0.60	8.73E-09
L-trans-5-Hydroxy-2-piperidinecarboxylic acid	Carboxylic acids and derivatives	-0.08	-0.26	0.14	0.01	3.93E-01
2-acetyl-1-alkyl-sn-glycero-3-phosphocholine	Glycerophospholipids	0.19	-0.11	0.46	0.13	1.59E-02
PC(16:1(9Z)/15:0)	Glycerophospholipids	0.12	-0.24	0.58	0.04	1.39E-01
LysoPC(P-16:0)	Glycerophospholipids	-0.06	-0.24	0.14	0.01	5.17E-01
Phenol glucuronide	Organooxygen compounds	-0.35	-0.48	-0.03	0.41	2.08E-06
PC(18:3(6Z,9Z,12Z)/15:0)	Glycerophospholipids	0.18	-0.06	0.39	0.09	2.59E-02
PI(20:2(11Z,14Z)/18:2(9Z,12Z))	Glycerophospholipids	-0.14	-0.45	0.44	0.06	9.83E-02
Glycine	Carboxylic acids and derivatives	0.35	0.08	0.47	0.21	3.36E-06
DG(14:1(9Z)/18:2(9Z,12Z)/0:0)	Glycerolipids	0.56	0.11	0.70	0.83	2.22E-16
Epomusenin A	Fatty Acyls	-0.08	-0.32	0.30	0.01	3.76E-01
Troxerutin	Flavonoids	0.30	-0.24	0.60	0.41	7.53E-05

LysoPE(20:2(11Z,14Z)/0:0)	Glycerophospholipids	0.58	-0.03	0.81	1.72	6.53E-18
PE(P-18:1(11Z)/22:4(7Z,10Z,13Z,16Z))	Glycerophospholipids	0.10	-0.13	0.41	0.03	2.60E-01
PC(22:4(7Z,10Z,13Z,16Z)/15:0)	Glycerophospholipids	-0.01	-0.31	0.48	0.00	9.57E-01
1-Methylpyrrole	Pyrroles	0.03	-0.15	0.17	0.00	7.94E-01
LysoPC(22:5(4Z,7Z,10Z,13Z,16Z))	Glycerophospholipids	0.40	0.02	0.57	1.08	2.39E-08
5-Hydroxyindoleacetic acid	Indoles and derivatives	0.05	-0.15	0.23	0.01	5.73E-01
Seryltyrosine	Carboxylic acids and derivatives	-0.10	-0.22	0.05	0.01	2.41E-01
5-Hydroxyisourate		0.29	0.04	0.44	1.61	9.70E-05
Lacto-N-biose I	Organooxygen compounds	0.19	-0.12	0.38	0.08	1.45E-02
PE(P-18:1(11Z)/18:2(9Z,12Z))	Glycerophospholipids	0.21	0.00	0.51	0.16	8.26E-03
3-(3,4-Dihydroxy-5-methoxy)-2-propenoic acid	Cinnamic acids and derivatives	-0.05	-0.26	0.20	0.00	6.24E-01
4-Hydroxy-3-(16-methylheptadecyl)-2H-pyran-2-one	Pyrans	0.30	0.00	0.40	0.38	5.32E-05
LysoPC(20:3(5Z,8Z,11Z))	Glycerophospholipids	0.31	-0.07	0.56	0.34	3.74E-05
PI(20:3(5Z,8Z,11Z)/16:0)	Glycerophospholipids	0.17	-0.12	0.47	0.13	3.90E-02
1-(3,5-Dihydroxyphenyl)-2-pentadecanone	Phenols	0.34	0.09	0.49	0.60	4.99E-06

Glucocheirolin	Organooxygen compounds	0.31	-0.06	0.55	0.37	3.78E-05
PE(18:1(9Z)/16:0)	Glycerophospholipids	0.15	-0.11	0.51	0.07	7.39E-02
5-Hydroxy-7-methoxy-2-tritriacontyl-4H-1-benzopyran-4-one	Benzopyrans	0.04	-0.18	0.27	0.01	6.79E-01
DG(14:1(9Z)/22:5(4Z,7Z,10Z,13Z,16Z)/0:0)	Glycerolipids	0.34	0.02	0.49	1.07	5.67E-06
12,13-EpOME	Fatty Acyls	0.32	-0.06	0.45	0.41	2.45E-05
PE(20:4(8Z,11Z,14Z,17Z)/P-16:0)	Glycerophospholipids	-0.02	-0.29	0.37	0.00	8.52E-01
3,5-Dichloro-4-hydroxy-2-methoxy-6-methylbenzoic acid	Benzene and substituted derivatives	0.06	-0.17	0.20	0.00	5.50E-01
Hydroxyphenylacetylglycine	Carboxylic acids and derivatives	0.03	-0.32	0.28	0.00	7.12E-01
DG(22:4(7Z,10Z,13Z,16Z)/18:3(6Z,9Z,12Z)/0:0)	Glycerolipids	0.24	-0.05	0.35	0.31	2.28E-03
PC(22:6(4Z,7Z,10Z,13Z,16Z,19Z)/15:0)	Glycerophospholipids	0.00	-0.28	0.43	0.00	9.89E-01
PS(14:0/22:2(13Z,16Z))	Glycerophospholipids	-0.23	-0.54	0.45	0.18	3.59E-03
PC(20:4(8Z,11Z,14Z,17Z)/15:0)	Glycerophospholipids	0.10	-0.13	0.46	0.04	2.57E-01
Epiafzelechin 3-gallate		0.10	-0.43	0.61	0.05	2.60E-01
PE(18:1(11Z)/18:2(9Z,12Z))	Glycerophospholipids	0.16	-0.05	0.46	0.11	5.32E-02
PE(P-18:1(9Z)/16:1(9Z))	Glycerophospholipids	0.02	-0.26	0.40	0.00	8.67E-01
Demethylated antipyrine	Azoles	0.21	-0.01	0.35	0.13	6.57E-03

PE(P-18:1(11Z)/22:5(4Z,7Z,10Z,13Z,16Z))	Glycerophospholipids	0.06	-0.19	0.43	0.01	4.99E-01
3-Fucosyllactose	Organooxygen compounds	0.18	-0.23	0.46	0.13	2.09E-02
LysoPI(18:0/0:0)	Glycerophospholipids	-0.06	-0.29	0.20	0.01	4.81E-01
Isolinderanolide	Tetrahydrofurans	0.62	0.24	0.73	1.46	1.41E-20
PE(P-18:1(9Z)/20:3(5Z,8Z,11Z))	Glycerophospholipids	-0.05	-0.30	0.36	0.01	5.80E-01
Marmesin rhamnoside	Coumarins and derivatives	-0.12	-0.36	0.15	0.02	1.60E-01
1-Isothiocyanato-6-(methylthio)hexane	Isothiocyanates	0.14	-0.13	0.30	0.04	7.55E-02
PE(P-18:1(9Z)/20:5(5Z,8Z,11Z,14Z,17Z))	Glycerophospholipids	-0.07	-0.32	0.39	0.01	4.11E-01
PC(P-16:0/18:4(6Z,9Z,12Z,15Z))	Glycerophospholipids	-0.08	-0.28	0.29	0.02	3.49E-01
Obtusilactone A	Tetrahydrofurans	0.55	0.18	0.65	0.72	9.37E-16
PE(P-18:1(9Z)/18:1(9Z))	Glycerophospholipids	0.03	-0.21	0.41	0.00	7.19E-01
PE(22:6(4Z,7Z,10Z,13Z,16Z,19Z)/P-18:1(11Z))	Glycerophospholipids	0.14	-0.17	0.49	0.04	9.95E-02
PE(20:4(5Z,8Z,11Z,14Z)/P-18:1(11Z))	Glycerophospholipids	0.16	-0.15	0.52	0.04	5.19E-02
Cohibin B	Fatty Acyls	0.14	-0.07	0.38	0.04	9.82E-02
Dide-O-methyl-4-O-alpha-D-glucopyranosylsimmondsin	Organooxygen compounds	-0.05	-0.30	0.28	0.01	5.73E-01
PC(P-18:1(9Z)/16:1(9Z))	Glycerophospholipids	0.00	-0.18	0.16	0.00	9.76E-01

1-Isothiocyanato-6-(methylsulfinyl)hexane	Sulfoxides	0.00	-0.16	0.17	0.00	9.93E-01
PC(20:3(8Z,11Z,14Z)/15:0)	Glycerophospholipids	0.04	-0.28	0.53	0.00	6.97E-01
3-Isopropenylpentanedioic acid	Fatty Acyls	-0.23	-0.62	0.22	0.05	2.58E-03
PE(P-16:0/20:4(5Z,8Z,11Z,14Z))	Glycerophospholipids	-0.08	-0.32	0.40	0.01	3.68E-01
Glaucarubin	Prenol lipids	0.09	-0.24	0.34	0.05	3.27E-01
PE(22:2(13Z,16Z)/18:1(11Z))	Glycerophospholipids	0.20	-0.17	0.58	0.11	1.24E-02
PE(P-18:0/18:2(9Z,12Z))	Glycerophospholipids	0.02	-0.25	0.39	0.00	8.63E-01
Melleolide M	Prenol lipids	0.12	-0.16	0.34	0.11	1.38E-01
PE(P-18:0/22:4(7Z,10Z,13Z,16Z))	Glycerophospholipids	-0.07	-0.32	0.35	0.01	4.16E-01
Metiamide	Azoles	0.46	-0.10	0.60	0.58	7.13E-11
PS(18:0/22:6(4Z,7Z,10Z,13Z,16Z,19Z))	Glycerophospholipids	-0.19	-0.49	0.43	0.18	1.43E-02

Table S6. Association between human milk PC scores, infant growth and risk of eczema by sex (n=200)

		Boys (n=114)				Girls (n=86)			
		PC 1		PC 2		PC 1		PC 2	
		Estimates	P value	Estimates	P value	Estimates	P value	Estimates	P value
Maternal BMI (kg/m^2)	Model 1	-0.05	0.345	0.08	0.086	-0.01	0.926	0.08	0.086
	Model 2	-0.05	0.347	0.08	0.109	-0.003	0.960	0.08	0.093
BAZ	Model 1	-0.03	0.501	0.001	0.869	-0.02	0.637	-0.01	0.890
	Model 2	-0.03	0.397	-0.02	0.518	-0.03	0.517	-0.01	0.817
WAZ	Model 1	-0.08	0.017	0.02	0.443	-0.05	0.171	0.00	0.966
	Model 2	-0.07	0.018	2.0E-03	0.945	-0.05	0.083	7.0E-03	0.795
LAZ	Model 1	-0.11	0.004	0.025	0.424	-0.06	0.154	0.006	0.879
	Model 2	-0.07	0.012	0.025	0.418	-0.06	0.176	0.019	0.592
Eczema	Model 1	0.95	0.279	1.07	0.078	0.94	0.340	1.07	0.133
	Model 2	0.96	0.392	1.08	0.048	0.95	0.455	1.06	0.224

Estimates for allergies were presented as odds ratio.Model 1 adjusted for infant age, sex, maternal age and city; model 2 adjusted for infant age, sex, city, maternal age, birth weight and birth length.

WAZ: weight-for-age *z* score, LAZ: length-for-age *z* score, BAZ: BMI-for-age *z* score

Table S7. Differentially expressed human milk metabolites between samples collected in summer and winter (n=77)

Metabolites	Summer (n=20) Mean	Summer (n=20) SD	Winter (n=57) Mean	Winter (n=57) SD	Fold change	FDR
Solasodine	8.94E-07	2.27E-06	4.26E-06	6.12E-06	0.21	6.31E-03
N2-gamma-Glutamylglutamine	2.63E-05	1.82E-05	1.13E-04	8.18E-05	0.23	1.42E-08
MG(0:0/22:4(7Z,10Z,13Z,16Z)/0:0)	8.31E-06	6.36E-06	3.20E-05	1.72E-05	0.26	1.68E-10
5beta-Cholestane-3alpha,7alpha,24,26-tetrol	8.95E-06	7.65E-06	2.76E-05	1.20E-05	0.32	1.22E-08
N2-Maltulosylarginine	1.95E-06	1.91E-06	5.69E-06	6.54E-06	0.34	2.33E-03
Biliverdin	2.92E-05	4.11E-05	8.29E-05	8.35E-05	0.35	3.71E-03
Betonicine	3.96E-05	2.59E-05	1.09E-04	1.65E-04	0.36	2.28E-02
PE(16:0/20:4(5Z,8Z,11Z,14Z))	5.09E-05	2.45E-05	1.39E-04	8.45E-05	0.37	3.90E-08
6-Deoxohomodolichosterone	8.21E-06	7.03E-06	2.21E-05	1.14E-05	0.37	1.09E-06
gamma-Glutamylglutamic acid	2.70E-06	2.01E-06	7.26E-06	6.12E-06	0.37	9.07E-05
CS-S-Methylcysteine sulfoxide	2.03E-05	1.86E-05	5.33E-05	5.23E-05	0.38	1.25E-03
Melleolide M	4.47E-06	3.22E-06	1.05E-05	6.26E-06	0.43	1.90E-05
Anserine	9.98E-06	5.09E-06	2.29E-05	2.29E-05	0.44	1.75E-03
5-Hydroxy-7-methoxy-2-tritriacontyl-4H-1-benzopyran-4-one	1.49E-05	7.69E-06	3.38E-05	1.51E-05	0.44	4.25E-08
PC(18:3(6Z,9Z,12Z)/15:0)	4.33E-04	1.69E-04	9.43E-04	3.64E-04	0.46	1.22E-09
PE(18:1(11Z)/18:2(9Z,12Z))	1.18E-04	4.48E-05	2.58E-04	1.10E-04	0.46	4.21E-09
LysoPE(0:0/18:3(6Z,9Z,12Z))	1.10E-05	7.00E-06	2.38E-05	2.74E-05	0.46	1.36E-02
PI(20:3(5Z,8Z,11Z)/16:0)	2.70E-06	1.25E-06	5.83E-06	3.09E-06	0.46	5.72E-07
gamma-L-Glutamyl-gamma-L-glutamyl-L-methionine	7.00E-06	4.60E-06	1.50E-05	1.21E-05	0.47	8.80E-04
Glaucarubin	1.01E-05	8.79E-06	2.14E-05	1.90E-05	0.47	5.92E-03
Urocanic acid	1.29E-04	1.22E-04	2.71E-04	2.44E-04	0.48	1.01E-02
Sonchuionoside C	2.64E-05	2.04E-05	5.32E-05	2.48E-05	0.50	3.53E-04
PE(22:5(4Z,7Z,10Z,13Z,16Z)/16:0)	6.40E-05	2.66E-05	1.24E-04	4.33E-05	0.52	5.83E-08
PC(20:3(8Z,11Z,14Z)/14:0)	5.41E-05	1.93E-05	1.03E-04	8.67E-05	0.53	1.75E-03
4-Hydroxy-L-glutamic acid	1.55E-04	1.42E-04	2.95E-04	1.20E-04	0.53	4.50E-03

PC(16:1(9Z)/16:1(9Z))	3.42E-05	2.23E-05	6.46E-05	3.08E-05	0.53	3.24E-04
Pyroglutamic acid	3.53E-05	3.11E-05	6.62E-05	3.13E-05	0.53	4.94E-03
5'-Methylthioadenosine	6.61E-04	1.62E-04	1.22E-03	4.72E-04	0.54	4.85E-09
Adenine	8.16E-05	7.25E-05	1.48E-04	7.84E-05	0.55	1.08E-02
5-Hydroxyisourate	1.77E-04	1.79E-04	3.13E-04	1.63E-04	0.57	3.45E-02
MG(22:2(13Z,16Z)/0:0/0:0)	8.03E-05	5.43E-05	1.36E-04	7.33E-05	0.59	6.77E-03
PC(14:0/14:0)	1.14E-05	8.23E-06	1.89E-05	1.44E-05	0.60	3.71E-02
SM(d18:0/14:0)	2.20E-05	9.98E-06	3.41E-05	2.66E-05	0.64	3.13E-02
PS(14:0/22:2(13Z,16Z))	1.01E-04	5.29E-05	1.57E-04	1.06E-04	0.65	2.44E-02
D-Glutamine	1.03E-02	5.13E-03	1.55E-02	5.51E-03	0.66	4.63E-03
Styrene	8.62E-05	2.23E-05	5.53E-05	2.61E-05	1.56	1.55E-04
Indole	2.36E-04	5.58E-05	1.36E-04	6.42E-05	1.74	1.97E-06
LysoPC(22:6(4Z,7Z,10Z,13Z,16Z,19Z))	1.57E-05	1.01E-05	8.91E-06	6.40E-06	1.77	4.95E-02
Hypoxanthine	3.89E-05	2.19E-05	2.20E-05	1.97E-05	1.77	3.13E-02
(R)-2-Hydroxysterculic acid	3.75E-04	1.08E-04	2.04E-04	1.00E-04	1.84	1.54E-05
Niacinamide	3.63E-03	1.65E-03	1.93E-03	1.25E-03	1.88	2.54E-03
N1-Methyl-4-pyridone-3-carboxamide	1.66E-03	9.35E-04	8.48E-04	5.21E-04	1.95	1.01E-02
Diethanolamine	1.45E-04	1.15E-04	6.98E-05	2.29E-05	2.08	4.87E-02
o-Ethyltoluene	8.10E-05	1.92E-05	3.69E-05	2.23E-05	2.19	2.00E-08
Perillyl acetate	4.10E-05	2.58E-05	1.85E-05	4.46E-05	2.22	4.85E-02
Epsilon-caprolactam	1.06E-04	6.31E-05	4.74E-05	2.83E-05	2.23	5.44E-03
PC(P-18:1(9Z)/16:1(9Z))	5.45E-05	1.97E-05	2.36E-05	2.17E-05	2.31	2.05E-05
Avocadyne 4-acetate	2.50E-04	1.35E-04	1.03E-04	4.81E-05	2.42	1.25E-03
3-Methyl-5-pentyl-2-furanundecanoic acid	1.34E-03	3.69E-04	5.30E-04	2.17E-04	2.53	8.07E-08
LysoPE(20:1(11Z)/0:0)	8.30E-05	4.16E-05	3.25E-05	2.51E-05	2.55	4.14E-04
(9S,10E,12Z,15Z)-9-Hydroxy-10,12,15-octadecatrienoic acid	6.87E-05	4.07E-05	2.46E-05	2.88E-05	2.79	1.47E-03
12,13-EpOME	3.00E-05	1.40E-05	9.96E-06	7.31E-06	3.01	7.03E-05
DG(22:5(7Z,10Z,13Z,16Z,19Z)/16:1(9Z)/0:0)	2.42E-05	1.16E-05	7.83E-06	7.15E-06	3.09	7.19E-05

Quinceoxepine	5.48E-05	2.74E-05	1.71E-05	1.91E-05	3.22	9.69E-05
MG(0:0/18:4(6Z,9Z,12Z,15Z)/0:0)	1.26E-04	7.50E-05	3.86E-05	4.46E-05	3.25	7.21E-04
LysoPE(0:0/22:1(13Z))	3.71E-05	3.41E-05	1.11E-05	1.70E-05	3.35	2.38E-02
Dihydro-6-isopropyl-2,4-dimethyl-4H-1,3,5-dithiazine	3.09E-05	1.66E-05	9.10E-06	1.12E-05	3.40	1.73E-04
13S-hydroxyoctadecadienoic acid	1.09E-05	6.02E-06	3.18E-06	3.86E-06	3.44	2.14E-04
4-Hydroxy-3-(16-methylheptadecyl)-2H-pyran-2-one	2.14E-05	8.07E-06	6.15E-06	6.10E-06	3.49	7.75E-07
6,10,14-Trimethyl-5,9,13-pentadecatrien-2-one	3.40E-04	1.18E-04	9.55E-05	6.90E-05	3.56	2.59E-07
5,12-dihydroxy-6,8,10,14,17-eicosapentaenoic acid	1.94E-05	1.14E-05	5.13E-06	7.87E-06	3.78	3.11E-04
Alpha-dimorphecolic acid	9.44E-04	3.34E-04	2.38E-04	3.44E-04	3.96	8.07E-08
2,6-Di-tert-butyl-4-ethylphenol	9.14E-05	4.20E-05	2.29E-05	2.23E-05	3.98	1.14E-05
Corchorifatty acid D	2.70E-05	1.77E-05	6.49E-06	1.63E-05	4.15	9.25E-04
DG(22:4(7Z,10Z,13Z,16Z)/18:3(6Z,9Z,12Z)/0:0)	2.37E-05	1.13E-05	5.60E-06	5.64E-06	4.24	1.54E-05
Nandrolone	5.93E-05	5.43E-05	1.38E-05	4.88E-05	4.29	1.73E-02
4,8 Dimethylnonanoyl carnitine	4.73E-05	3.69E-05	9.86E-06	2.32E-05	4.80	2.63E-03
(2'E,4'Z,7'Z,8E)-Colnelenic acid	1.68E-04	1.57E-04	3.25E-05	1.09E-04	5.17	1.08E-02
Arachidonic acid	4.52E-05	1.68E-05	7.50E-06	1.37E-05	6.03	4.03E-08
Delta-12-Prostaglandin J2	1.77E-05	1.29E-05	1.79E-06	7.49E-06	9.85	3.53E-04

Results were based on independent sample t-test between samples collected in summer and in winter. Fold change was calculated as summer vs. winter.

Table S8. Overlapped human milk metabolites being identified from both PC 1 and t-test

Metabolites	PC1 loading	Fold change	FDR
N2-gamma-Glutamylglutamine	-0.75	0.23	1.42E-08
MG(0:0/22:4(7Z,10Z,13Z,16Z)/0:0)	-0.69	0.26	1.68E-10
5beta-Cholestane-3alpha,7alpha,24,26-tetrol	-0.56	0.32	1.22E-08
Biliverdin	-0.83	0.35	3.71E-03
PE(16:0/20:4(5Z,8Z,11Z,14Z))	-0.56	0.37	3.90E-08
Melleolide M	-0.75	0.43	1.90E-05
5-Hydroxy-7-methoxy-2-tritriacontyl-4H-1-benzopyran-4-one	-0.56	0.44	4.25E-08
PC(18:3(6Z,9Z,12Z)/15:0)	-0.64	0.46	1.22E-09
Glaucarubin	-0.77	0.47	5.92E-03
Sonchuionoside C	-0.69	0.50	3.53E-04
PE(22:5(4Z,7Z,10Z,13Z,16Z)/16:0)	-0.55	0.52	5.83E-08
4-Hydroxy-L-glutamic acid	-0.87	0.53	4.50E-03
Pyroglutamic acid	-0.67	0.53	4.94E-03
5'-Methylthioadenosine	-0.57	0.54	4.85E-09
Adenine	-0.56	0.55	1.08E-02
5-Hydroxyisourate	-0.73	0.57	3.45E-02
D-Glutamine	-0.73	0.66	4.63E-03
Styrene	0.74	1.56	1.55E-04
Indole	0.79	1.74	1.97E-06
Niacinamide	0.56	1.88	2.54E-03
o-Ethyltoluene	0.77	2.19	2.00E-08
Perillyl acetate	0.82	2.22	4.85E-02
3-Methyl-5-pentyl-2-furanundecanoic acid	0.69	2.53	8.07E-08
(9S,10E,12Z,15Z)-9-Hydroxy-10,12,15-octadecatrienoic acid	0.80	2.79	1.47E-03
12,13-EpOME	0.83	3.01	7.03E-05
DG(22:5(7Z,10Z,13Z,16Z,19Z)/16:1(9Z)/0:0)	0.69	3.09	7.19E-05
Quinceoxepine	0.86	3.22	9.69E-05

MG(0:0/18:4(6Z,9Z,12Z,15Z)/0:0)	0.71	3.25	7.21E-04
Dihydro-6-isopropyl-2,4-dimethyl-4H-1,3,5-dithiazine	0.83	3.40	1.73E-04
13S-hydroxyoctadecadienoic acid	0.85	3.44	2.14E-04
4-Hydroxy-3-(16-methylheptadecyl)-2H-pyran-2-one	0.76	3.49	7.75E-07
6,10,14-Trimethyl-5,9,13-pentadecatrien-2-one	0.79	3.56	2.59E-07
5,12-dihydroxy-6,8,10,14,17-eicosapentaenoic acid	0.69	3.78	3.11E-04
Alpha-dimorphecolic acid	0.83	3.96	8.07E-08
2,6-Di-tert-butyl-4-ethylphenol	0.76	3.98	1.14E-05
Corchorifatty acid D	0.87	4.15	9.25E-04
DG(22:4(7Z,10Z,13Z,16Z)/18:3(6Z,9Z,12Z)/0:0)	0.71	4.24	1.54E-05
Nandrolone	0.82	4.29	1.73E-02
4,8 Dimethylnonanoyl carnitine	0.87	4.80	2.63E-03
(2'E,4'Z,7'Z,8E)-Colnelenic acid	0.88	5.17	1.08E-02
Arachidonic acid	0.87	6.03	4.03E-08
Delta-12-Prostaglandin J2	0.84	9.85	3.53E-04

Fold change was calculated as summer vs. winter.

Table S9. Overlapped human milk metabolites being identified from both PC 2 and t-test

Metabolites	PC2 loading	Fold change	FDR
PE(16:0/20:4(5Z,8Z,11Z,14Z))	0.54	0.37	6.44E-16
PE(22:5(4Z,7Z,10Z,13Z,16Z)/16:0)	0.61	0.52	4.02E-21
PC(14:0/14:0)	0.61	0.60	4.02E-21
SM(d18:0/14:0)	0.65	0.64	7.15E-24
PC(16:1(9Z)/16:1(9Z))	0.69	0.53	1.25E-28
6-Deoxohomodolichosterone	0.54	0.37	2.08E-15
PC(20:3(8Z,11Z,14Z)/14:0)	0.52	0.53	1.06E-14
PC(18:3(6Z,9Z,12Z)/15:0)	0.57	0.46	1.27E-17
PI(20:3(5Z,8Z,11Z)/16:0)	0.55	0.46	4.55E-16
PS(14:0/22:2(13Z,16Z))	0.57	0.65	2.04E-17

Fold change was calculated as summer vs. winter.

Table S10 Association between weight of milk collected, PC scores, ambient temperature and infant z scores (n = 200).

	Estimates	P value
**PC1 score**	**-0.51**	**0.007**
**PC2 score**	**0.13**	**0.552**
**Ambient temperature**	**-0.92**	**0.022**
**WAZ**	**1.86**	**0.032**
**LAZ**	**0.94**	**0.210**

Multiple linear regression adjusted for infant age, sex, maternal age and city.

WAZ: weight-for-age *z* score, LAZ: length-for-age *z* score

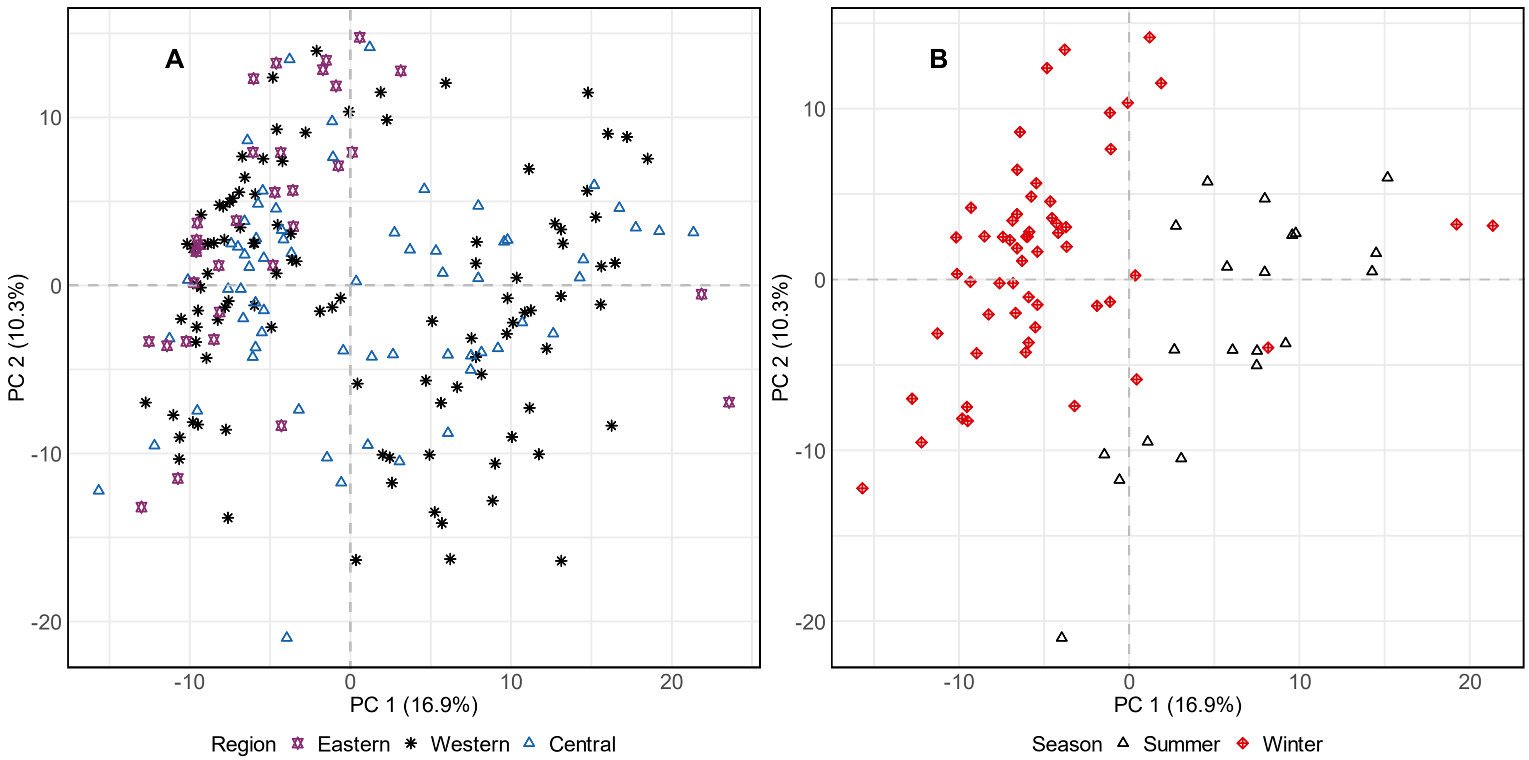

Figure S1. PCA score plot of human milk metabolites.

A: PCA score plot across three regions of China. The Eastern includes Beijing, Guangzhou, Jinhua and Weihai; the Western includes Chengdu and Lanzhou, the Central includes Zhengzhou.

B: PCA score plot between summer and winter.

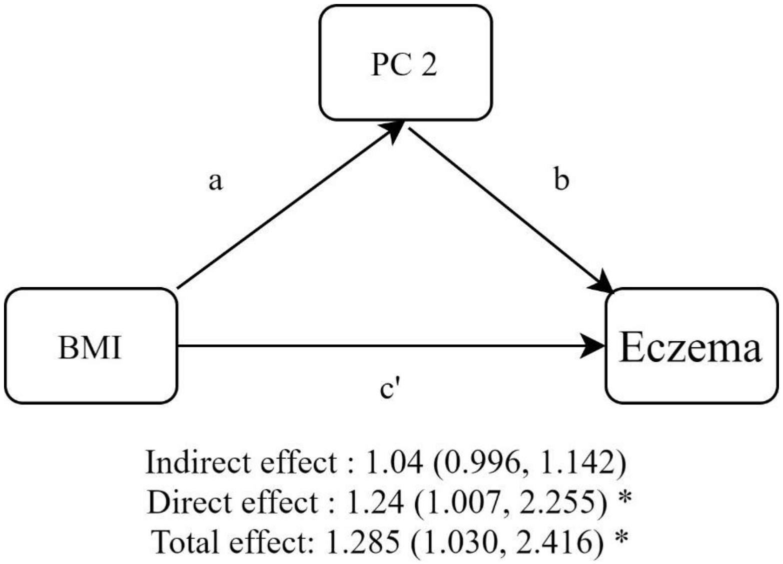

Figure S2. Mediation analysis between human milk PC scores, maternal postpartum BMI and risk of allergies.
Indirect effect, direct effect and total effect were defined as a×b, c′ and a×b+c′, respectively. All the effects were expressed as estimate (95% bootstrap confidence interval). * indicates significant effect based on 95% bootstrap CI. All the models were adjusted for infant age, infant sex, city, and maternal age.
PC 2: score of principal component 2; BMI: maternal postpartum BMI. ºº

2. Materials and Methods

2.4. Untargeted metabolomic analysis

2.4.1. Metabolites extraction

Metabolites were extracted by mixing 100 μL of sample and 400 μL of extract solution (acetonitrile: methanol=1:1, containing mixture of three isotopically labelled internal standards). The internal standards were carefully selected to avoid any potential intervene with HM endogenous metabolism. The mixture was vortexed for 30 s, sonicated for 10 min in ice-water bath, and incubated for 1 h at -40 ℃ to precipitate proteins. The mixture was then centrifuged at 12000 rpm for 15 min at 4 ℃. The resulting supernatant was transferred to a fresh glass vial for analysis. Quality control samples (21 in total) were prepared by mixing an equal aliquot of the supernatants from all the samples.

2.4.2. LC-MS/MS Analysis

LC-MS/MS analyses were performed using an UHPLC system (Vanquish, Thermo Fisher Scientific) with a UPLC BEH Amide column (2.1 mm × 100 mm, 1.7 μm) coupled to Q Exactive HFX mass spectrometer (Orbitrap MS, Thermo). The mobile phase consisted of 25 mmol/L ammonium acetate and 25 ammonia hydroxide in water (pH = 9.75) (A) and acetonitrile (B). The auto-sampler temperature was 4 ℃, and the injection volume was 3 μL. The QE HFX mass spectrometer was used for its ability to acquire MS/MS spectra on information-dependent acquisition (IDA) mode in the control of the acquisition software (Xcalibur, Thermo). In this mode, the acquisition software continuously evaluates the full scan MS spectrum. The ESI source conditions were set as following: sheath gas flow rate as 30 Arb, Aux gas flow rate as 25 Arb, capillary temperature 350 ℃, full MS resolution as 60000, MS/MS resolution as 7500, collision energy as 10/30/60 in NCE mode, spray Voltage as 3.6 kV (positive) or -3.2 kV (negative), respectively.

2.4.3. Data preprocessing and annotation

The raw data were converted to the mzXML format using ProteoWizard and processed with an in-house program, which was developed using R and based on XCMS, for peak detection, extraction, alignment, and integration. Then an in-house MS2 database built with over 2,000 chemical standards was applied in metabolite annotation. The similarity score of MS/MS spectra was calculated by using the forward dot-product algorithm [10]. The cutoff for annotation was set at 0.3.
